# Silver-Catalyzed Cascade Cyclization of Amino-*NH*-1,2,3-Triazoles with 2-Alkynylbenzaldehydes: An Access to Pentacyclic Fused Triazoles

**DOI:** 10.3390/molecules27217567

**Published:** 2022-11-04

**Authors:** Shuitao Zhang, Jianxin Li, Tiebo Xiao, Baomin Yang, Yubo Jiang

**Affiliations:** Faculty of Science, Kunming University of Science and Technology, South Jingming Road 727, Chenggong District, Kunming 650500, China

**Keywords:** cascade, cyclizations, fused 1,2,3-triazoles, isoquinoline, quinazoline

## Abstract

An operationally simple Ag(I)-catalyzed approach for the synthesis of isoquinoline and quinazoline fused 1,2,3-triazoles was developed by a condensation and amination cyclization cascade of amino-*NH*-1,2,3-triazoles with 2-alkynylbenzaldehydes involving three new *C*-*N* bond formations in one manipulation, in which the group of -*NH* of the triazole ring serves as a nucleophile to form the quinazoline skeleton. The efficient protocol can be applied to a variety of substrates containing a range of functional groups, delivering novel pentacyclic fused 1,2,3-triazoles in good-to-excellent yields.

## 1. Introduction

**Figure 1:**
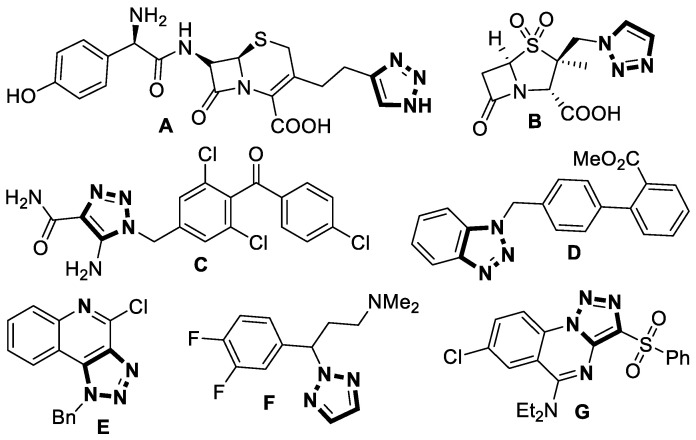
Some 1,2,3-triazole-based drugs and bioactive molecules.

Broad attention has been paid to 1,2,3-triazole-containing heterocycles, which have been widely applied in the fields of medicine [1,2,3,4,5], pesticide [6,7,8,9,10], biochemistry [11,12,13,14], and material science [15,16,17,18,19,20] since the ‘Click’ triazole chemistry was founded at the beginning of this century [21,22]. For instance, some of the well-known drugs bearing triazole moiety are presented in Figure 1, including **A** (Cefatrizine) and **B** (Tazobactum) as *β*-lactam antibiotic [1,23,24,25,26,27], **C** as anti-cancer reagent [23,28], **D** as potential nonpeptidic angiotensin (II) receptor antagonists [29,30], **E** as a toll-like receptor [29,31], **F** as a mental disorders medicine [32], and **G** as a wall teichoic acid active antibiotic [11].

**Scheme 1:**
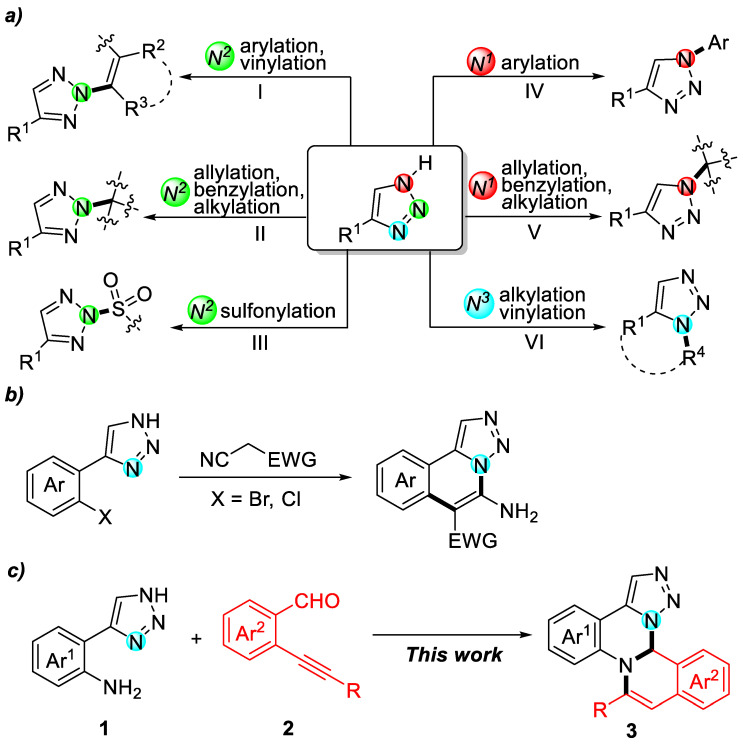
Modifications of the *NH*-1,2,3-triazoles *via C-N* bond formation. (**a**) One bond formation to substituted 1,2,3-triazoles; (**b**) Two bonds formation to tricyclic fused 1,2,3-triazoles; (**c**) Three bonds formation to pentacyclic fused 1,2,3-triazoles.

Owing to these pharmaceutical and biological properties, the constructions of various 1,2,3-triazoles are of paramount significance. The 4-monosubstitued 1,2,3-triazoles play remarkable roles in the triazole family [32,33,34,35,36,37,38,39,40,41]. Though the main access to this kind of compound, referring to the cycloaddition reaction of acetylene or its substitute with an azide source [42,43,44,45,46,47], could deliver respectable structures, the variations with the core are far from enough. So, direct modifications of the triazole ring, using its -*NH* moiety to expand diversity, attract broad attention. Over past decades, most work focused on one *C*-*N* bond formation process, especially on *N*^2^ of the heterocycle [32,33,34,35,39,48,49,50,51,52,53,54,55,56,57,58]. In early 2011, Buchwald demonstrated a Pd-catalyzed selective *C*-*N*^2^ coupling of 4-monosubstitued 1,2,3-triazoles with aryl bromides, delivering a series of arylated structures, 2,4-disubsituted 1,2,3-triazoles [33], which could not be obtained by traditional cycloaddition reactions. Later, Chen et al. reported a highly *N*^2^-selective *C-N* coupling using pyrrole or indole as an arylation reagent using *N*-iodophthalimide [34] or *N*-iodosuccinimide [48] as a mediate. Vinylation of the *N*^2^ was also explored by Shi et al. through Au-catalyzed alkyne activation with about 80% selectivity [49] (Scheme 1a(I)). Meanwhile, *N*^2^-selective allylation and benzylation were investigated, employing allenamide [50], aryldiazoacetate [32], and conjugate olefine (ketone) [51,52] as a reaction partner, respectively. Moreover, the *N*^2^-selective alkylation could be also achieved through an additional reaction of the *N(H)* group with (conjugate) alkene (ketone) [35,39,53,54] or by substitution with epoxide [55], dialkylamide [56], and alcohol [57] (Scheme 1a(II)). Additionally, Reddy et al. realized a highly regioselective *N*^2^-sulfonylation of 4-aryl-*NH*-1,2,3-triazoles with sodium sulfinate or thiosulfonate as a sulfonylating agent, mediated by I_2_ [58] (Scheme 1a(III)). 

Compared to the numerous *N*^2^-selective functionalizations, modifications on *N*^1^ and *N*^3^ of the 4-monosubstitued 1,2,3-triazoles are much more rare. In 2020, Ma group reported a Cu-catalyzed site- and enantio- selective ring opening of cyclic diaryliodoniums, delivering *N*^1^-arylation products of 1,4-disubstituted-1,2,3-triazoles [40] (Scheme 1a(IV)). Maddani et al. reached a selective *N*^1^-benzylation by 1,6-addition of the -*NH* to *para*-quinone methides mediated by acid of ClCH_2_CO_2_H [51]. Breit developed a rhodium-catalyzed asymmetric *N*^1^-selective allylation of triazole derivatives with internal alkynes and terminal allenes [36]. Very recently, Ji et al. reported a selective *N*^1^-alkylation of azoles through a three-component process involving ketones as alkylation reagents and *N*,*N*′-dimethylpropionamide as a carbon source [59] (Scheme 1a(V)). The *N*^3^-selective couplings of the 1,2,3-triazoles were demonstrated by Taylor and Li et al. with vinyl ketone (catalyzed by borinic acid) and alkyne (promoted by TBAF), delivering 1,5-disubstituted derivatives, respectively [60,61] (Scheme 1a(VI)).

In addition to the above one-bond-formation processes, cascade strategies to construct novel and diverse fused structures are more valued and important themes in organic synthesis. In 2013, Shi et al. [62] designed 4-(*ortho*-halo-aryl) 1,2,3-triazoles to merge with activated nitriles, forming a series of 5-amino-[1,2,3]triazolo-[5,1-*a*]isoquinoline derivatives, a kind of valued tricyclic fused 1,2,3-triazoles (Scheme 1b). Though some achievements were reached in this field, there is still a lack of strategies for constructing interesting and complex fused 1,2,3-triazole derivatives, which attracts us considerably as we are persistently interested in this area [63,64,65,66,67,68,69,70]. Sparked by the vigorous performance of 2-alkynylbenzaldehyde in the synthesis of fused cyclic compounds [71,72,73,74,75,76,77,78,79], herein, we designed 2-(1*H*-1,2,3-triazol-5-yl)anilines **1** to react with 2-alkynylbenzaldehydes **2** to construct isoquinolino [2,1-*a*] [1,2,3] triazolo [1,5-*c*] quinazolines **3** through a cascade process involving three *C*-*N* bond formations in one manipulation. This method features high efficiency, excellent atom economy, and only green by-products of H_2_O (Scheme 1c).

## 2. Results and Discussion

At the outset of our studies, the cascade reaction between 2-(1*H*-1,2,3-triazol-5-yl)aniline **1a** and 2-alkynylbenzaldehyde **2a** was investigated as a model (Table 1). To our delight, the reaction proceeded very successfully in the presence of 10 mol% AgNO_3_ at 80 °C for 1 hour using DMF as a solvent, delivering the product isoquinolino [2,1-*a*] [1,2,3] triazolo [1,5-*c*] quinazoline **3aa** with excellent yield (82%) (Entry 1). The structure of **3aa** was unambiguously confirmed by X-ray crystallography analysis (CCDC NO: 2133327) (see Supplementary Materials) [80]. The screening of solvents was then performed. Unfortunately, we found that other solvents, including toluene, DCE, MeCN, and DMSO, were less effective than DMF (Entries 2–5). Increasing or decreasing the temperature of the reaction could not lead to any further improvements in the yield (Entries 6–8). Changing the catalyst to AgOTf resulted in a slightly decreased yield, and the desired product **3aa** was obtained with 76% yield (Entry 9). However, the reaction proceeded very reluctantly in the presence of other catalysts (Ag_2_O, Ag_2_CO_3_, and AgOAc, without any target molecules detected (Entry 10–12)). When CuSCN or CuI is used instead of AgNO_3_, the yield drops sharply (Entry 13–14). However, the yield of the reaction slightly decreased when different amounts of AgNO_3_ catalyst were used (Entry 15–17). After testing different reaction concentrations, 0.2 M DMF was kept as the optimum one (Entry 18–19). Lastly, when the reaction time was extended to 2 hours, the target product was obtained in excellent 92% yield (Entry 20).

**Figure 2:**
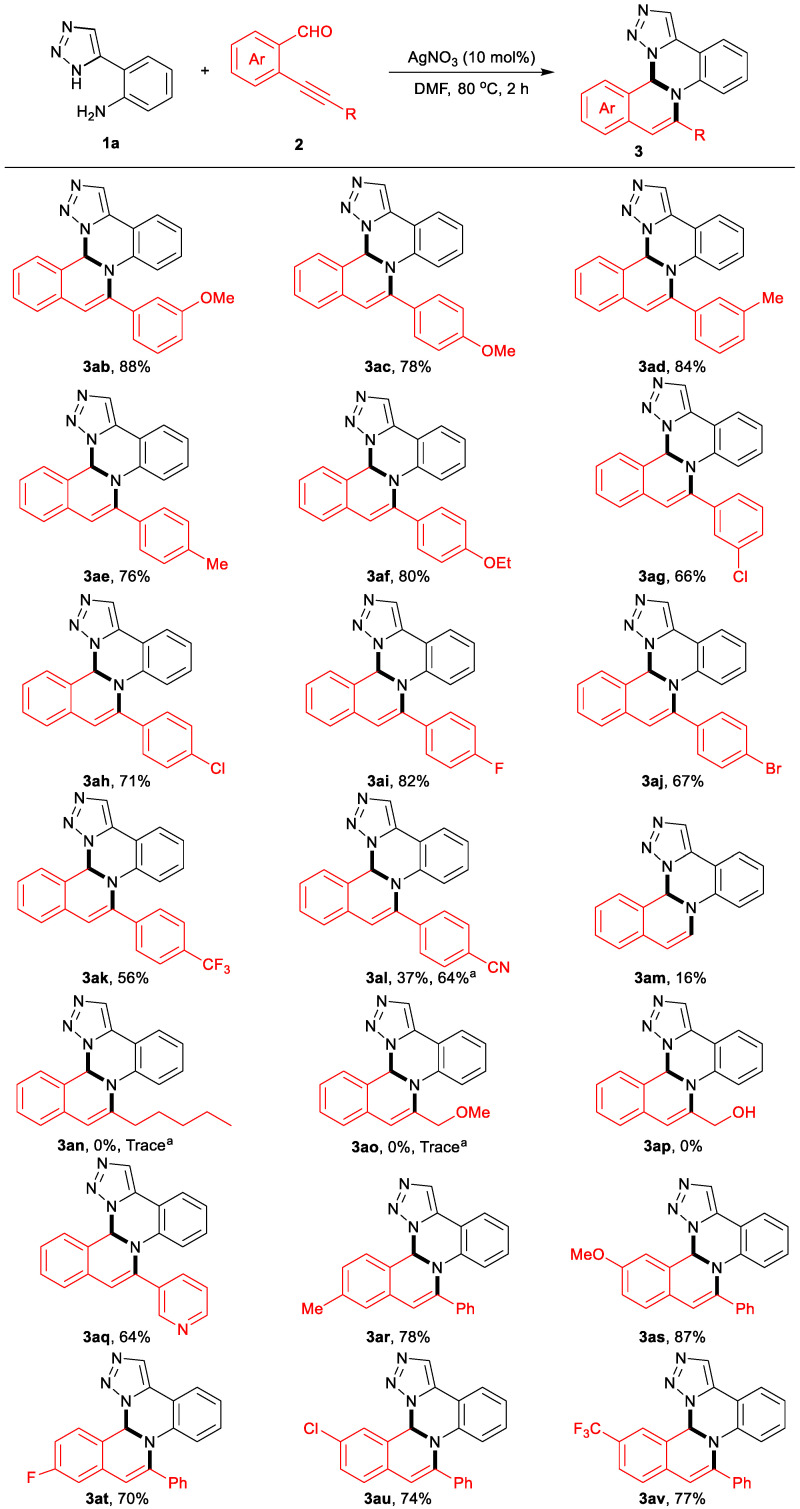
Scope of 2-alkynylbenzaldehyde.^.^ Reaction conditions: **1a** (0.2 mmol), **2** (0.2 mmol), AgNO_3_ (10 mol%) in DMF (1 mL) at 80 °C for 2 h. Isolated yield. ^a^ AgBF_4_ instead of AgNO_3_.

With the optimized reaction conditions in hand (Table 1, entry 18), the substrate scope of the cascade cyclization reaction was investigated with *o*-alkynyl aldehydes first. To our delight, a variety of *o*-alkynyl aldehydes with different alkynyl bearing substituted groups (including various aryl, alkyl, and heteroaryl) could work efficiently with 2-(1*H*-1,2,3-triazol-5-yl)aniline (**1a**), as shown in Figure 2. Reactions of alkynylbenzaldehydes containing electron-donating (**3ab**–**3af**) and electron-withdrawing (**3ag**–**3al**) groups on the phenyl ring proceeded smoothly to afford the corresponding products in moderate-to-good yields (37–88%). Generally, electron-donating groups substituted with alkynylbenzaldehyde (**3ab**–**3af**) were more successfully converted into target products than those with strong electron-withdrawing groups (**3ak**–**3al**). It should be noted that alkynylarylaldehyde with 2-pyridyl (**3aq**) was also suitable for this reaction, furnishing the corresponding products in satisfactory yield. Unfortunately, to substrates with aliphatic groups. such as pentyl, methoxymethyl, and hydroxymethyl on the 2-position of the alkynyl moiety (**3an**–**3ap**), the reaction could not provide the desired product. Surprisingly, when 2-((trimethylsilyl)ethynyl)benzaldehyde **2m** was used, the desilylation product (**3am**) was obtained in a low yield of 16%. Then, the effects of substituents on the core benzene ring linked directly to the formyl group were also studied. It was found that both electron-rich (–Me and –OMe) and -poor (–F, –Cl, and –CF_3_) groups were well tolerated in the reactions, and good yields were obtained (**3ar**–**3av**).

**Figure 3:**
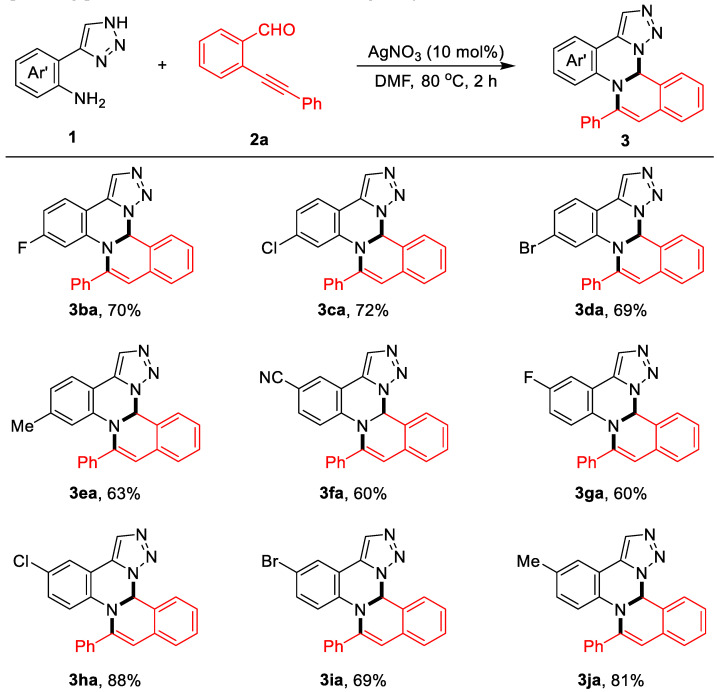
Scope of 2-(1*H*-1,2,3-triazol-5-yl)aniline. Reaction conditions: **1a** (0.2 mmol), **2** (0.2 mmol), AgNO_3_ (10 mol%) in DMF (1 mL) at 80 °C for 2 h. Isolated yield.

To gain further insight into the reaction, we continued our study by examining the 2-(1*H*-1,2,3-triazol-5-yl)aniline substrate scope, as shown in Figure 3. Gratifyingly, different electron-withdrawing group (–F, –Cl, –Br, –CN) and electron-donating group of -Me on 4- or 5-position of the phenyl ring (**3ba**–**3ja**) were perfectly tolerated, with the corresponding products obtained in moderate-to-good yields (60–88%).

**Scheme 2:**
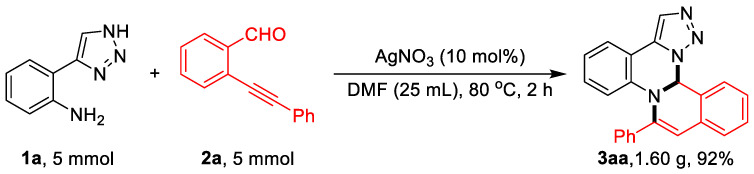
Gram scale experiment.

To illustrate the synthetic applicability of the protocol, the reaction was conducted on a gram-scale. A reaction of 5 mmol of **1a** and **2a** in 25 mL of DMF was carried out, and it could proceed smoothly under the optimized conditions to produce the product **3aa** in 92% (1.60 g) yield within 2 h (Scheme 2).

**Scheme 3:**
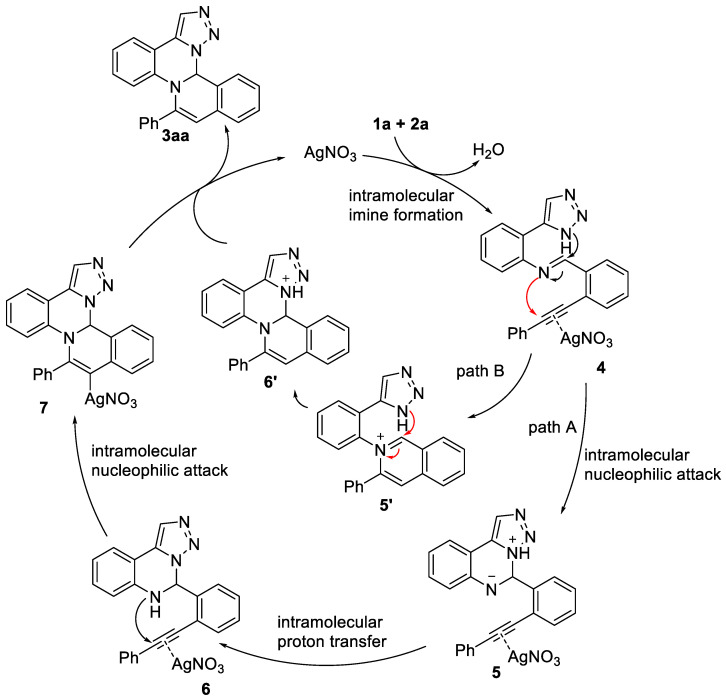
Proposed mechanism.

Based on our studies and previous reports [72,73,74,75], a plausible mechanism for the formation of target product **3aa** is presented in Scheme 3. The condensation reaction of 2-(1*H*-1,2,3-triazol-5-yl)aniline **1a** and 2-alkynylbenzaldehyde **2a** gives an imine in which the C≡C bond coordinates to AgNO_3_ catalyst to generate intermediate **4**. Then, two possibilities may exist for the formation of compound **3aa**. In path A, intermediate **4** would first undergo the intramolecular nucleophilic attack of the 1,2,3-triazole’s *N*^3^ atom onto the imine carbon center to form intermediate **5** (the first amination). Intramolecular proton transfer then occurred, producing fused tricyclic intermediate **6**, which would undergo a second intramolecular nucleophilic attack of the –*NH* group onto the triple bond, upon the π-activation by AgNO_3_, to afford **7** (the second (hydro-) amination), then deliver the desired compound **3aa** through protonolysis. Alternatively (path B), from the *N*-nucleophilic attack of the imine to the triple bond activated by AgNO_3_, imine cation intermediate **5’** could be formed initially, followed by intramolecular nucleophilic attack of triazole’s *N*^3^ to the carbon center of the formed imine to produce the fused pentacyclic intermediate **6’**, which would then give the final compound **3aa** through the subsequent deprotonation.

## 3. Materials and Methods

### 3.1. Synthesis of Various Substituted 2-(1H-1,2,3-Triazol-5-yl) Aniline (Take 1a as An Example) [81,82]

**Scheme 4:**
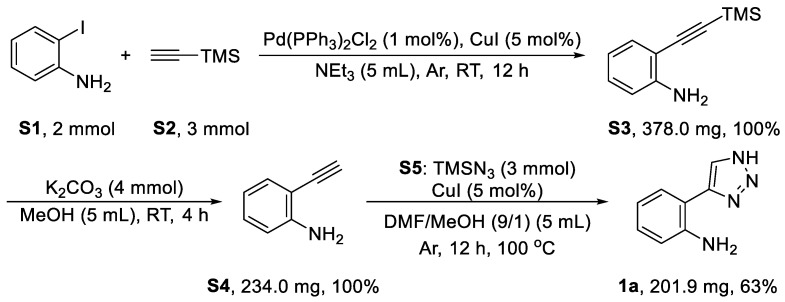
Synthesis of substrate **1a**.

A 15 mL flask equipped with a magnetic stir bar was charged with 2-iodoaniline **S1** (2 mmol), trimethylsilylacetylene **S2** (3 mmol), bis(triphenylphosphine)palladium (II) chloride (1 mol%), cuprous iodide (5 mol%), and 5 mL of triethylamine. The solution was stirred at room temperature under argon for 12 h. Upon completion of the reaction, the solvent was evaporated under vacuum, and the crude product was purified by column chromatography on silica gel (EtOAc:Petrol = 1:50), giving the pure product **S3** (Scheme 4).

A 15 mL flask equipped with a magnetic stir bar was charged with 2-((trimethylsilyl)ethynyl)aniline **S3** (2 mmol), potassium carbonate (4 mmol), and 5 mL of methanol. The solution was stirred at room temperature for 4 h. Upon completion of the reaction, the mixture was added to H_2_O (15 mL) and extracted with EtOAc (3 × 15 mL). The combined organic layer was washed with brine (3 × 5 mL), dried over Na_2_SO_4_, and concentrated under reduced pressure to afford product **S4** (Scheme 4).

A 15 mL flask equipped with a magnetic stir bar was charged with 2-ethynylaniline **S4** (2 mmol), TMSN_3_ **S5** (3 mmol), cuprous iodide (5 mol%), and 5 mL of mixed solvent (DMF/MeOH = 9/1). The solution was stirred at 100 °C under argon for 12 h. Upon completion of the reaction, the mixture was added to H_2_O (15 mL) and extracted with EtOAc (3 × 15 mL). The combined organic layer was washed with brine (3 × 5 mL), dried over Na_2_SO_4_, and concentrated under reduced pressure to afford a crude product. Purification by column chromatography on silica gel (EtOAc:Petrol = 1:3) afforded the pure product **1a** (Scheme 4).

### 3.2. Synthesis of Various Substituted 2-(Phenylethynyl)benzaldehyde (Take 2a as An Example) [83]

**Scheme 5:**
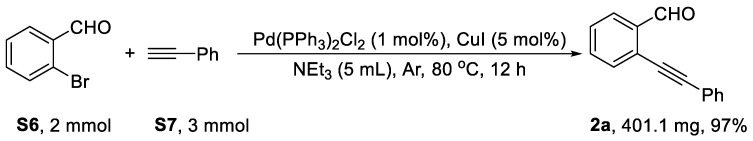
Synthesis of substrate **2a**.

A 15 mL flask equipped with a magnetic stir bar was charged with 2-bromobenzaldehyde **S6** (2 mmol), phenylacetylene **S7** (3 mmol), bis(triphenylphosphine)palladium (II) chloride (1 mol%), cuprous iodide (5 mol%), and 5 mL of triethylamine. The solution was stirred at 80 °C under argon for 12 h. Upon completion of the reaction, the solvent was evaporated under vacuum, and the crude product was purified by column chromatography on silica gel (Petrol), giving the pure product **2a** (Scheme 5).

### 3.3. General Procedure for Synthesis Pentacyclic Fused Triazoles (Take 3aa as An Example)

A 15 mL flask equipped with a magnetic stir bar was charged with 2-(1*H*-1,2,3-triazol-5-yl)aniline **1a** (0.2 mmol), 2-alkynylbenzaldehyde **2a** (0.2 mmol), and 1 mL of DMF. The solution was stirred at 80 °C under air for 2 h. Upon completion of the reaction, the mixture was added to H_2_O (15 mL) and extracted with EtOAc (3 × 15 mL). The combined organic layer was washed with brine (3 × 5 mL), dried over Na_2_SO_4_, and concentrated under reduced pressure to afford a crude product. Purification by column chromatography on silica gel (EtOAc:Petrol = 1:3) afforded the desired product **3aa**.

## 4. Conclusions

In summary, we developed a cascade process of condensation/in-situ generated imine and alkyne aminations of 2-(1*H*-1,2,3-triazol-5-yl)anilines with 2-alkynylbenzaldehydes catalyzed by AgNO_3_ to deliver novel isoquinoline and quinazoline-fused 1,2,3-triazoles in good-to-excellent yields. The methodology mainly features three new *C*-*N* bond formations in one convenient manipulation to construct various pentacyclic fused 1,2,3-triazoles, which may possess broad potential applications. Furthermore, the gram-scale reaction, broad substrate scope, excellent functional-group compatibility, and H_2_O as the only by-product, further demonstrate the atomic economy of this method.

## Figures and Tables

**Table 1:** Optimization of the reaction conditions ^a^.

Entry	Sol. (x mL)	Temp. (°C)	Cat. (x mol%)	Yield (%) ^b^
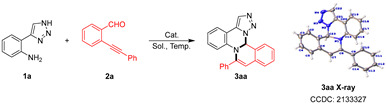				
1	DMF (2)	80	AgNO_3_ (10)	82
2	Toluene (2)	80	AgNO_3_ (10)	trace
3	DCE (2)	80	AgNO_3_ (10)	79
4	MeCN (2)	80	AgNO_3_ (10)	70
5	DMSO (2)	80	AgNO_3_ (10)	74
6	DMF (2)	60	AgNO_3_ (10)	74
7	DMF (2)	100	AgNO_3_ (10)	75
8	DMF (2)	120	AgNO_3_ (10)	70
9	DMF (2)	80	AgOTf (10)	76
10	DMF (2)	80	Ag_2_O (10)	ND
11	DMF (2)	80	Ag_2_CO_3_ (10)	ND
12	DMF (2)	80	AgOAc (10)	ND
13	DMF (2)	80	CuSCN (10)	22
14	DMF (2)	80	CuI (10)	30
15	DMF (2)	80	AgNO_3_ (5)	72
16	DMF (2)	80	AgNO_3_ (20)	79
17	DMF (2)	80	AgNO_3_ (30)	80
18	DMF (1)	80	AgNO_3_ (10)	83
19	DMF (4)	80	AgNO_3_ (10)	77
20 ^c^	DMF (1)	80	AgNO_3_ (10)	92

^a^ Reaction conditions: **1a** (0.2 mmol), **2a** (0.2 mmol), 1 hour. ^b^ Isolated yield. ^c^ Reaction time, 2 h.

## Data Availability

The data are available on request from the corresponding authors.

## Supplementary Materials

The following supporting information can be downloaded at: https://www.mdpi.com/article/10.3390/molecules27217567/s1, characterization data, ^1^H NMR and ^13^C NMR of compounds **1a**–**1j**, **3aa**–**3av** and **3ba**–**3ja**, and crystallographic data of **3aa**. References [81,82,83] are cited in supplementary materials.

## Sample Availability

Samples of the compounds **3aa**–**3av** and **3ba**–**3ja** are available from the authors.
