# Supplementary material for: Silver-Catalyzed Cascade Cyclization of Amino-NH-1,2,3-Triazoles with 2-Alkynylbenzaldehydes: An Access to Pentacyclic Fused Triazoles
Source: Molecules. 2022 Nov 4;27(21):7567. doi: 10.3390/molecules27217567 (PMC9655256; doi:10.3390/molecules27217567)
Supplement: Supplementary file 1 [file molecules-27-07567-s001.zip › molecules-1985750-supplementary.pdf]

Silver-Catalyzed Cascade Cyclization of Amino-*NH*-1,2,3-Triazoles with 2-Alkynylbenzaldehyde: An Access to Pentacyclic Fused Triazoles

Shuitao Zhang, Jianxin Li, Tiebo Xiao, Baomin Yang * and Yubo Jiang *

Faculty of Science, Kunming University of Science and Technology, Kunming 650500, China; E-mail: yangbm4985@sina.com; ybjiang@kust.edu.cn

Supplementary Materials

Table of Contents

1. General..S2

2. Representative experimental procedures S3

3. Compound characterization data S5

4. References S16

5. ^{1}H NMR and ^{13}C NMR spectra...S17

6. Crystallographic date of **3aa**..S55

1. General

Unless otherwise noted, for chromatography, 300-400 mesh silica gel (Qingdao, China) was employed. ^{1}H NMR and ^{13}C NMR spectra were measured in $CDCl_3$ or DMSO-d_6 and recorded on Brucker ARX 600 or 500 spectrometer. Chemical shifts (δ) were given in ppm, referenced to the residual proton resonance of $CDCl_3$ (7.26) or DMSO-d_6 (2.50), to the carbon resonance of $CDCl_3$ (77.16) or DMSO-d_6 (39.52). Coupling constants (J) were given in Hertz (Hz). The term m, q, t, d, s referred to multiplet, quartet, triplet, doublet, singlet. Exact masses (HRMS) were recorded on a high resolution magnetic mass spectrometer using electrospray ionization (ESI) techniques. Materials obtained from commercial suppliers were used without further purification.

2. Representative experimental procedures

Synthesis of various substituted 2-(1H-1,2,3-triazol-5-yl) aniline (take ***1a*** *as an example) [1,2]*

To a 15 mL flask equipped with a magnetic stir bar was charged with 2-iodoaniline **S1** (2 mmol), trimethylsilylacetylene **S2** (3 mmol), bis(triphenylphosphine)palladium(II) chloride (1 mol%), cuprous iodide (5 mol%) and 5 mL of triethylamine. The solution was stirred at room temperature under argon for 12 h. Upon completion of the reaction, the solvent was evaporated under vacuum, the crude product was purified by column chromatography on silica gel (EtOAc:Petrol= 1:50), giving the pure product **S3** (Scheme 4 in main text).

To a 15 mL flask equipped with a magnetic stir bar was charged with 2-((trimethylsilyl)ethynyl)aniline **S3** (2 mmol) and potassium carbonate (4 mmol) and 5 mL of methanol. The solution was stirred at room temperature under air for 12 h. Upon completion of the reaction, the mixture was added to H_2O (15 mL) and extracted with EtOAc (3 × 15 mL). The combined organic layer was washed with brine (3 × 5 mL), dried over Na_2SO_4 and concentrated under reduced pressure to afford a crude product **S4** (Scheme 4 in main text).

To a 15 mL flask equipped with a magnetic stir bar was charged with 2-ethynylaniline **S4** (2 mmol), $TMSN_3$ **S5** (3 mmol), cuprous iodide (5 mol%) and 5 mL of mixed solvent (DMF/MeOH = 9/1). The solution was stirred at 100 °C under argon for 12 h. Upon completion of the reaction, the mixture was added to H_2O (15 mL) and extracted with EtOAc (3 × 15 mL). The combined organic layer was washed with brine (3 × 5 mL), dried over Na_2SO_4 and concentrated under reduced pressure to afford a crude product.

Purification by column chromatography on silica gel (EtOAc:Petrol= 1:3) afforded the pure product **1a** (Scheme 4 in main text).

*Synthesis of various substituted 2-(phenylethynyl)benzaldehyde (take **2a** as an example) [3]*

To a 15 mL flask equipped with a magnetic stir bar was charged with 2-bromobenzaldehyde **S6** (2 mmol), phenylacetylene **S7** (3 mmol), bis(triphenylphosphine)palladium (II) chloride (1 mol%), cuprous iodide (5 mol%) and 5 mL of triethylamine. The solution was stirred at 80 ^{o}C under argon for 12 h. Upon completion of the reaction, the solvent was evaporated under vacuum, the crude product was purfied by column chromatography on silica gel (Petrol), giving the pure product **2a** (Scheme 5 in main text).

Procedure for the synthesis of isoquinolino [2,1-a] [1,2,3] triazolo [1,5-c] quinazoline:

To a 15 mL flask equipped with a magnetic stir bar was charged with 2-(1*H*-1,2,3-triazol-5-yl)aniline **1a** (0.2 mmol), 2-alkynylbenzaldehyde **2a** (0.2 mmol), and 1 mL of DMF. The solution was stirred at 80 ^{o}C under air for 2 h. Upon completion of the reaction, the mixture was added to H_2O (15 mL) and extracted with EtOAc (3 × 15 mL). The combined organic layer was washed with brine (3 × 5 mL), dried over Na_2SO_4 and concentrated under reduced pressure to afford a crude product. Purification by column chromatography on silica gel (EtOAc:Petrol= 1:3) afforded the desired product **3aa** (Scheme 2 in main text).

3. Compound characterization data:

6-Phenyl-11b*H*-isoquinolino[2,1-*a*][1,2,3]triazolo[1,5-*c*]quinazoline (3aa):

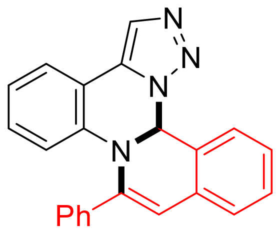

3aa

^{1}H NMR (600 MHz, $CDCl_3$) δ 8.14 (s, 1H), 7.78 (d, *J* = 7.2 Hz, 2H), 7.48 (dd, *J* = 7.8, 1.2 Hz, 1H), 7.41 – 7.35 (m, 3H), 7.33 (d, *J* = 7.8 Hz, 1H), 7.30 – 7.23 (m, 2H), 7.14 (t, *J* = 7.8 Hz, 1H), 6.99 (s, 1H), 6.88 (t, *J* = 7.8 Hz, 1H), 6.82 (t, *J* = 7.8 Hz, 1H), 6.57 (d, *J* = 7.8 Hz, 1H), 6.41 (d, *J* = 8.4 Hz, 1H); ^{13}C NMR (151 MHz, $CDCl_3$) δ 141.42, 137.80, 134.53, 131.66, 131.51, 130.66, 130.19, 129.41, 129.20, 129.01, 128.61, 127.96, 126.04, 125.40, 124.61, 122.74, 121.12, 118.08, 117.61, 112.66, 71.98; HRMS (ESI): calculated mass for $C_{23}H_{17}N_4^+$ [M+H$^+$]: 349.1448, mass found: 349.1451.

6-(3-Methoxyphenyl)-11b*H*-isoquinolino[2,1-*a*][1,2,3]triazolo[1,5-*c*]quinazoline (3ab):

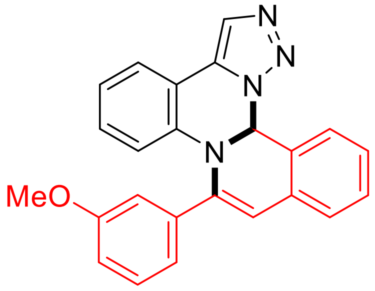

3ab

^{1}H NMR (600 MHz, $CDCl_3$) δ 8.13 (s, 1H), 7.48 (d, *J* = 7.8 Hz, 1H), 7.39 (d, *J* = 7.8 Hz, 1H), 7.36 – 7.27 (m, 4H), 7.24 (s, 1H), 7.18 – 7.12 (m, 1H), 6.98 (s, 1H), 6.92 (td, *J* = 7.8, 1.2 Hz, 2H), 6.83 (td, *J* = 7.2, 1.8 Hz, 1H), 6.56 (d, *J* = 7.6 Hz, 1H), 6.43 (d, *J* = 8.4 Hz, 1H), 3.80 (s, 3H); ^{13}C NMR (151 MHz, $CDCl_3$) δ 160.28, 141.43, 137.96, 136.28, 131.68, 131.63, 130.78, 130.35, 130.32, 129.09, 128.75, 128.04, 125.49, 124.66, 122.85, 121.23, 118.60, 118.13, 117.98, 114.86, 112.70, 111.68, 72.06, 55.45; HRMS (ESI): calculated mass for $C_{24}H_{19}N_4O^+$ [M+H$^+$]: 379.1554, mass found: 379.1556.

6-(4-Methoxyphenyl)-11b*H*-isoquinolino[2,1-*a*][1,2,3]triazolo[1,5-*c*]quinazoline (3ac):

^{1}H NMR (600 MHz, $CDCl_3$) δ 8.13 (s, 1H), 7.71 (d, *J* = 8.8 Hz, 2H), 7.49 (dd, *J* = 7.6, 1.3 Hz, 1H), 7.32 – 7.27 (m, 2H), 7.17 – 7.12 (m, 2H), 6.98 – 6.90 (m, 4H), 6.84 (td, *J* = 7.2, 0.8 Hz, 1H),

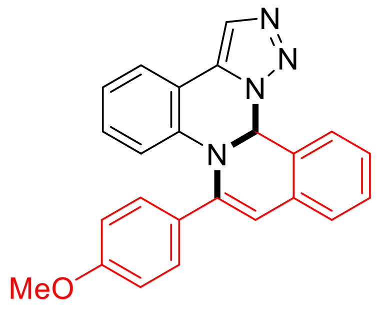

6.55 (d, J = 7.6 Hz, 1H), 6.42 (d, J = 8.4 Hz, 1H), 3.83 (s, 3H); ^{13}C NMR (151 MHz, $CDCl_3$) δ 160.69, 141.33, 138.06, 132.09, 131.65, 130.55, 130.27, 129.05, 128.26, 128.02, 127.61, 127.17, 125.20, 124.66, 122.80, 121.14, 118.27, 115.78, 114.66, 112.84, 72.09, 55.44; HRMS (ESI): calculated mass for $C_{24}H_{19}N_4O^+$ [M+H$^+$]: 379.1554, mass found: 379.1557.

6-(*m*-Tolyl)-11b*H*-isoquinolino[2,1-*a*][1,2,3]triazolo[1,5-*c*]quinazoline (3ad):

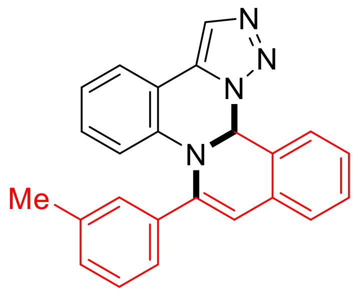

^{1}H NMR (600 MHz, $CDCl_3$) δ 8.14 (s, 1H), 7.62 (s, 1H), 7.59 (d, J = 7.8 Hz, 1H), 7.52 – 7.47 (m, 1H), 7.36 – 7.27 (m, 3H), 7.24 (s, 1H), 7.21 (d, J = 7.4 Hz, 1H), 7.16 (t, J = 7.2 Hz, 1H), 6.98 (s, 1H), 6.92 (t, J = 7.8 Hz, 1H), 6.84 (t, J = 7.2 Hz, 1H), 6.57 (d, J = 7.6 Hz, 1H), 6.44 (d, J = 8.4 Hz, 1H), 2.39 (s, 3H); ^{13}C NMR (151 MHz, $CDCl_3$) δ 141.76, 139.00, 138.06, 134.72, 131.85, 131.67, 130.78, 130.37, 130.33, 129.20, 129.09, 128.63, 128.03, 126.68, 125.43, 124.68, 123.41, 122.86, 121.15, 118.20, 117.63, 112.73, 72.11, 21.63; HRMS (ESI): calculated mass for $C_{24}H_{19}N_4^+$ [M+H$^+$]: 363.1604, mass found: 363.1606.

6-(*p*-Tolyl)-11b*H*-isoquinolino[2,1-*a*][1,2,3]triazolo[1,5-*c*]quinazoline (3ae):

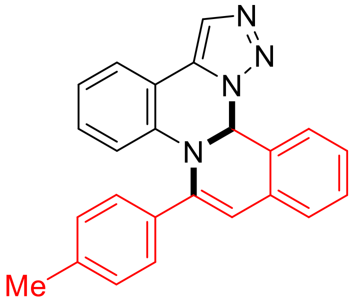

3ae

^{1}H NMR (600 MHz, $CDCl_3$) δ 8.13 (s, 1H), 7.68 (d, J = 8.2 Hz, 2H), 7.49 (dd, J = 7.6, 1.3 Hz, 1H), 7.33 –7.28 (m, 2H), 7.25 – 7.19 (m, 3H), 7.19 – 7.13 (m, 1H), 6.97 (s, 1H), 6.94 – 6.90 (m, 1H), 6.87 – 6.82 (m, 1H), 6.57 (d, J = 7.6 Hz, 1H), 6.42 (d, J = 7.8 Hz, 1H), 2.38 (s, 3H); ^{13}C NMR (151 MHz, $CDCl_3$) δ 141.55, 139.67, 137.99, 131.90, 131.82, 131.60, 130.67, 130.24, 129.99, 129.02, 128.44, 127.98, 126.04, 125.31, 124.62, 122.78, 121.09, 118.18, 116.76, 112.72, 72.04, 21.40; HRMS (ESI): calculated mass for $C_{24}H_{19}N_4^+$ [M+H$^+$]: 363.1604, mass found: 363.1605.

6-(4-Ethoxyphenyl)-11b*H*-isoquinolino[2,1-*a*][1,2,3]triazolo[1,5-*c*]quinazoline (3af):

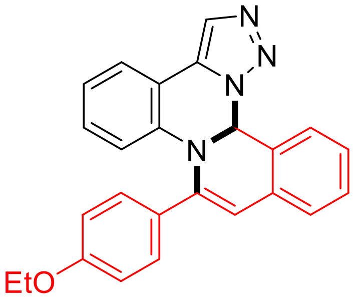

3af

^{1}H NMR (600 MHz, $CDCl_3$) δ 8.12 (s, 1H), 7.68 (d, J = 8.4 Hz, 2H), 7.47 (dd, J = 7.8, 1.2 Hz, 1H), 7.31 – 7.23 (m, 2H), 7.14 – 7.08 (m, 2H), 6.94 (s, 1H), 6.90 (t, J = 9.0 Hz, 3H), 6.82 (t, J = 7.8 Hz, 1H), 6.54 (d, J = 7.6 Hz, 1H), 6.42 (d, J = 8.4 Hz, 1H), 4.06 – 3.98 (m, 2H), 1.40 (t, J = 7.0 Hz, 3H); ^{13}C NMR (151 MHz, $CDCl_3$) δ 160.06, 141.32, 138.02, 132.07, 131.60, 130.48, 130.21, 128.99, 128.16, 127.95, 127.55, 126.91, 125.13, 124.60, 122.72, 121.07, 118.23, 115.61, 115.05, 112.76, 72.03, 63.57, 14.81; HRMS (ESI): calculated mass for $C_{25}H_{21}N_4O^+$ [M+H$^+$]: 393.1710, mass found: 393.1711.

6-(3-Chlorophenyl)-11b*H*-isoquinolino[2,1-*a*][1,2,3]triazolo[1,5-*c*]quinazoline (3ag):

^{1}H NMR (600 MHz, $CDCl_3$) δ 8.13 (s, 1H), 7.77 (s, 1H), 7.68 – 7.63 (m, 1H), 7.50 (dd, J = 7.6, 1.3 Hz, 1H), 7.36 – 7.29 (m, 4H), 7.26 (s, 1H), 7.19 (td, J = 7.8, 1.2 Hz, 1H), 6.98 (s, 1H), 6.97

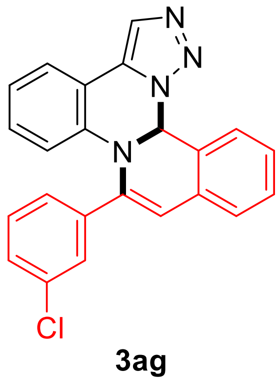

3ag

– 6.93 (m, 1H), 6.87 (td, *J* = 7.8, 1.2 Hz, 1H), 6.57 (d, *J* = 7.6 Hz, 1H), 6.37 (d, *J* = 8.2 Hz, 1H); ^{13}C NMR (151 MHz, $CDCl_3$) δ 140.23, 137.68, 136.73, 135.35, 131.55, 131.39, 130.85, 130.62, 130.40, 129.48, 129.23, 129.18, 128.15, 125.99, 125.72, 124.86, 124.36, 123.00, 121.54, 118.92, 118.01, 112.93, 72.04; HRMS (ESI): calculated mass for $C_{23}H_{16}N_4Cl^+$ [M+H$^+$]: 383.1058, mass found: 383.1061.

6-(4-Chlorophenyl)-11b*H*-isoquinolino[2,1-*a*][1,2,3]triazolo[1,5-*c*]quinazoline (3ah):

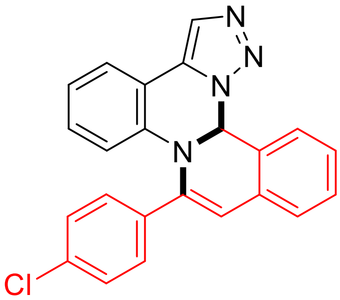

3ah

^{1}H NMR (600 MHz, $CDCl_3$) δ 8.14 (s, 1H), 7.71 (d, *J* = 8.6 Hz, 2H), 7.50 (dd, *J* = 7.6, 1.2 Hz, 1H), 7.38 (d, *J* = 8.6 Hz, 2H), 7.33 (d, *J* = 7.0 Hz, 1H), 7.30 (t, *J* = 7.4 Hz, 1H), 7.25 (s, 1H), 7.17 (t, *J* = 7.4 Hz, 1H), 6.97 (s, 1H), 6.95 – 6.91 (m, 1H), 6.86 (t, *J* = 7.4 Hz, 1H), 6.57 (d, *J* = 7.6 Hz, 1H), 6.35 (d, *J* = 8.4 Hz, 1H); ^{13}C NMR (151 MHz, $CDCl_3$) δ 140.33, 137.60, 135.21, 133.12, 131.47, 131.46, 130.65, 130.26, 129.46, 129.13, 128.91, 128.05, 127.32, 125.54, 124.76, 122.85, 121.40, 118.05, 117.97, 112.83, 71.96; HRMS (ESI): calculated mass for $C_{23}H_{16}N_4Cl^+$ [M+H$^+$]: 383.1058, mass found: 383.1060.

6-(4-Fluorophenyl)-11b*H*-isoquinolino[2,1-*a*][1,2,3]triazolo[1,5-*c*]quinazoline (3ai):

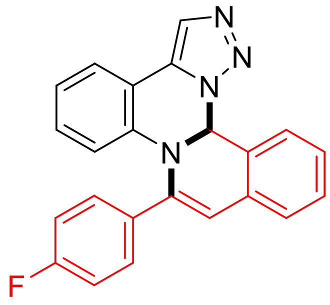

3ai

^{1}H NMR (600 MHz, $CDCl_3$) δ 8.14 (s, 1H), 7.81 – 7.73 (m, 2H), 7.50 (dd, *J* = 7.6, 1.4 Hz, 1H), 7.33 (d, *J* = 6.8 Hz, 1H), 7.30 (t, *J* = 7.4 Hz, 1H), 7.20 (s, 1H), 7.17 (t, *J* = 7.4 Hz, 1H), 7.10 (t, *J* = 8.6 Hz, 2H), 6.97 (s, 1H), 6.96 – 6.91 (m, 1H), 6.86 (t, *J* = 7.4 Hz, 1H), 6.57 (d, *J* = 7.6 Hz, 1H), 6.37 (d, *J* = 8.4 Hz, 1H); ^{13}C NMR (151 MHz, $CDCl_3$) δ 163.39 (d, *J* = 250.1 Hz), 140.50, 137.68, 131.56 (d, *J* = 16.3 Hz), 130.81 (d, *J* =

3.1 Hz), 130.56, 130.25, 129.10, 128.72, 128.04, 127.99, 127.93, 125.42, 124.75, 122.82, 121.35, 118.04, 117.44, 116.32 (d, *J* = 21.8 Hz), 112.86, 72.01; HRMS (ESI): calculated mass for $C_{23}H_{16}N_4F^+$ [M+H$^+$]: 367.1354, mass found: 367.1350.

6-(4-Bromophenyl)-11b*H*-isoquinolino[2,1-*a*][1,2,3]triazolo[1,5-*c*]quinazoline (3aj):

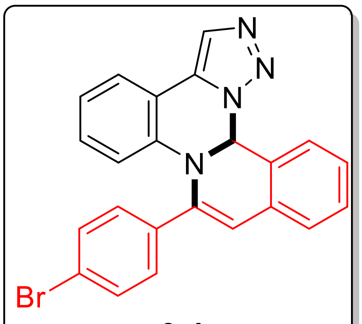

3aj

^{1}H NMR (600 MHz, $CDCl_3$) δ 8.14 (s, 1H), 7.64 (d, *J* = 8.6 Hz, 2H), 7.52 (d, *J* = 8.6 Hz, 2H), 7.50 (dd, *J* = 7.2, 1.2 Hz, 1H), 7.33 (d, *J* = 7.2 Hz, 1H), 7.29 (t, *J* = 7.4 Hz, 1H), 7.26 (s, 1H), 7.17 (td, *J* = 7.8, 1.2 Hz, 1H), 6.97 (s, 1H), 6.95 – 6.90 (m, 1H), 6.86 (td, *J* = 7.8, 1.2 Hz, 1H), 6.56 (d, *J* = 7.6 Hz, 1H), 6.35 (d, *J* = 8.2 Hz, 1H); ^{13}C NMR (151 MHz, $CDCl_3$) δ 140.35, 137.57, 133.56, 132.39, 131.45, 131.42, 130.66, 130.26, 129.12, 128.93, 128.04, 127.56, 125.54, 124.76, 123.51, 122.84, 121.40, 118.10, 117.94, 112.81, 71.93; HRMS (ESI): calculated mass for $C_{23}H_{16}N_4Br^+$ [M+H$^+$]: 427.0553, mass found: 429.0559.

6-(4-(Trifluoromethyl)phenyl)-11b*H*-isoquinolino[2,1-*a*][1,2,3]triazolo[1,5-*c*] quinazoline (3ak):

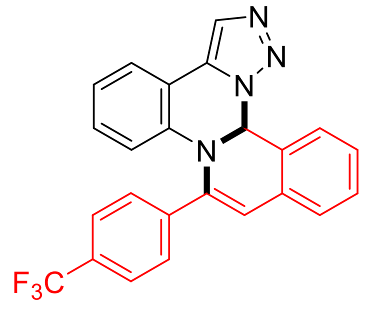

3ak

^{1}H NMR (600 MHz, $CDCl_3$) δ 8.15 (s, 1H), 7.90 (d, *J* = 8.2 Hz, 2H), 7.67 (d, *J* = 8.4 Hz, 2H), 7.52 (dd, *J* = 7.8, 1.8 Hz, 1H), 7.37 (d, *J* = 4.4 Hz, 2H), 7.32 (t, *J* = 7.8 Hz, 1H), 7.20 (t, *J* = 7.2 Hz, 1H), 7.00 (s, 1H), 6.96 – 6.91 (m, 1H), 6.88 (td, *J* = 7.8, 1.2 Hz, 1H), 6.59 (d, *J* = 7.6 Hz, 1H), 6.33 (d, *J* = 8.4 Hz, 1H); ^{13}C NMR (151 MHz, $CDCl_3$) δ 140.01, 138.11, 137.49, 131.49, 131.21, 130.85, 131.30 – 129.58 (m), 130.37, 129.38, 129.24, 128.14, 126.28, 126.24, 126.21, 125.87, 124.88, 122.96, 121.59, 119.75,

117.89, 112.90, 71.99; HRMS (ESI): calculated mass for $C_{24}H_{16}N_4F_3^+$ [M+H$^+$]: 417.1322, mass found: 417.1321.

4-(11b*H*-Isoquinolino[2,1-*a*][1,2,3]triazolo[1,5-*c*]quinazolin-6-yl)benzonitrile (3al):

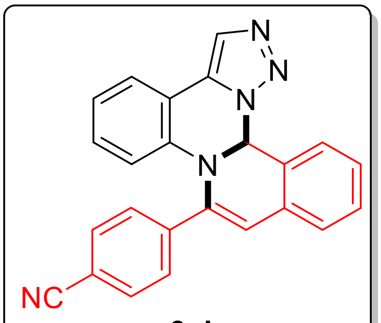

^{1}H NMR (600 MHz, CDCl$_3$) δ 8.14 (s, 1H), 7.88 (d, *J* = 8.4 Hz, 2H), 7.68 (d, *J* = 8.6 Hz, 2H), 7.52 (dd, *J* = 7.6, 1.2 Hz, 1H), 7.41 (s, 1H), 7.37 (d, *J* = 7.4 Hz, 1H), 7.31 (t, *J* = 7.2 Hz, 1H), 7.20 (t, *J* = 7.8 Hz, 1H), 6.98 (s, 1H), 6.97 – 6.91 (m, 1H), 6.88 (td, *J* = 7.8, 0.6 Hz, 1H), 6.57 (d, *J* = 7.6 Hz, 1H), 6.28 (d, *J* = 8.2 Hz, 1H); ^{13}C NMR (151 MHz, CDCl$_3$) δ 139.43, 138.91, 137.25, 132.98, 131.35, 130.93, 130.81, 130.31, 129.64, 129.26, 128.12, 126.41, 126.03, 124.93, 122.94, 121.71, 120.67, 118.55, 117.69, 112.89, 112.42, 71.84; HRMS (ESI): calculated mass for $C_{24}H_{16}N_5^+$ [M+H$^+$]: 374.1400, mass found: 374.1402.

11b*H*-Isoquinolino[2,1-*a*][1,2,3]triazolo[1,5-*c*]quinazoline (3am):

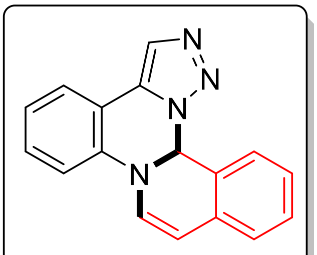

^{1}H NMR (600 MHz, CDCl$_3$) δ 7.96 (s, 1H), 7.65 – 7.59 (m, 2H), 7.47 – 7.38 (m, 2H), 7.37 – 7.28 (m, 3H), 7.15 (d, *J* = 7.6 Hz, 1H), 6.94 (s, 1H), 6.35 (d, *J* = 7.2 Hz, 1H), 5.65 (d, *J* = 7.2 Hz, 1H); ^{13}C NMR (151 MHz, CDCl$_3$) δ 139.48, 131.40, 131.23, 131.00, 130.57, 130.51, 130.48, 128.47, 126.35, 125.94, 124.98, 120.90, 119.84, 119.2, 101.67, 71.09; HRMS (ESI): calculated mass for $C_{17}H_{13}N_4^+$ [M+H$^+$]: 273.1135, mass found: 273.1136.

6-(Pyridin-3-yl)-11b*H*-isoquinolino[2,1-*a*][1,2,3]triazolo[1,5-*c*]quinazoline (3aq):

^{1}H NMR (600 MHz, CDCl$_3$) δ 9.07 (d, *J* = 1.8 Hz, 1H), 8.60 (d, *J* = 4.6 Hz, 1H), 8.12 (s, 1H), 8.05 – 7.93 (m, 1H), 7.50 (d, *J* = 7.4 Hz, 1H), 7.37 – 7.28 (m, 4H), 7.19 (t, *J* = 7.8 Hz, 1H), 6.99

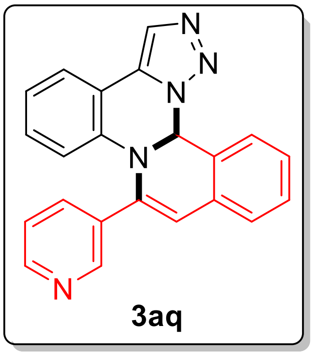

(s, 1H), 6.92 (t, J = 7.8 Hz, 1H), 6.86 (t, J = 7.2 Hz, 1H), 6.57 (d, J = 7.6 Hz, 1H), 6.34 (d, J = 8.4 Hz, 1H); ^{13}C NMR (151 MHz, $CDCl_3$) δ 150.16, 147.32, 138.57, 137.27, 133.56, 131.40, 131.10, 130.73, 130.47, 130.33, 129.27, 129.20, 128.10, 125.71, 124.90, 123.94, 122.91, 121.62, 119.20, 117.81, 112.93, 71.91; HRMS (ESI): calculated mass for $C_{22}H_{16}N_5^+$ [M+H$^+$]: 350.1400, mass found: 350.1401.

9-Methyl-6-phenyl-11b*H*-isoquinolino[2,1-*a*][1,2,3]triazolo[1,5-*c*]quinazoline (3ar)

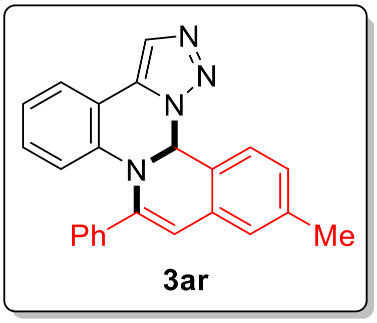

^{1}H NMR (600 MHz, $CDCl_3$) δ 8.13 (s, 1H), 7.83 – 7.73 (m, 2H), 7.49 (dd, J = 7.6, 1.2 Hz, 1H), 7.43 –7.37 (m, 3H), 7.22 (s, 1H), 7.15 (s, 1H), 6.98 (d, J = 2.4 Hz, 2H), 6.95 – 6.88 (m, 1H), 6.84 (td, J = 7.2, 0.6 Hz, 1H), 6.47 (d, J = 7.8 Hz, 1H), 6.41 (d, J = 8.4 Hz, 1H), 2.31 (s, 3H); ^{13}C NMR (151 MHz, CDCl3) δ 141.47, 138.95, 138.05, 134.76, 131.67, 131.57, 130.27, 129.40, 129.35, 129.27, 128.15, 127.99, 126.18, 126.06, 124.66, 122.77, 121.13, 118.09, 117.86, 112.78, 72.09, 21.29; HRMS (ESI): calculated mass for $C_{24}H_{19}N_4^+$ [M+H$^+$]: 363.1604, mass found: 363.1607.

10-Methoxy-6-phenyl-11b*H*-isoquinolino[2,1-*a*][1,2,3]triazolo[1,5-*c*]quinazoline (3as):

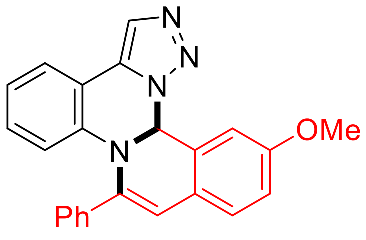

3as

^{1}H NMR (600 MHz, $CDCl_3$) δ 8.13 (s, 1H), 7.80 – 7.73 (m, 2H), 7.51 (dd, *J* = 7.2, 1.2 Hz, 1H), 7.41 (t, *J* = 7.4 Hz, 2H), 7.38 – 7.34 (m, 1H), 7.27 (d, *J* = 2.4 Hz, 1H), 7.24 (s, 1H), 6.99 (s, 1H), 6.97 – 6.93 (m, 1H), 6.88 – 6.82 (m, 2H), 6.43 (d, *J* = 8.0 Hz, 1H), 6.13 (d, *J* = 2.2 Hz, 1H), 3.66 (s, 3H); ^{13}C NMR (151 MHz, $CDCl_3$) δ 160.54, 139.36, 138.30, 134.85, 132.62, 131.70, 130.37, 129.26, 129.08, 128.05, 127.08, 125.79, 124.77, 124.73, 121.16, 118.05, 117.56, 114.44, 112.77, 108.85, 72.10, 55.45; HRMS (ESI): calculated mass for $C_{24}H_{19}N_4O^+$ [M+H$^+$]: 379.1554, mass found: 379.1556.

9-Fluoro-6-phenyl-11b*H*-isoquinolino[2,1-*a*][1,2,3]triazolo[1,5-*c*]quinazoline (3at):

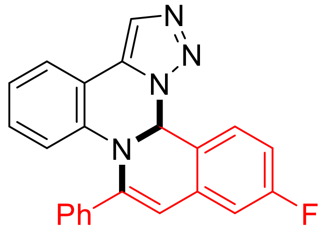

3at

^{1}H NMR (600 MHz, $CDCl_3$) δ 8.14 (s, 1H), 7.83 – 7.73 (m, 2H), 7.51 (dd, *J* = 7.2, 1.2 Hz, 1H), 7.46 – 7.37 (m, 3H), 7.19 (s, 1H), 7.04 (dd, *J* = 9.0, 2.4 Hz, 1H), 6.97 (s, 1H), 6.95 – 6.91 (m, 1H), 6.90 – 6.81 (m, 2H), 6.53 (dd, *J* = 7.8, 5.2 Hz, 1H), 6.39 (d, *J* = 7.8 Hz, 1H); ^{13}C NMR (151 MHz, $CDCl_3$) δ 163.14 (d, *J* = 246.9 Hz), 142.81, 137.57, 134.28, 133.71 (d, *J* = 8.9 Hz), 131.52, 130.37, 129.87, 129.35, 128.09, 126.46 (d, *J* = 2.5 Hz), 126.29, 124.76, 124.71, 121.45, 118.20, 116.75 (d, *J* = 2.0 Hz), 115.15 (d, *J* = 22.3 Hz), 112.73, 112.29 (d, *J* = 23.0 Hz), 71.71; HRMS (ESI): calculated mass for $C_{23}H_{16}N_4F^+$ [M+H$^+$]: 367.1354, mass found: 367.1352.

10-Chloro-6-phenyl-11b*H*-isoquinolino[2,1-*a*][1,2,3]triazolo[1,5-*c*]quinazoline (3au):

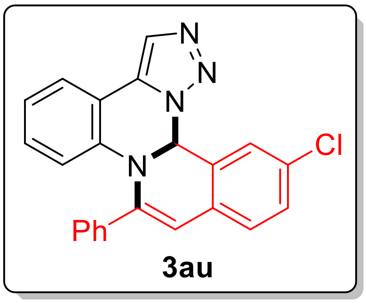

^{1}H NMR (600 MHz, $CDCl_3$) δ 8.15 (s, 1H), 7.83 – 7.72 (m, 2H), 7.52 (dd, *J* = 7.8, 1.8 Hz, 1H), 7.45 – 7.38 (m, 3H), 7.27 (s, 2H), 7.22 (s, 1H), 6.95 – 6.93 (m, 2H), 6.91 – 6.86 (m, 1H), 6.56 (s, 1H), 6.39 (d, *J* = 8.2 Hz, 1H); ^{13}C NMR (151 MHz, $CDCl_3$) δ 141.96, 137.60, 134.58, 134.38, 132.17, 131.60, 130.45, 130.32, 129.78, 129.39, 128.24, 126.70, 126.22, 124.89, 123.37, 121.57, 118.24, 116.69, 112.78, 71.61; HRMS (ESI): calculated mass for $C_{23}H_{16}N_4Cl^+$ [M+H$^+$]: 383.1058, mass found: 383.1061.

6-Phenyl-10-(trifluoromethyl)-11b*H*-isoquinolino[2,1-*a*][1,2,3]triazolo[1,5-*c*] quinazoline (3av):

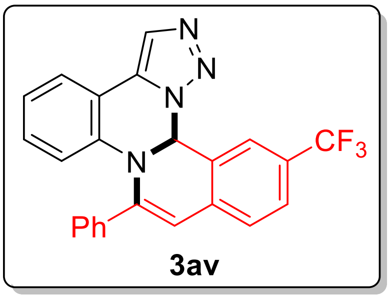

^{1}H NMR (600 MHz, $CDCl_3$) δ 8.17 (s, 1H), 7.82 – 7.77 (m, 2H), 7.57 (d, *J* = 7.8 Hz, 1H), 7.54 (dd, *J* = 7.2, 1.2 Hz, 1H), 7.48 – 7.41 (m, 4H), 7.27 (s, 1H), 7.00 (s, 1H), 6.96 – 6.92 (m, 1H), 6.92 – 6.87 (m, 1H), 6.82 (s, 1H), 6.40 (d, *J* = 8.2 Hz, 1H); ^{13}C NMR (151 MHz, $CDCl_3$) δ 143.76, 137.20, 135.11, 134.12, 131.54, 131.07, 130.46, 130.21, 129.46, 128.32, 126.50, 126.27 (d, *J* = 3.7 Hz), 125.68, 124.98, 123.73 (dd, *J* = 544.9, 272.5 Hz), 121.74, 120.00 (d, *J* = 3.7 Hz), 118.40, 116.31, 112.76, 71.60; HRMS (ESI): calculated mass for $C_{24}H_{16}N_4F_3^+$ [M+H$^+$]: 417.1322, mass found: 417.1323.

3-Fluoro-6-phenyl-11b*H*-isoquinolino[2,1-*a*][1,2,3]triazolo[1,5-*c*]quinazoline (3ba):

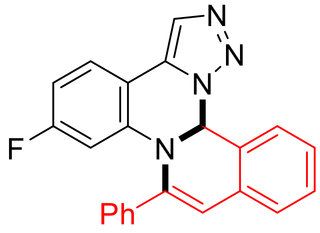

^{1}H NMR (600 MHz, $CDCl_3$) δ 8.10 (s, 1H), 7.81 – 7.74 (m, 2H), 7.48 – 7.38 (m, 4H), 7.38 – 7.31 (m, 2H), 7.28 (s, 1H), 7.19 (td, *J* = 7.2, 0.6 Hz, 1H), 7.00 (s, 1H), 6.61 – 6.51 (m, 2H), 6.13 (dd, *J* = 11.0, 2.4 Hz, 1H); ^{13}C NMR (151 MHz, $CDCl_3$) δ 163.66 (d, *J* = 248.6 Hz), 141.10, 139.95 (d, *J* = 10.6 Hz), 134.05, 131.55, 131.01, 130.76, 129.76, 129.43, 129.28, 128.89, 127.72, 126.29 (d, *J* = 10.2 Hz), 126.07, 125.68, 122.85, 118.15, 109.15 (d, *J* = 2.7 Hz), 108.52 (d, *J* = 22.9 Hz), 105.87 (d, *J* = 27.5 Hz), 71.90; HRMS (ESI): calculated mass for $C_{23}H_{16}N_4F^+$ [M+H$^+$]: 367.1354, mass found: 367.1353.

3-Chloro-6-phenyl-11b*H*-isoquinolino[2,1-*a*][1,2,3]triazolo[1,5-*c*]quinazoline (3ca):

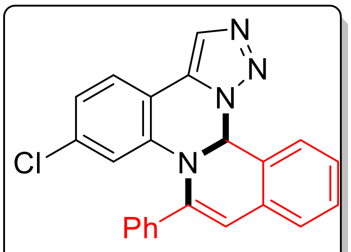

^{1}H NMR (600 MHz, $CDCl_3$) δ 8.12 (s, 1H), 7.82 – 7.74 (m, 2H), 7.46 – 7.38 (m, 4H), 7.38 – 7.31 (m, 2H), 7.28 (s, 1H), 7.19 (dd, *J* = 10.8, 4.0 Hz, 1H), 6.99 (s, 1H), 6.82 (dd, *J* = 8.2, 1.8 Hz, 1H), 6.56 (d, *J* = 7.6 Hz, 1H), 6.39 (d, *J* = 1.8 Hz, 1H); ^{13}C NMR (151 MHz, $CDCl_3$) δ 141.06, 139.12, 135.98, 134.01, 131.59, 130.90, 130.70, 129.81, 129.46, 129.34, 128.93, 128.15, 126.06, 125.75, 125.68, 122.84, 121.57, 118.30, 118.24, 111.43, 72.01; HRMS (ESI): calculated mass for $C_{23}H_{16}N_4Cl^+$ [M+H$^+$]: 383.1058, mass found: 383.1060.

3-Bromo-6-phenyl-11b*H*-isoquinolino[2,1-*a*][1,2,3]triazolo[1,5-*c*]quinazoline (3da):

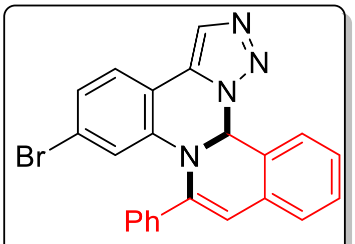

3da

^{1}H NMR (600 MHz, $CDCl_3$) δ 8.13 (s, 1H), 7.76 (d, *J* = 7.0 Hz, 2H), 7.46 – 7.38 (m, 3H), 7.38 – 7.31 (m, 3H), 7.27 (s, 1H), 7.19 (td, *J* = 7.2, 1.2 Hz, 1H), 7.02 – 6.94 (m, 2H), 6.55 (dd, *J* = 11.2, 4.6 Hz, 2H); ^{13}C NMR (151 MHz, $CDCl_3$) δ 141.00, 139.11, 133.96, 131.57, 130.92, 130.65, 129.80, 129.44, 129.33, 128.91, 128.18, 126.04, 125.80, 125.74, 124.43, 124.10, 122.81, 121.13, 118.23, 111.81, 72.01; HRMS (ESI): calculated mass for $C_{23}H_{16}N_4Br^+$ [M+H$^+$]: 427.0553, mass found: 429.0555.

3-Methyl-6-phenyl-11b*H*-isoquinolino[2,1-*a*][1,2,3]triazolo[1,5-*c*]quinazoline (3ea):

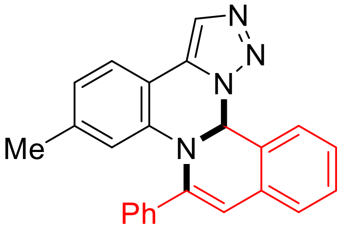

3ea

^{1}H NMR (600 MHz, $CDCl_3$) δ 8.09 (s, 1H), 7.79 (d, *J* = 7.2 Hz, 2H), 7.42 (t, *J* = 6.6 Hz, 2H), 7.40 –7.35 (m, 2H), 7.34 (d, *J* = 7.2 Hz, 1H), 7.30 (t, *J* = 7.4 Hz, 1H), 7.25 (s, 1H), 7.17 (t, *J* = 7.4 Hz, 1H), 6.96 (s, 1H), 6.66 (d, *J* = 7.2 Hz, 1H), 6.56 (d, *J* = 7.6 Hz, 1H), 6.21 (s, 1H), 1.99 (s, 3H); ^{13}C NMR (151 MHz, $CDCl_3$) δ 141.67, 140.67, 137.88, 134.74, 131.79, 131.74, 130.85, 129.42, 129.24, 129.03, 128.66, 127.59, 126.10, 125.43, 124.55, 122.86, 122.17, 118.75, 117.64, 110.19, 72.04, 21.86; HRMS (ESI): calculated mass for $C_{24}H_{19}N_4^+$ [M+H$^+$]: 363.1604, mass found: 363.1606.

6-Phenyl-11b*H*-isoquinolino[2,1-*a*][1,2,3]triazolo[1,5-*c*]quinazoline-2-carbonitrile (3fa):

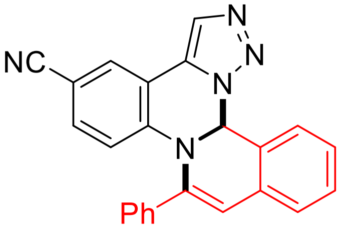

3fa

^{1}H NMR (600 MHz, $CDCl_3$) δ 8.22 (s, 1H), 7.79 (d, *J* = 1.8 Hz, 1H), 7.78 – 7.70 (m, 2H), 7.48 -7.41 (m, 3H), 7.41 – 7.34 (m, 2H), 7.32 (s, 1H), 7.23 (t, *J* = 7.2 Hz, 1H), 7.17 (dd, *J* = 8.4, 1.8 Hz, 1H), 7.08 (s, 1H), 6.59 (d, *J* = 7.6 Hz, 1H), 6.47 (d, *J* = 8.4 Hz, 1H); ^{13}C NMR (151 MHz, $CDCl_3$) δ 141.37, 140.42, 133.71, 133.62, 131.30, 130.80, 129.98,

129.87, 129.59, 129.19, 128.77, 128.40, 125.96, 122.73, 118.75, 118.55, 118.37, 113.38, 104.28, 72.05; HRMS (ESI): calculated mass for $C_{24}H_{16}N_5^+$ [M+H$^+$]: 374.1400, mass found: 374.1401.

2-Fluoro-6-phenyl-11b*H*-isoquinolino[2,1-*a*][1,2,3]triazolo[1,5-*c*]quinazoline (3ga):

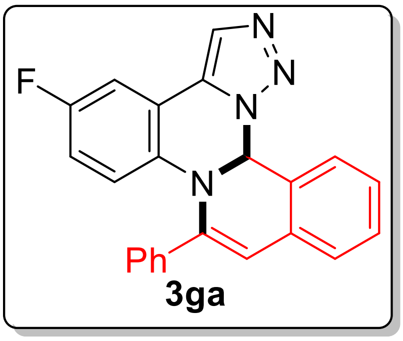

^{1}H NMR (600 MHz, $CDCl_3$) δ 8.13 (s, 1H), 7.83 – 7.73 (m, 2H), 7.45 – 7.38 (m, 3H), 7.37 – 7.30 (m, 2H), 7.27 (s, 1H), 7.22 – 7.17 (m, 2H), 6.97 (s, 1H), 6.62 (td, *J* = 9.0, 3.0 Hz, 1H), 6.55 (d, *J* = 7.6 Hz, 1H), 6.35 (dd, *J* = 9.0, 4.8 Hz, 1H); ^{13}C NMR (151 MHz, $CDCl_3$) δ 157.35 (d, *J* = 241.2 Hz), 141.50, 134.42, 134.13 (d, *J* = 2.2 Hz), 131.71, 130.97 (d, *J* = 2.7 Hz), 130.38, 129.67, 129.38, 129.24, 128.86, 128.52, 126.14, 125.57, 122.86, 119.52 (d, *J* = 7.9 Hz), 117.78, 117.05 (d, *J* = 22.6 Hz), 113.86 (d, *J* = 8.8 Hz), 111.31 (d, *J* = 24.7 Hz), 72.18; HRMS (ESI): calculated mass for $C_{23}H_{16}N_4F^+$ [M+H$^+$]: 367.1354, mass found: 367.1356.

2-Chloro-6-phenyl-11b*H*-isoquinolino[2,1-*a*][1,2,3]triazolo[1,5-*c*]quinazoline (3ha):

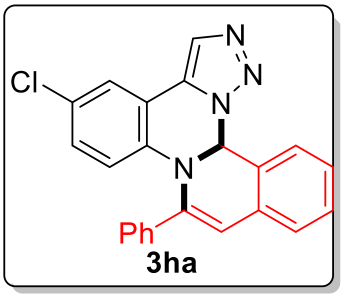

^{1}H NMR (500 MHz, $CDCl_3$) δ 8.13 (s, 1H), 7.76 (d, *J* = 6.8 Hz, 2H), 7.45 (d, *J* = 3.0 Hz, 1H), 7.44 – 7.36 (m, 3H), 7.36 – 7.28 (m, 2H), 7.27 (s, 1H), 7.17 (t, *J* = 7.4 Hz, 1H), 6.98 (s, 1H), 6.82 (d, *J* = 9.0 Hz, 1H), 6.55 (d, *J* = 7.6 Hz, 1H), 6.33 (d, *J* = 9.0 Hz, 1H); ^{13}C NMR (126 MHz, $CDCl_3$) δ 141.17, 136.44, 134.22, 131.59, 130.60, 130.50, 129.95, 129.63, 129.32, 129.22, 128.83, 128.35, 126.43, 126.05, 125.58, 124.28, 122.75, 119.40, 117.87, 114.16, 72.06; HRMS (ESI): calculated mass for $C_{23}H_{16}N_4Cl^+$ [M+H$^+$]: 383.1058, mass found: 383.1061.

2-Bromo-6-phenyl-11b*H*-isoquinolino[2,1-*a*][1,2,3]triazolo[1,5-*c*]quinazoline (3ia):

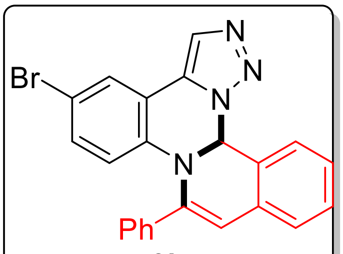

3ia

^{1}H NMR (600 MHz, $CDCl_3$) δ 8.13 (s, 1H), 7.81 – 7.72 (m, 2H), 7.59 (d, *J* = 2.2 Hz, 1H), 7.43 – 7.36 (m, 3H), 7.34 (d, *J* = 7.8 Hz, 1H), 7.30 (t, *J* = 7.4 Hz, 1H), 7.27 (s, 1H), 7.17 (td, *J* = 7.8, 0.6 Hz, 1H), 6.98 (s, 1H), 6.94 (dd, *J* = 9.0, 2.4 Hz, 1H), 6.55 (d, *J* = 7.6 Hz, 1H), 6.26 (d, *J* = 9.0 Hz, 1H); ^{13}C NMR (151 MHz, $CDCl_3$) δ 140.96, 136.81, 134.06, 132.74, 131.47, 130.41, 130.37, 129.59, 129.28, 129.19, 128.78, 128.35, 127.05, 125.96, 125.54, 122.68, 119.70, 117.83, 114.46, 113.55, 71.91; HRMS (ESI): calculated mass for $C_{23}H_{16}N_4Br^+$ [M+H$^+$]: 427.0553, mass found: 429.0556.

2-Methyl-6-phenyl-11b*H*-isoquinolino[2,1-*a*][1,2,3]triazolo[1,5-*c*]quinazoline (3ja):

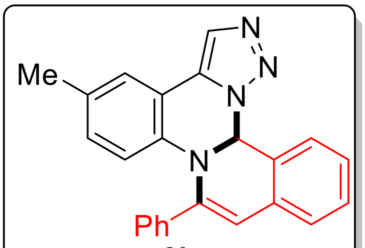

3ja

^{1}H NMR (600 MHz, $CDCl_3$) δ 8.14 (s, 1H), 7.84 – 7.73 (m, 2H), 7.44 – 7.36 (m, 3H), 7.35 – 7.31 (m, 2H), 7.29 (t, *J* = 7.4 Hz, 1H), 7.25 (s, 1H), 7.15 (t, *J* = 7.8 Hz, 1H), 6.97 (s, 1H), 6.70 (d, *J* = 8.4 Hz, 1H), 6.57 (d, *J* = 7.6 Hz, 1H), 6.31 (d, *J* = 8.4 Hz, 1H), 2.17 (s, 3H); ^{13}C NMR (151 MHz, $CDCl_3$) δ 141.53, 135.43, 134.64, 131.69, 131.61, 130.90, 130.60, 130.50, 129.33, 129.13, 128.92, 128.51, 127.82, 126.04, 125.30, 124.94, 122.70, 118.00, 117.27, 112.53, 71.99, 20.30; HRMS (ESI): calculated mass for $C_{24}H_{19}N_4^+$ [M+H$^+$]: 363.1604, mass found: 363.1605.

2-(1*H*-1,2,3-Triazol-5-yl)aniline (1a):

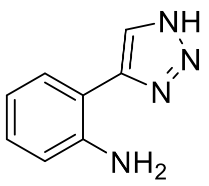

1a

^{1}H NMR (600 MHz, DMSO) δ 8.32 (s, 1H), 7.57 (d, *J* = 6.6 Hz, 1H), 7.05 (dd, *J* = 11.2, 4.0 Hz, 1H), 6.79 (d, *J* = 7.8 Hz, 1H), 6.60 (t, *J* = 7.4 Hz, 1H), 6.16 (s, 2H); ^{13}C NMR (151 MHz, DMSO) δ 146.69, 145.65, 128.77, 127.94, 115.89, 115.80, 112.45. HRMS (ESI): calculated mass for

$C_8H_9N_4^+$ [M+H$^+$]: 161.0822, mass found: 161.0825.

5-Fluoro-2-(1*H*-1,2,3-triazol-5-yl)aniline (1b):

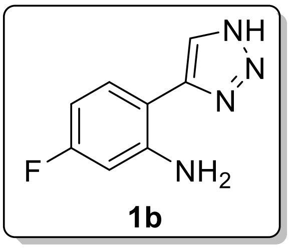

^{1}H NMR (600 MHz, DMSO) δ 8.29 (s, 1H), 7.59 (s, 1H), 6.58 (dd, *J* = 11.4, 2.4 Hz, 1H), 6.47 (s, 2H), 6.40 (td, *J* = 8.4, 3.0 Hz, 1H); ^{13}C NMR (151 MHz, DMSO) δ 162.81 (d, *J* = 241.5 Hz), 147.82, 147.74, 146.05, 129.80 (d, *J* = 10.1 Hz), 109.34, 102.57 (d, *J* = 22.1 Hz), 101.35 (d, *J* = 24.4 Hz).. HRMS (ESI): calculated mass for $C_8H_8N_4F^+$ [M+H$^+$]: 179.0728, mass found: 179.0727.

5-Chloro-2-(1*H*-1,2,3-triazol-5-yl)aniline (1c):

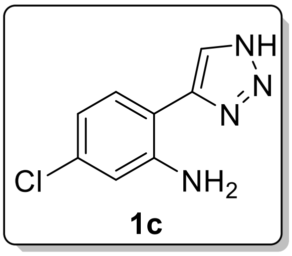

^{1}H NMR (600 MHz, DMSO) δ 8.34 (s, 1H), 7.58 (d, *J* = 7.8 Hz, 1H), 6.86 (d, *J* = 2.2 Hz, 1H), 6.61 (dd, *J* = 8.4, 2.4 Hz, 1H), 6.46 (s, 2H). ^{13}C NMR (151 MHz, DMSO) δ 147.08, 145.76, 133.06, 129.45, 115.38, 114.76, 111.47. HRMS (ESI): calculated mass for $C_8H_8N_4Cl^+$ [M+H$^+$]: 195.0432, mass found: 195.0436.

5-Bromo-2-(1*H*-1,2,3-triazol-5-yl)aniline (1d):

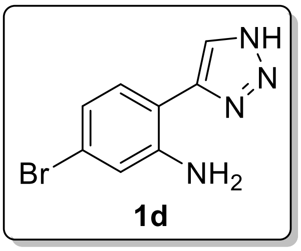

^{1}H NMR (500 MHz, DMSO) δ 8.33 (s, 1H), 7.52 (d, *J* = 6.4 Hz, 1H), 7.01 (d, *J* = 2.4 Hz, 1H), 6.74 (dd, *J* = 8.0, 1.5 Hz, 1H), 6.41 (s, 2H); ^{13}C NMR (126 MHz, DMSO) δ 147.15, 145.69, 131.25, 129.53, 118.07, 117.59, 111.90. HRMS (ESI): calculated mass for $C_8H_8N_4Br^+$ [M+H$^+$]: 238.9927, mass found: 238.9929.

5-Methyl-2-(1*H*-1,2,3-triazol-5-yl)aniline (1e):

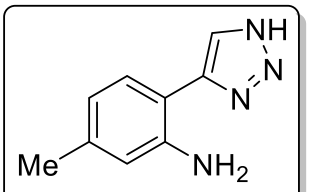

1e

^{1}H NMR (600 MHz, DMSO) δ 8.19 (s, 1H), 7.47 (s, 1H), 6.59 (s, 1H), 6.43 (d, *J* = 7.6 Hz, 1H), 6.06 (s, 2H), 2.19 (s, 3H); ^{13}C NMR (151 MHz, DMSO) δ 146.83, 145.52, 138.09, 130.82, 127.87, 116.91, 116.14, 109.81, 21.08. HRMS (ESI): calculated mass for $C_9H_{11}N_4^+$ [M+H$^+$]: 175.0978, mass found: 175.0976.

4-Amino-3-(1*H*-1,2,3-triazol-5-yl)benzonitrile (1f):

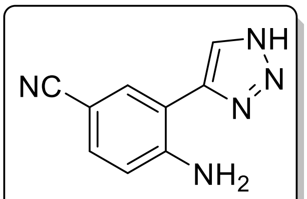

1f

^{1}H NMR (500 MHz, DMSO) δ 8.52 (s, 1H), 8.08 (s, 1H), 7.41 (dd, *J* = 8.5, 1.5 Hz, 1H), 7.13 (s, 2H), 6.89 (d, *J* = 8.6 Hz, 1H); ^{13}C NMR (126 MHz, DMSO) δ 149.43, 145.04, 132.23, 132.06, 120.28, 115.88, 112.62, 96.38. HRMS (ESI): calculated mass for $C_9H_8N_5^+$ [M+H$^+$]: 186.0774, mass found: 186.0772.

4-Fluoro-2-(1*H*-1,2,3-triazol-5-yl)aniline (1g):

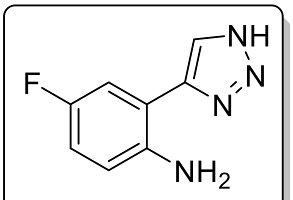

1g

^{1}H NMR (500 MHz, DMSO) δ 8.41 (s, 1H), 7.47 (dd, *J* = 10.2, 3.0 Hz, 1H), 6.92 (td, *J* = 8.6, 3.0 Hz, 1H), 6.80 (dd, *J* = 9.0, 5.0 Hz, 1H), 6.05 (s, 2H). ^{13}C NMR (126 MHz, DMSO) δ 154.09 (d, *J* = 230.1 Hz), 145.65, 142.28, 142.27, 117.05 (d, *J* = 7.6 Hz), 115.59 (d, *J* = 22.3 Hz), 113.50 (d, *J* = 23.2 Hz), 113.15 (d, *J* = 7.6 Hz). HRMS (ESI): calculated mass for $C_8H_8N_4F^+$ [M+H$^+$]: 179.0728, mass found: 179.0726.

4-Chloro-2-(1*H*-1,2,3-triazol-5-yl)aniline (1h):

^{1}H NMR (600 MHz, DMSO) δ 8.46 (s, 1H), 7.65 (s, 1H), 7.07 (dd, *J* = 9.0, 2.4 Hz, 1H), 6.81

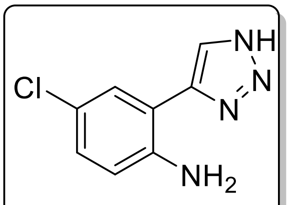

1h

(d, *J* = 9.0 Hz, 1H), 6.34 (s, 2H). ^{13}C NMR (151 MHz, DMSO) δ 145.57, 144.60, 128.33, 126.93, 119.04, 117.43, 113.88. HRMS (ESI): calculated mass for $C_8H_8N_4Cl^+$ [M+H$^+$]: 195.0432, mass found: 195.0429.

4-Bromo-2-(1*H*-1,2,3-triazol-5-yl)aniline (1i):

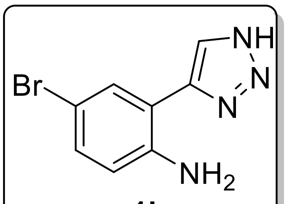

1i

^{1}H NMR (600 MHz, DMSO) δ 8.46 (s, 1H), 7.76 (s, 1H), 7.17 (dd, *J* = 9.0, 2.4 Hz, 1H), 6.77 (d, *J* = 9.0 Hz, 1H), 6.36 (s, 2H). ^{13}C NMR (151 MHz, DMSO) δ 145.43, 144.95, 131.07, 129.70, 117.87, 114.50, 106.34. HRMS (ESI): calculated mass for $C_8H_8N_4Br^+$ [M+H$^+$]: 238.9927, mass found: 238.9925.

4-Methyl-2-(1*H*-1,2,3-triazol-5-yl)aniline (1j):

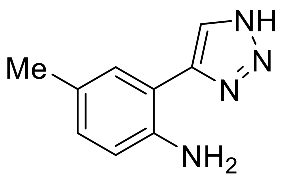

1j

^{1}H NMR (600 MHz, DMSO) δ 8.29 (s, 1H), 7.40 (s, 1H), 6.87 (d, *J* = 8.2 Hz, 1H), 6.71 (d, *J* = 8.2 Hz, 1H), 5.94 (s, 2H), 2.19 (s, 3H). ^{13}C NMR (151 MHz, DMSO) δ 146.76, 143.32, 129.56, 128.07, 124.21, 116.16, 112.49, 20.17. HRMS (ESI): calculated mass for $C_9H_{11}N_4^+$ [M+H$^+$]: 175.0978, mass found: 175.0980.

4. References

1. Li, H.-H.; Ye, S.-H.; Chen, Y.-B.; Luo, W.-F.; Qian, P.-C.; Ye, L.-W. Efficient and divergent synthesis of medium-sized lactams through zinc-catalyzed oxidative cyclization of indoly ynamides. *Chin. J. Chem*. **2020**, *38*, 263–268.
2. Röhrig, U.F.; Majjigapu, S.R.; Grosdidier, A.; Bron, S.; Stroobant, V.; Pilotte, L.; Colau, D.; Vogel, P.; den Eynde, B.J.V.; Zoete, V.; Michielin, O. Rational design of 4-aryl-1,2,3-triazoles for indoleamine 2,3-dioxygenase **1** inhibition. *J. Med. Chem*. **2012**, *55*, 5270–5290.
3. Dai, G.-X.; Larock, R.C. Synthesis of 3,4-disubstituted isoquinolines *via* palladium-catalyzed cross-coupling of *o*-(1-alkynyl)benzaldimines and organic halides. *Org. Lett*. **2001**, *3*, 4035–4038.

5. ^{1}H NMR and ^{13}C NMR spectra

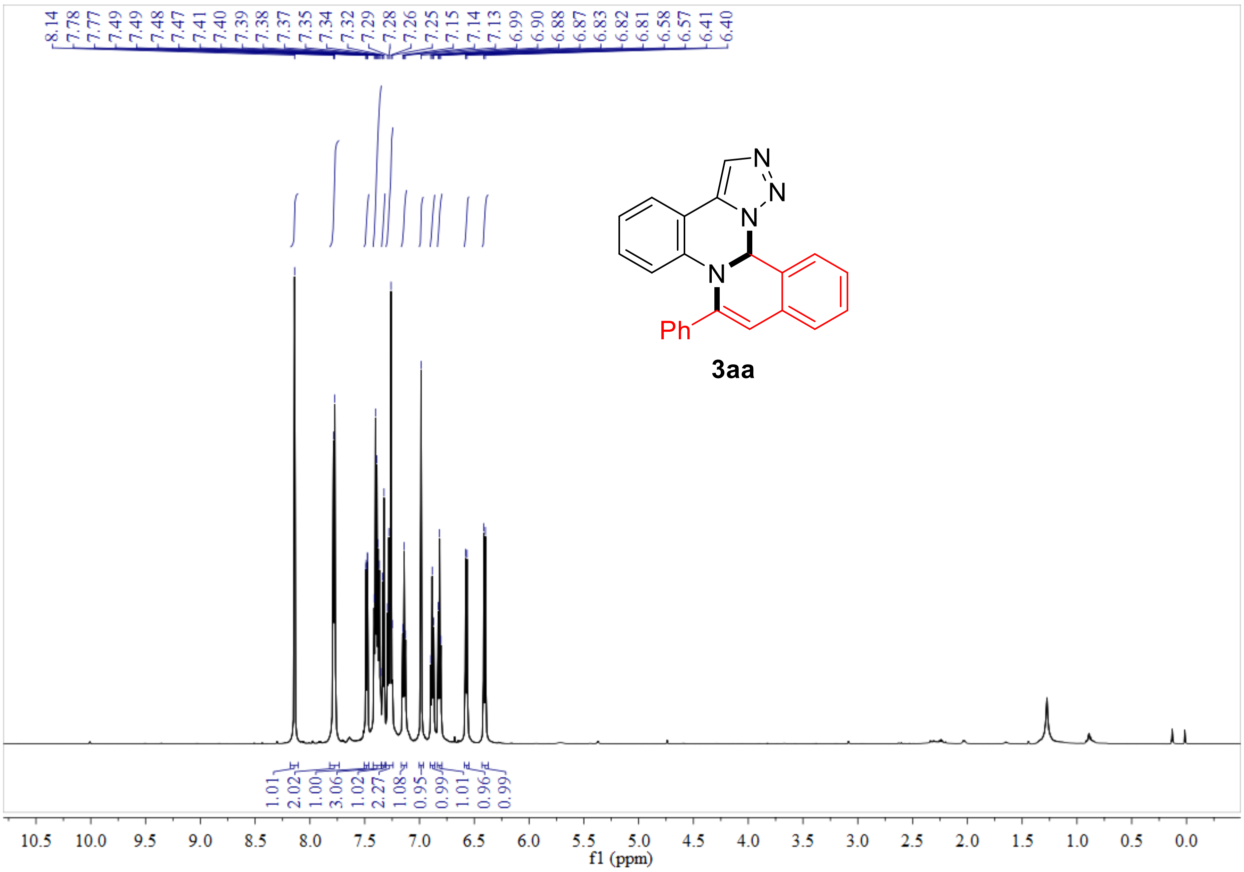

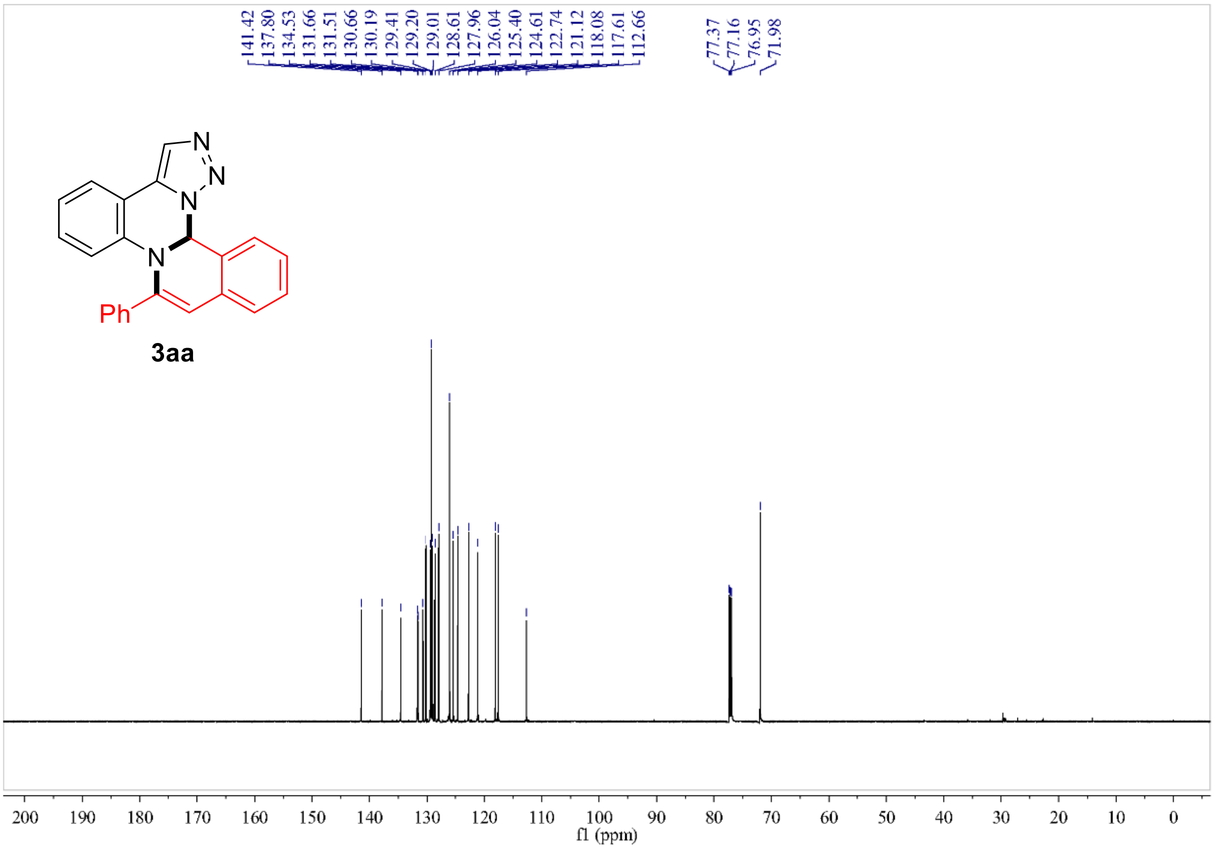

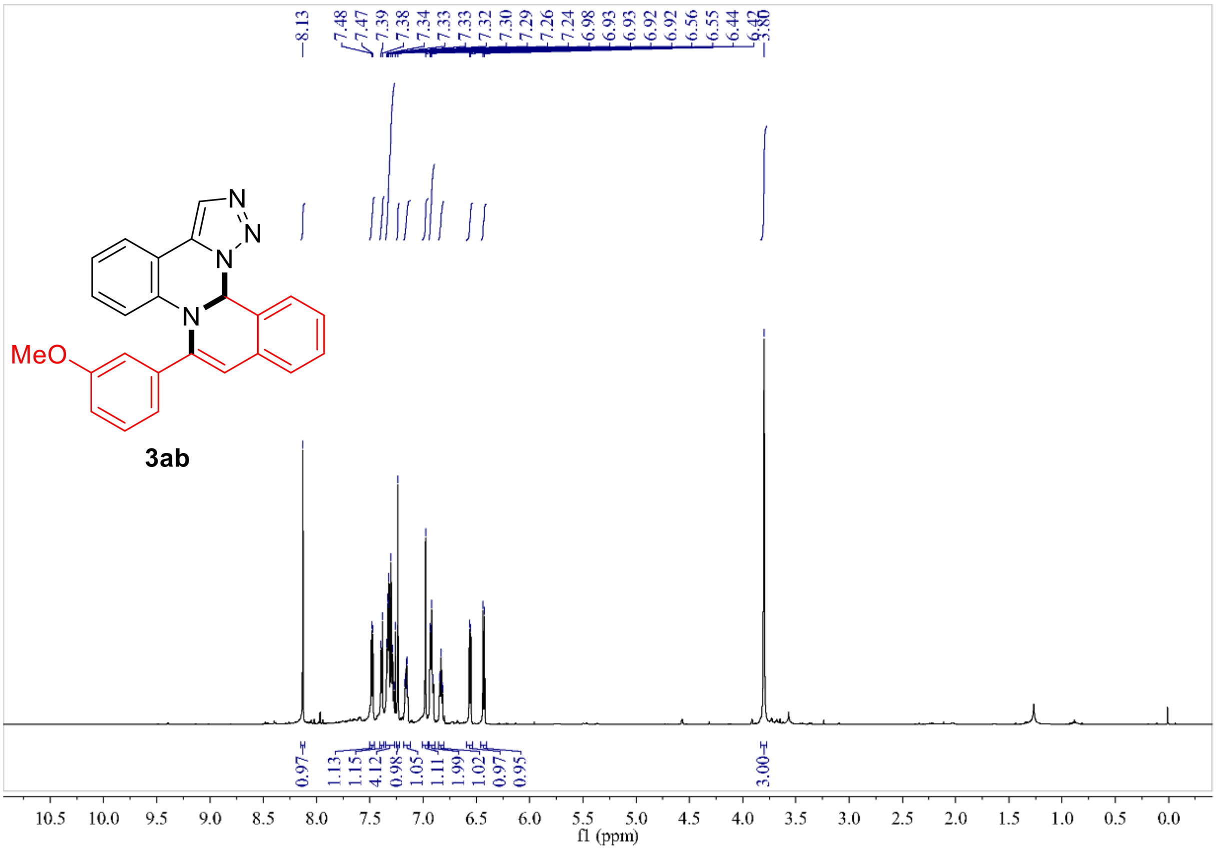

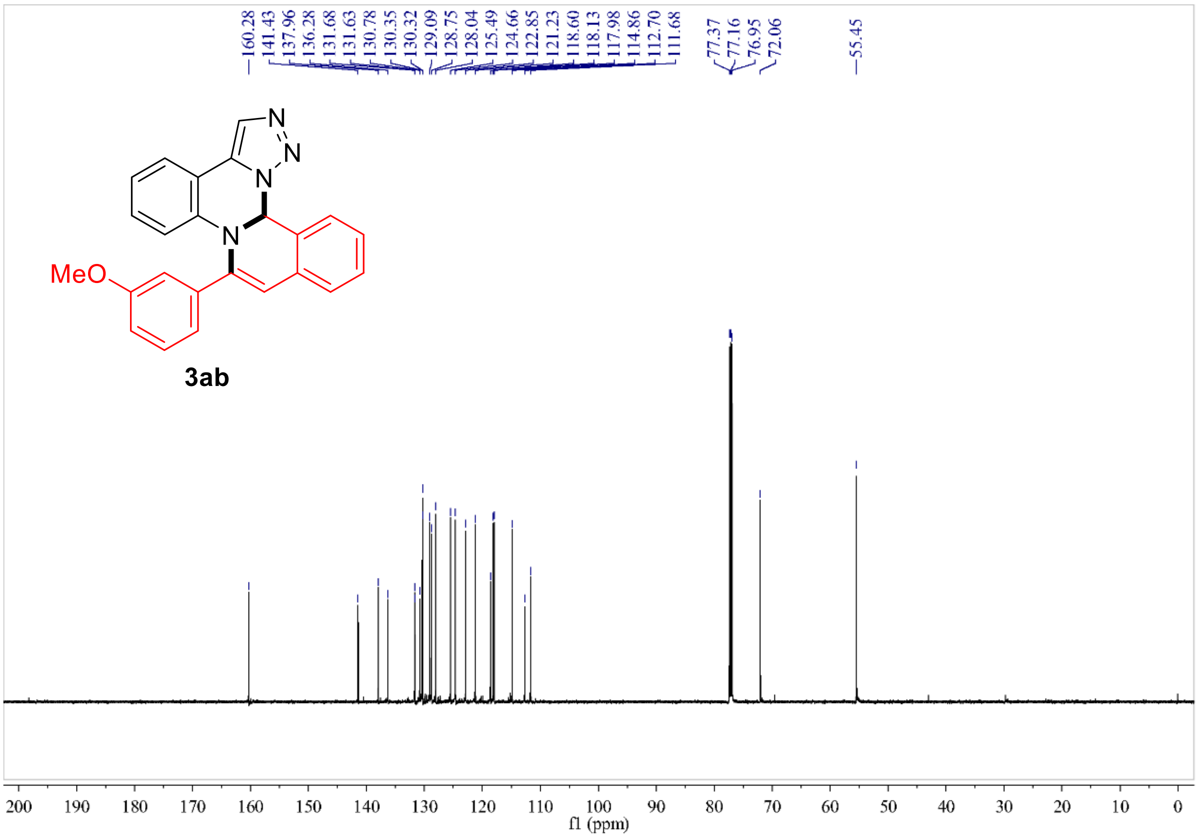

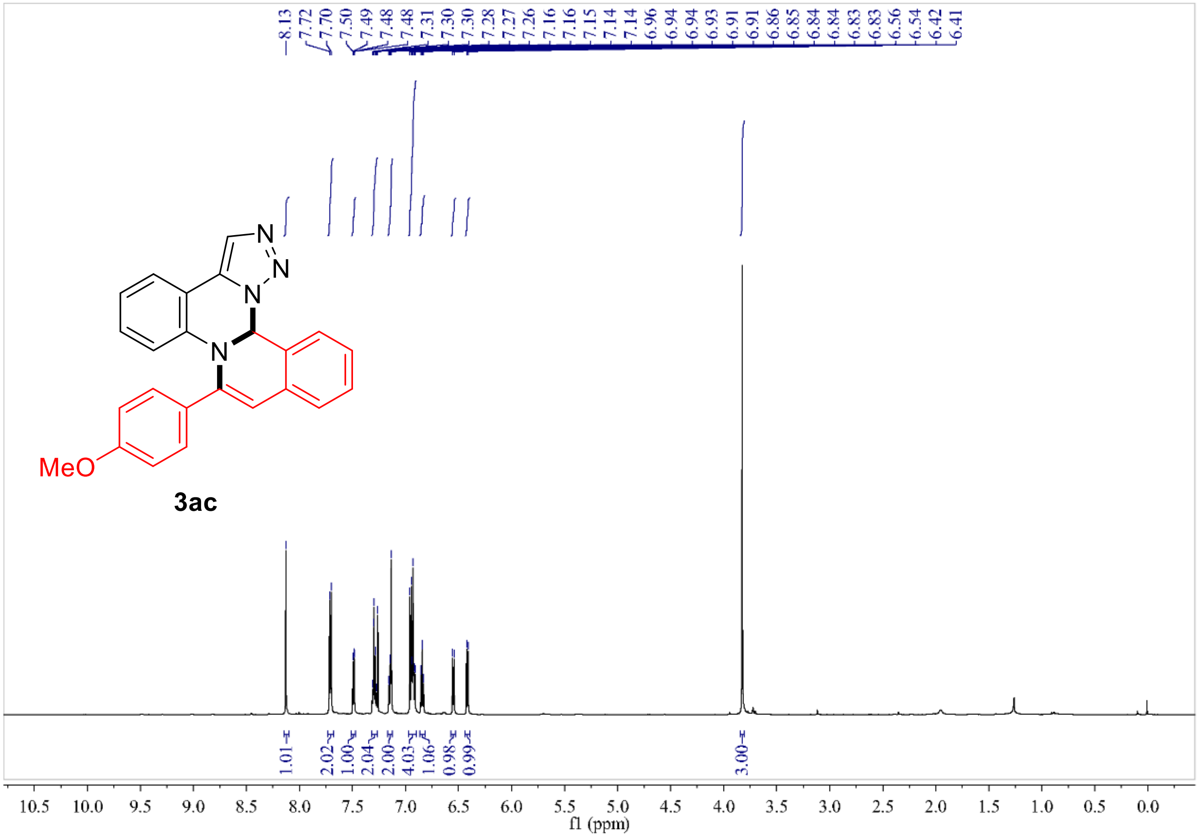
8.13
7.72
7.70
7.50
7.49
7.48
7.48
7.31
7.30
7.30
7.28
7.27
7.26
7.16
7.16
7.15
7.14
7.14
6.96
6.94
6.93
6.91
6.91
6.86
6.85
6.84
6.84
6.83
6.83
6.56
6.54
6.42
6.41
N
N
N
N
MeO
3ac
1.01
2.02
1.00
2.04
2.00
4.03
1.06
0.98
0.99
3.00
10.5
10.0
9.5
9.0
8.5
8.0
7.5
7.0
6.5
6.0
5.5
5.0
4.5
4.0
3.5
3.0
2.5
2.0
1.5
1.0
0.5
0.0
f1 (ppm)

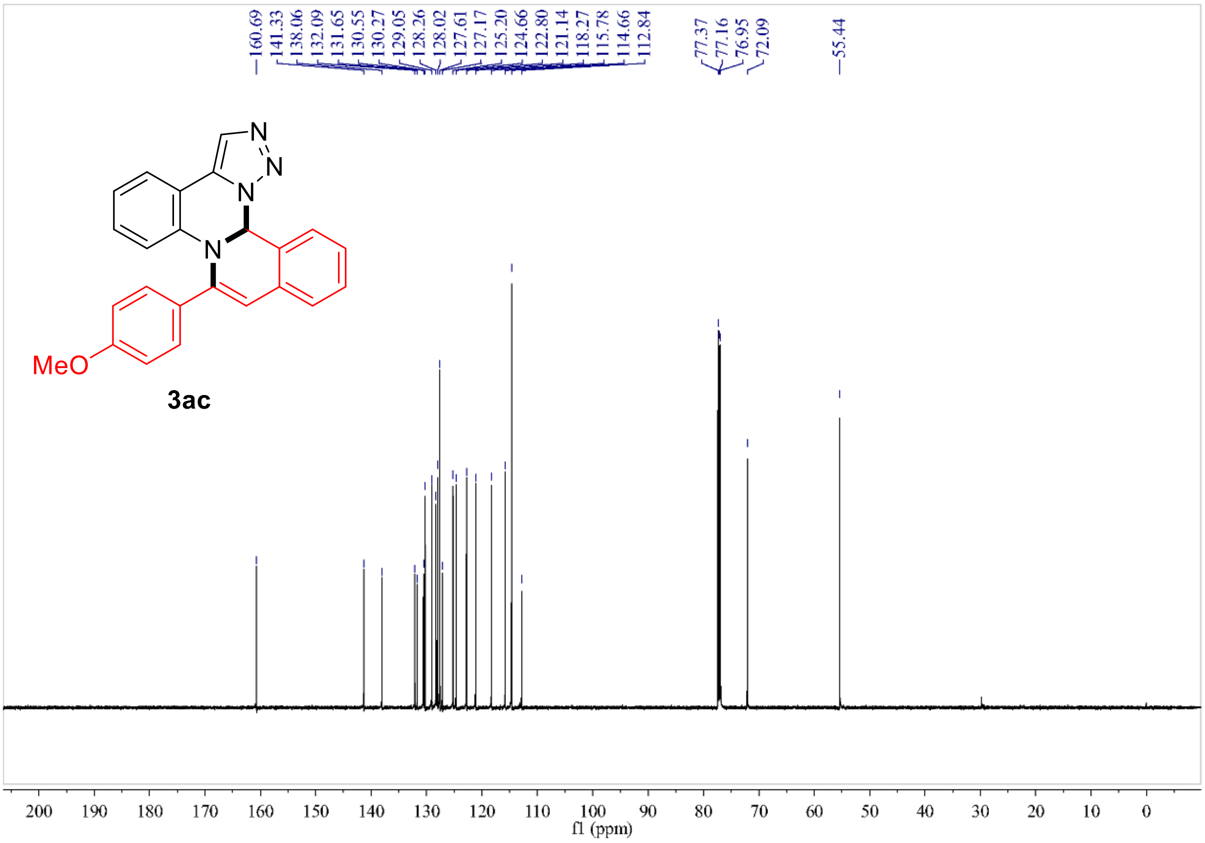
160.69
141.33
138.06
132.09
131.65
130.55
130.27
129.05
128.26
128.02
127.61
127.17
125.20
124.66
122.80
121.14
118.27
115.78
114.66
112.84
77.37
77.16
76.95
72.09
55.44
N
N
N
N
MeO
3ac
200
190
180
170
160
150
140
130
120
110
100
90
80
70
60
50
40
30
20
10
0
f1 (ppm)

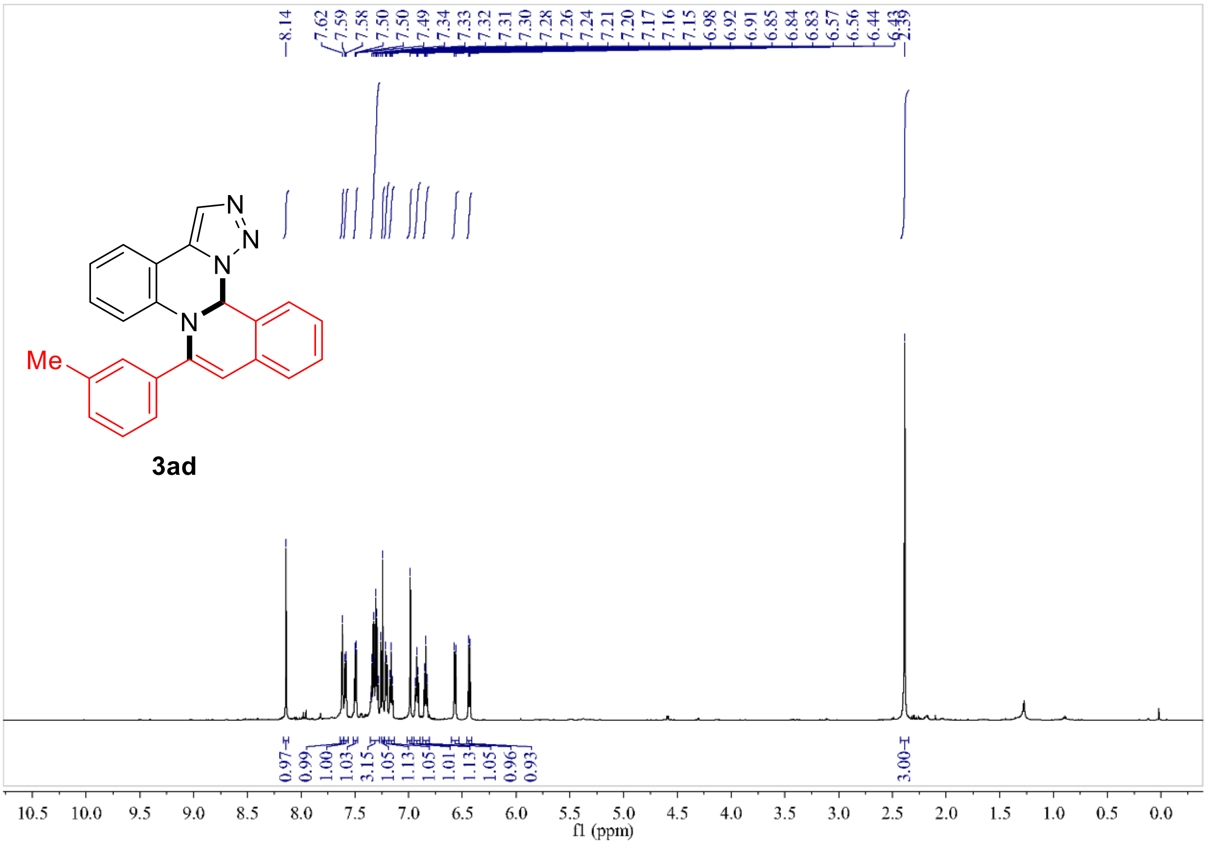
Me
3ad
f1 (ppm)

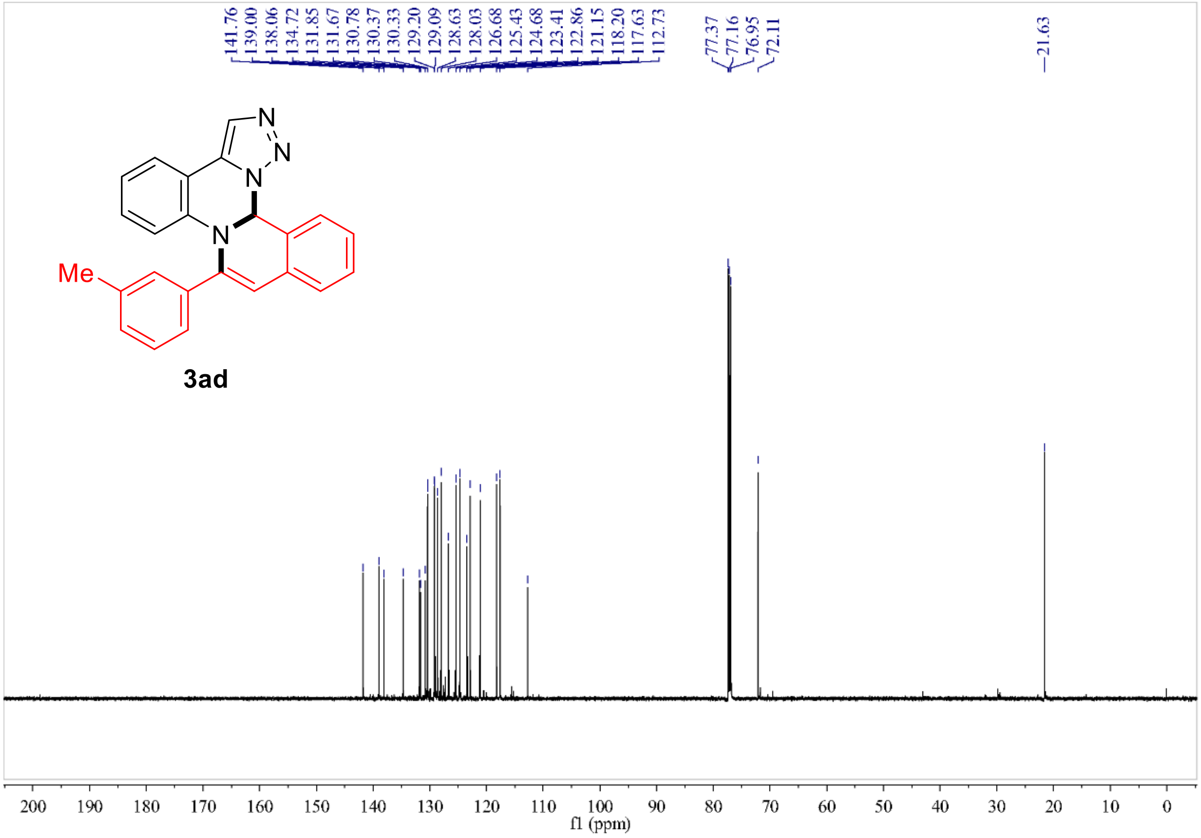
Me
3ad
f1 (ppm)

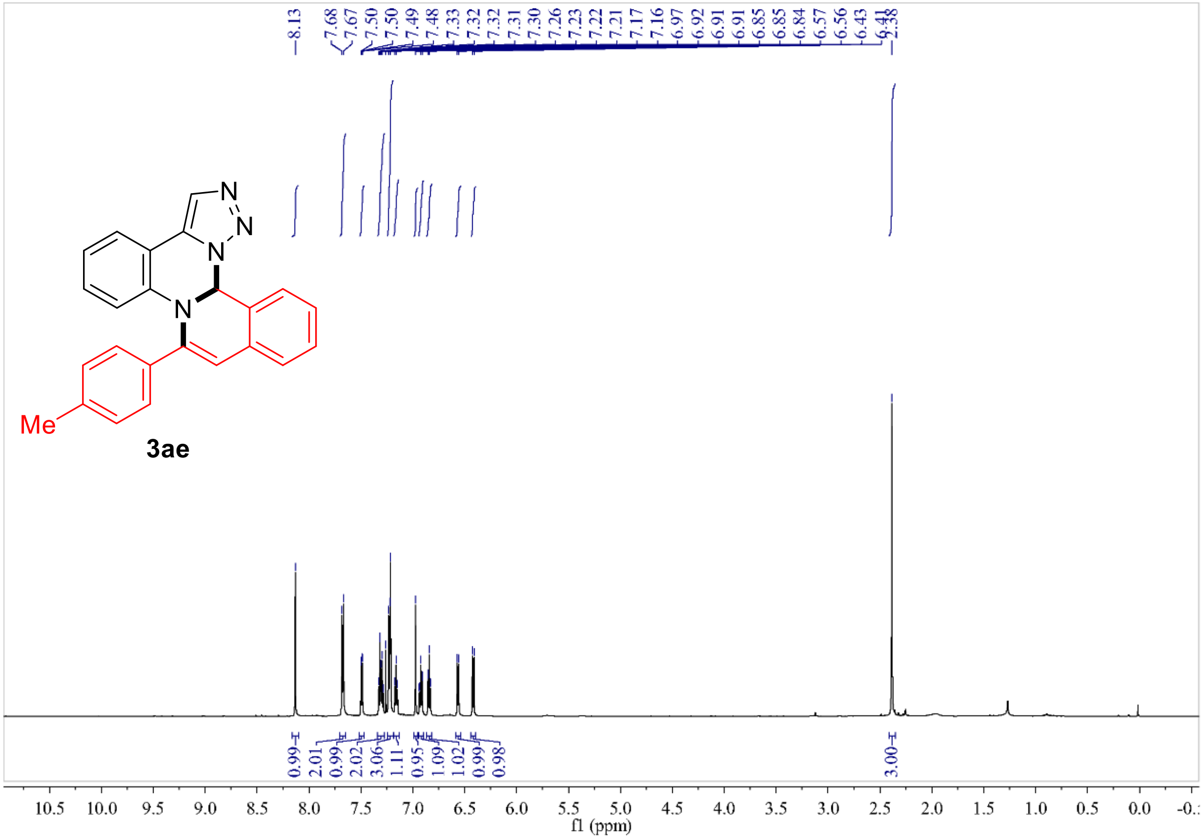
8.13
7.68
7.67
7.50
7.50
7.49
7.48
7.33
7.32
7.32
7.31
7.30
7.26
7.23
7.22
7.21
7.17
7.16
6.97
6.92
6.91
6.91
6.85
6.85
6.84
6.57
6.56
6.43
6.41
2.38
N
N
N
N
Me
3ae
0.99
2.01
0.99
2.02
3.06
1.11
0.95
1.09
1.02
0.99
0.98
3.00
10.5
10.0
9.5
9.0
8.5
8.0
7.5
7.0
6.5
6.0
5.5
5.0
4.5
4.0
3.5
3.0
2.5
2.0
1.5
1.0
0.5
0.0
f1 (ppm)

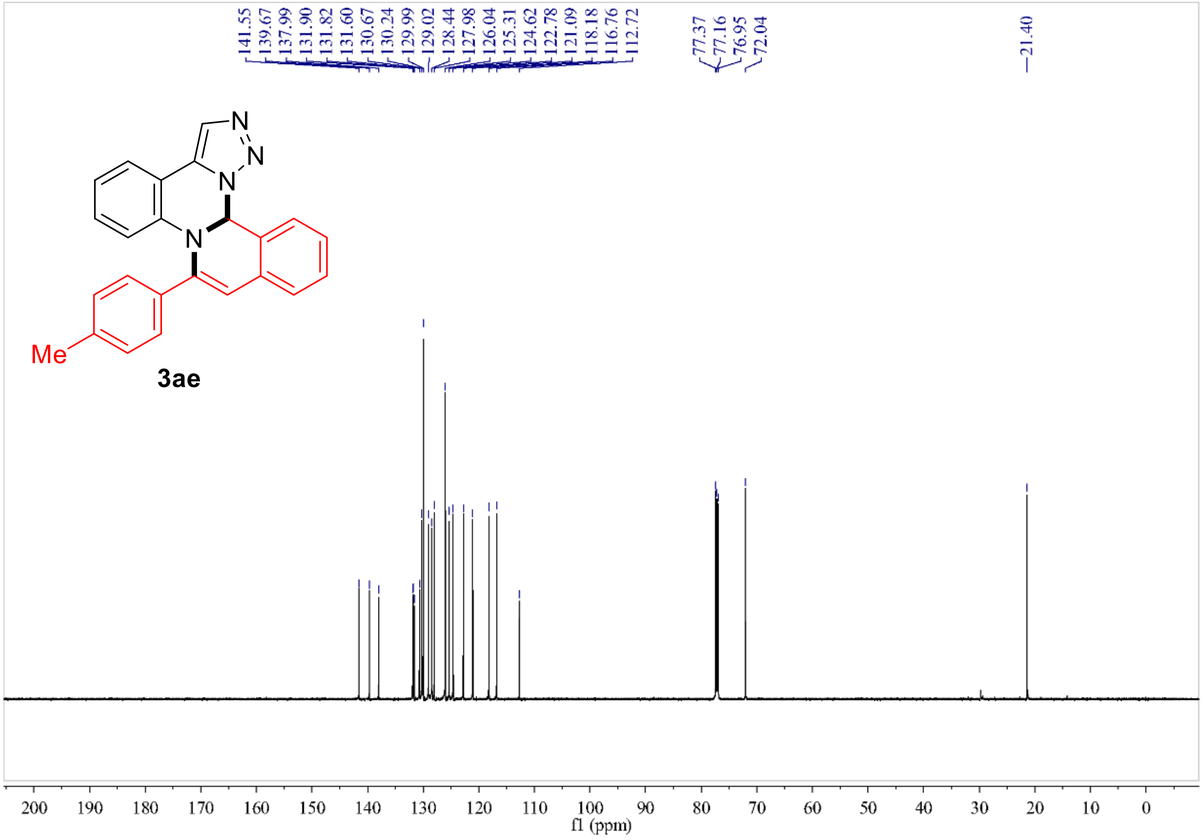
141.55
139.67
137.99
131.90
131.82
131.60
130.67
130.24
129.99
129.02
128.44
127.98
126.04
125.31
124.62
122.78
121.09
118.18
116.76
112.72
77.37
77.16
76.95
72.04
21.40
N
N
N
N
Me
3ae
200
190
180
170
160
150
140
130
120
110
100
90
80
70
60
50
40
30
20
10
0
f1 (ppm)

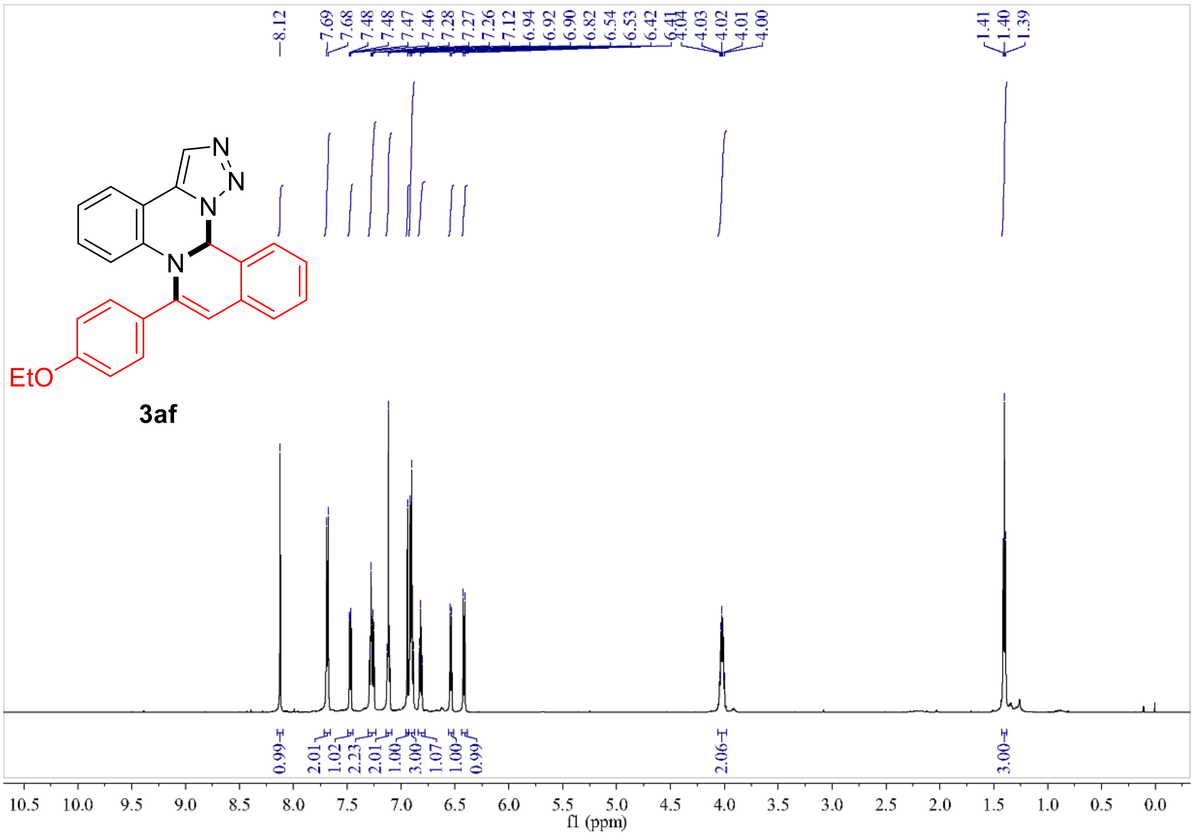
3af
EtO
f1 (ppm)

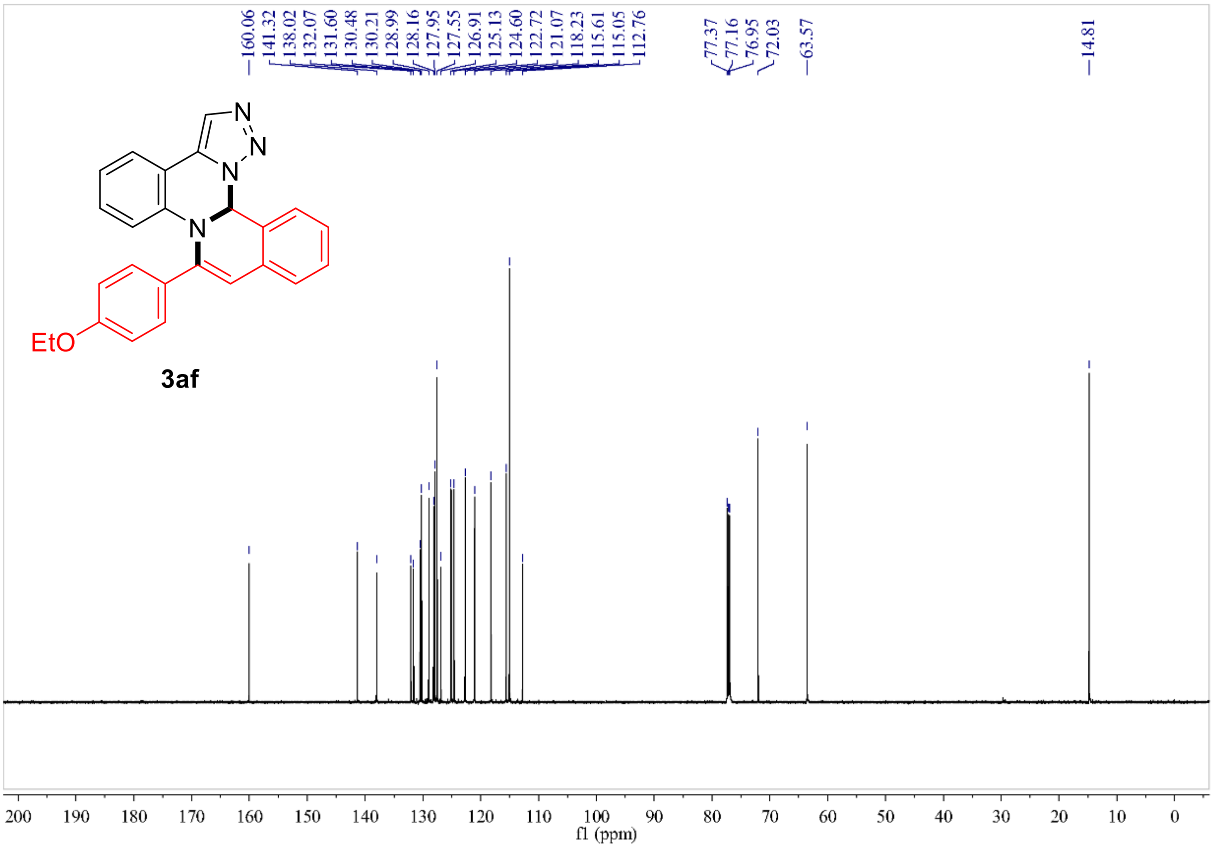
3af
EtO
f1 (ppm)

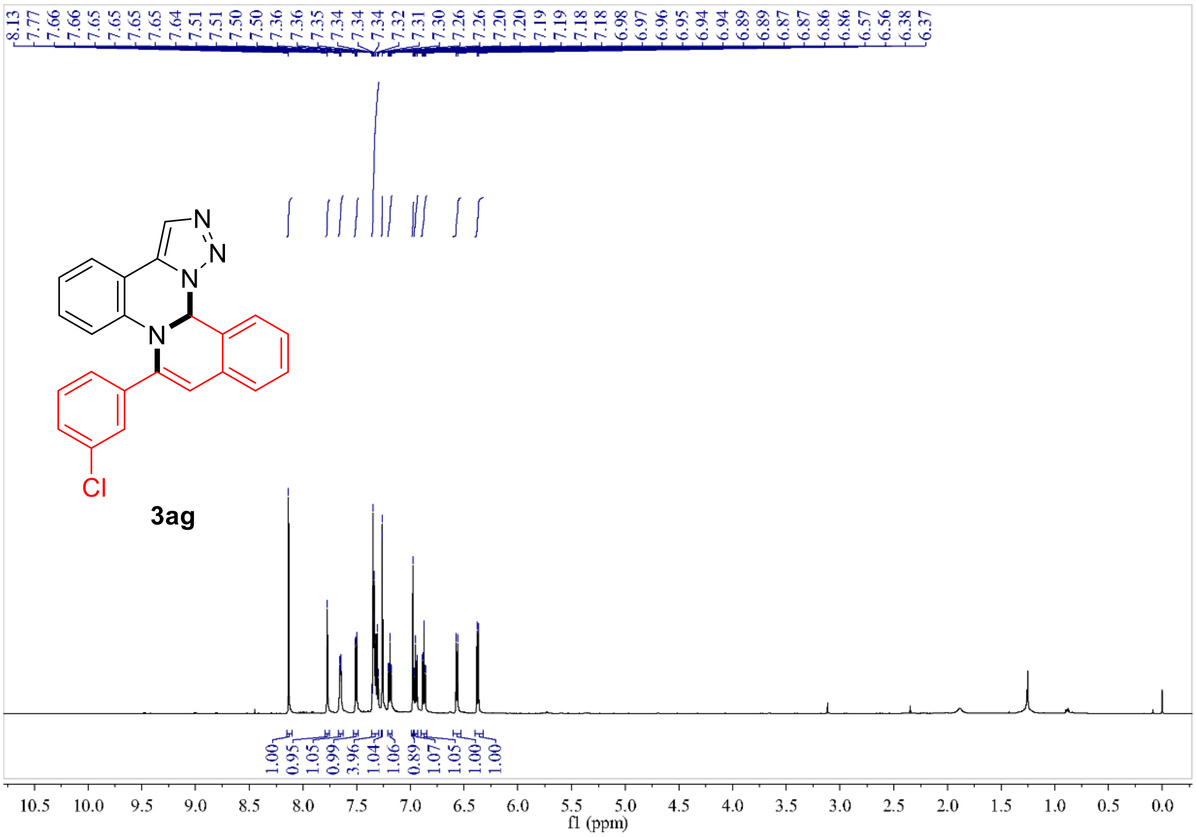
8.13
7.77
7.66
7.66
7.65
7.65
7.65
7.64
7.51
7.51
7.50
7.50
7.36
7.36
7.35
7.34
7.34
7.34
7.32
7.31
7.30
7.26
7.26
7.20
7.20
7.19
7.19
7.18
6.98
6.97
6.96
6.95
6.94
6.94
6.89
6.89
6.87
6.87
6.86
6.86
6.57
6.56
6.38
6.37
N
N
N
N
Cl
3ag
1.00
0.95
1.05
0.99
3.96
1.04
1.06
0.89
1.07
1.05
1.00
1.00
10.5 10.0 9.5 9.0 8.5 8.0 7.5 7.0 6.5 6.0 5.5 5.0 4.5 4.0 3.5 3.0 2.5 2.0 1.5 1.0 0.5 0.0
f1 (ppm)

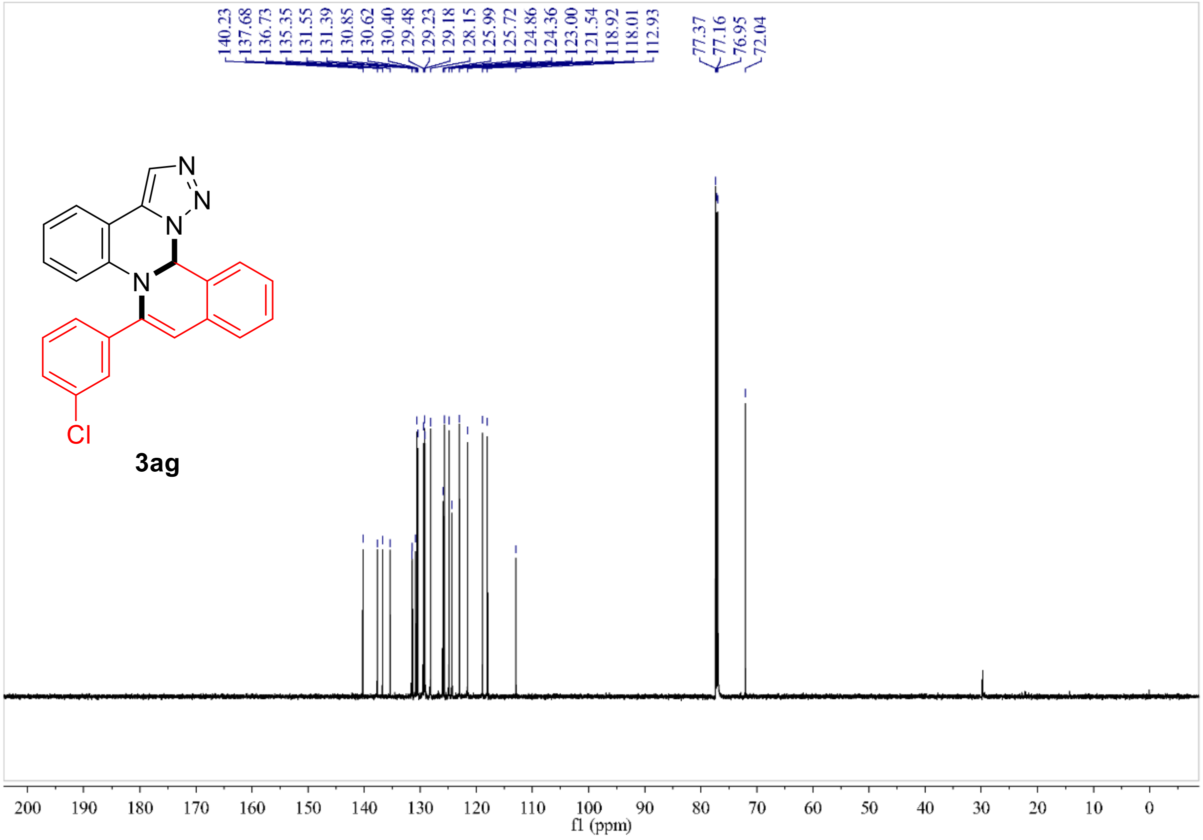
140.23
137.68
136.73
135.35
131.55
131.39
130.85
130.62
130.40
129.48
129.23
129.18
128.15
125.99
125.72
124.86
124.36
123.00
121.54
118.92
118.01
112.93
77.37
77.16
76.95
72.04
N
N
N
N
Cl
3ag
200 190 180 170 160 150 140 130 120 110 100 90 80 70 60 50 40 30 20 10 0
f1 (ppm)

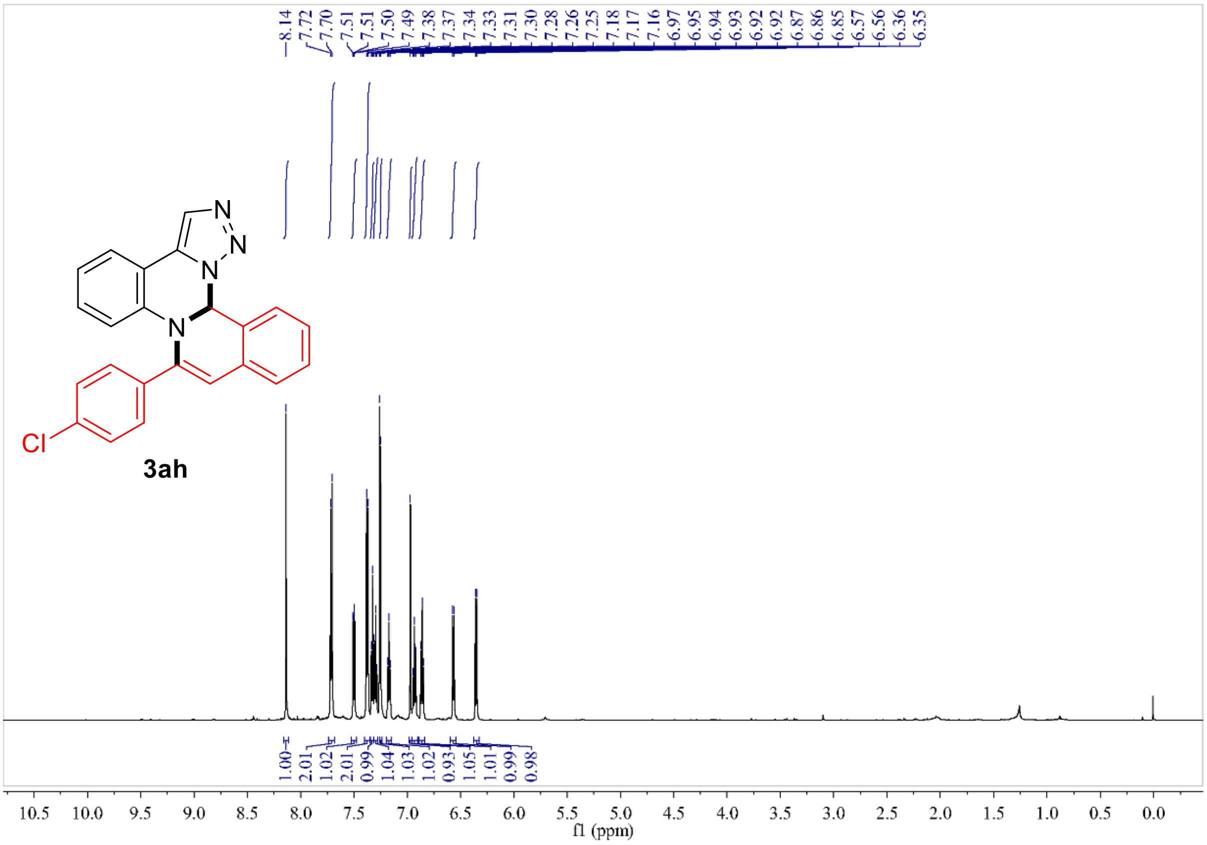
8.14
7.72
7.70
7.51
7.51
7.50
7.49
7.38
7.37
7.34
7.33
7.31
7.30
7.28
7.26
7.25
7.18
7.17
7.16
6.97
6.95
6.94
6.93
6.92
6.92
6.87
6.86
6.85
6.57
6.56
6.36
6.35
N
N
N
N
Cl
3ah
1.00
2.01
1.02
2.01
0.99
1.04
1.03
1.02
0.93
1.05
1.01
0.99
0.98
10.5 10.0 9.5 9.0 8.5 8.0 7.5 7.0 6.5 6.0 5.5 5.0 4.5 4.0 3.5 3.0 2.5 2.0 1.5 1.0 0.5 0.0
f1 (ppm)

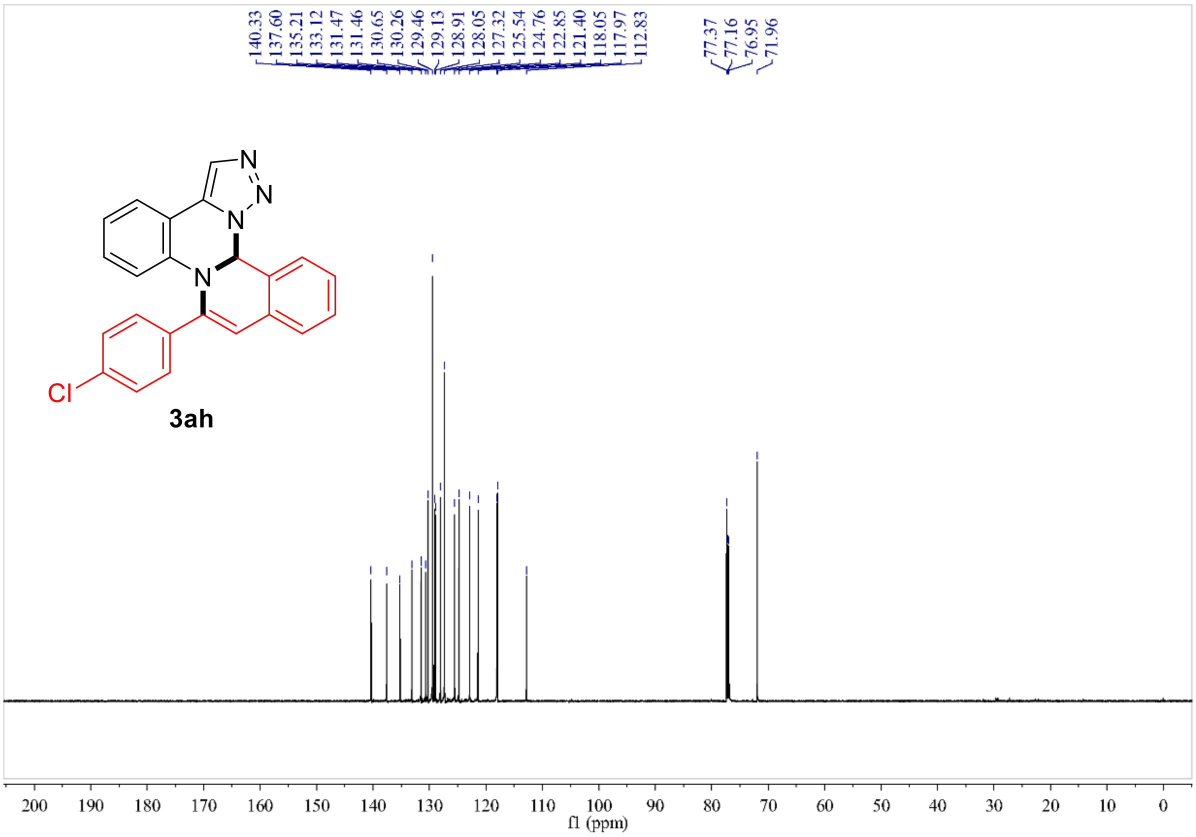
140.33
137.60
135.21
133.12
131.47
131.46
130.65
130.26
129.46
129.13
128.91
128.05
127.32
125.54
124.76
122.85
121.40
118.05
117.97
112.83
77.37
77.16
76.95
71.96
N
N
N
N
Cl
3ah
200 190 180 170 160 150 140 130 120 110 100 90 80 70 60 50 40 30 20 10 0
f1 (ppm)

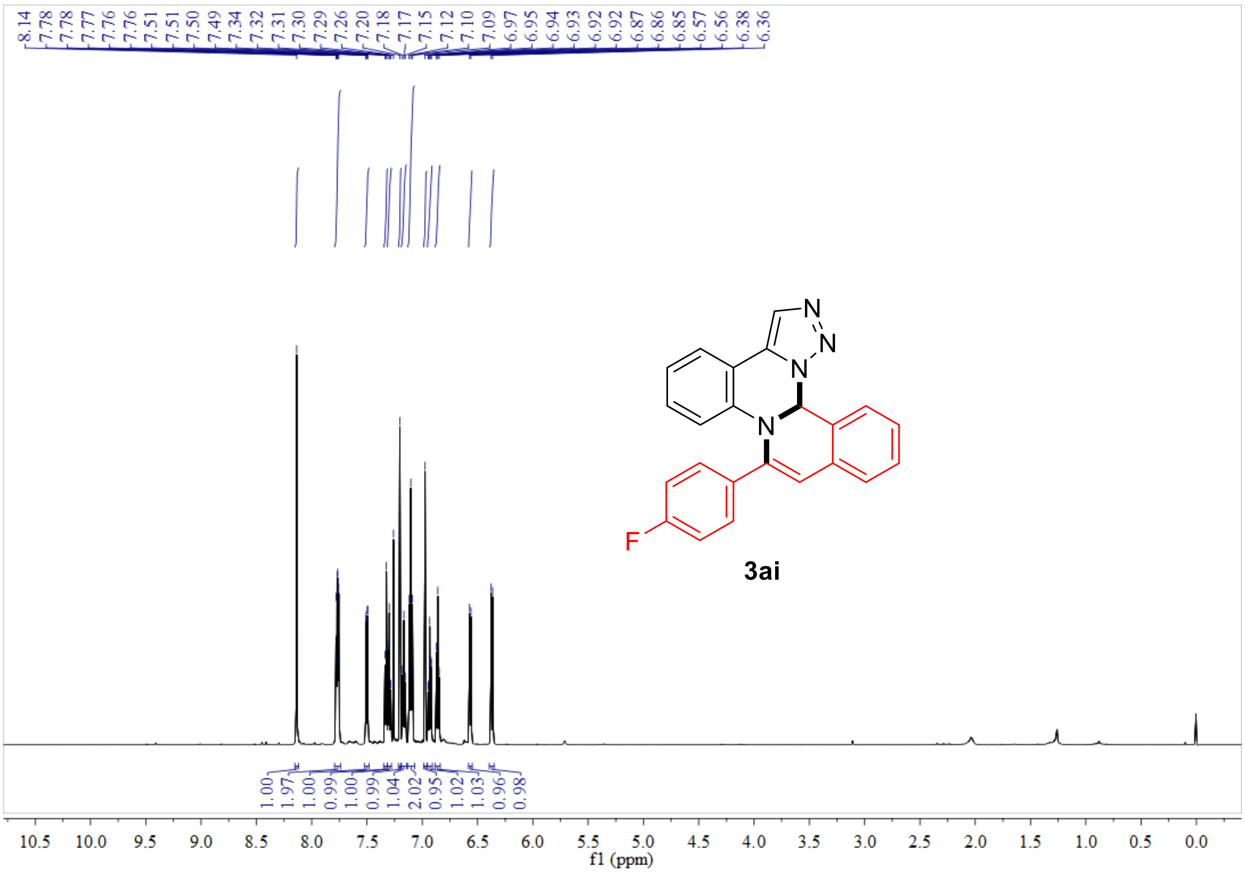

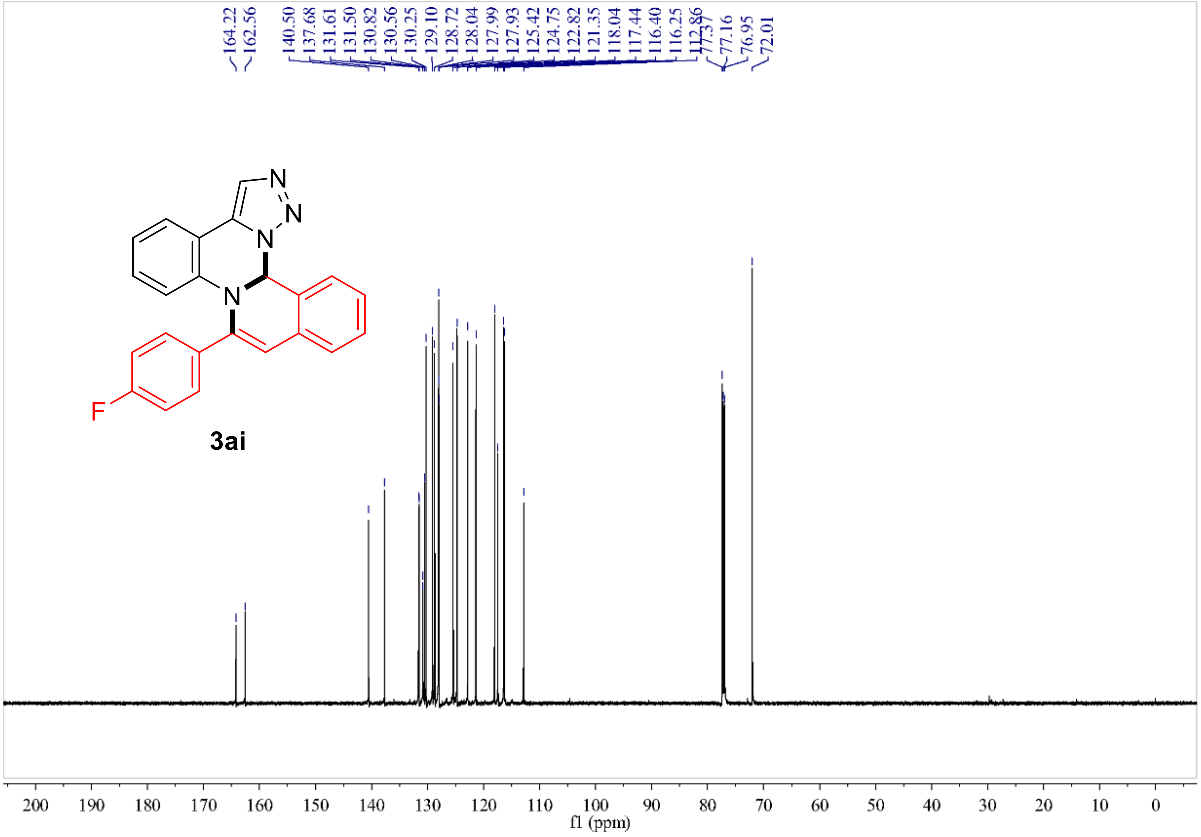

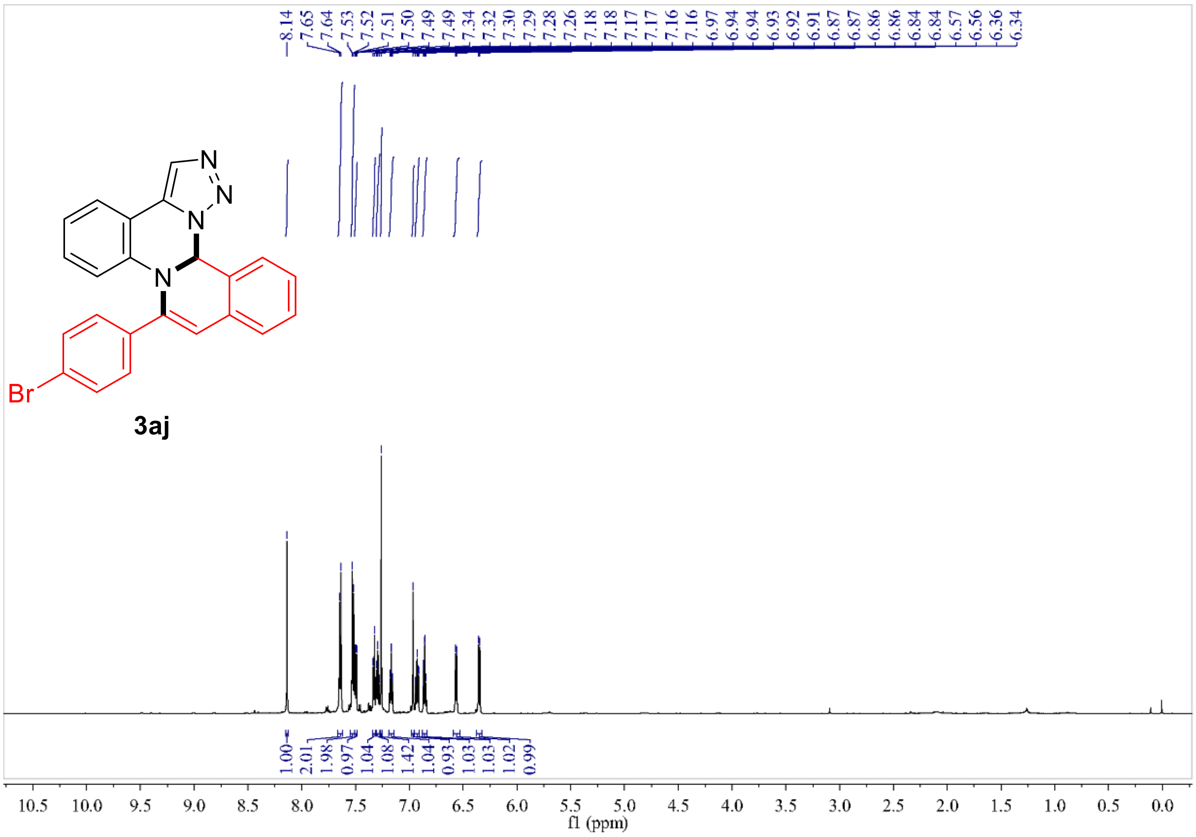

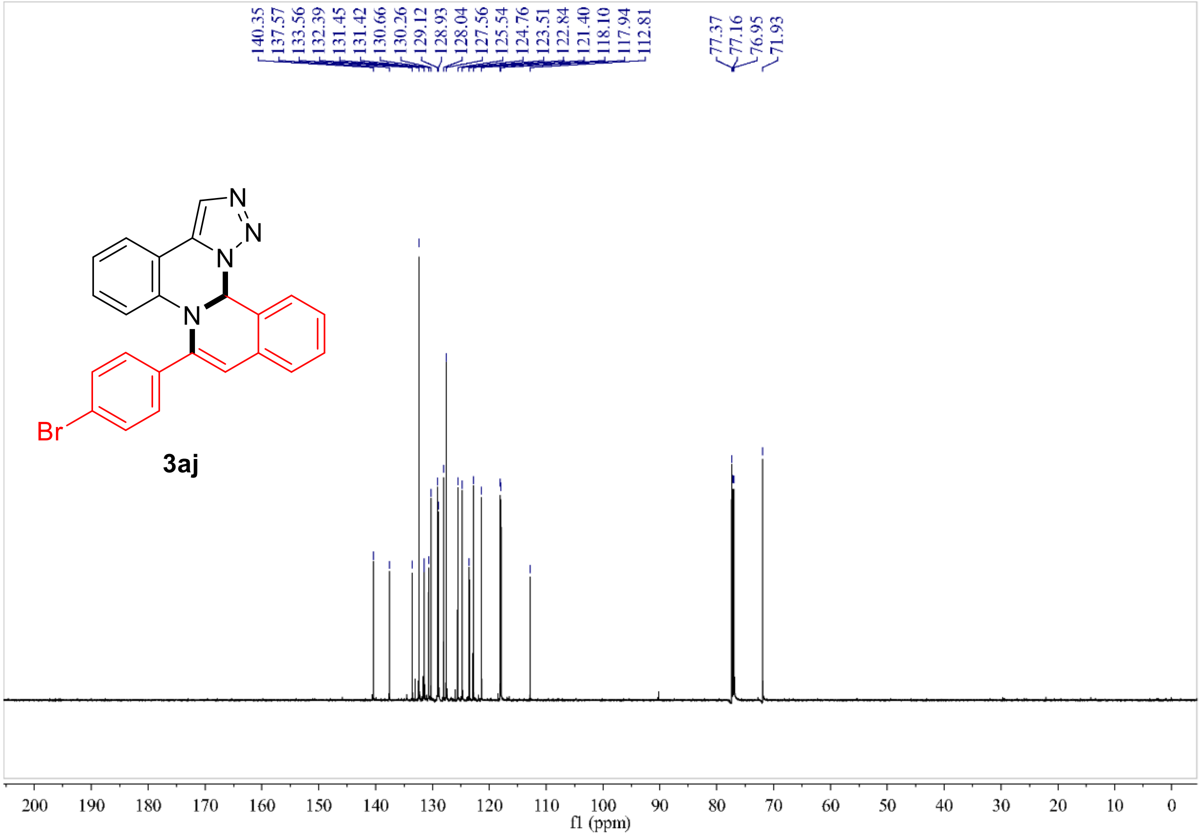

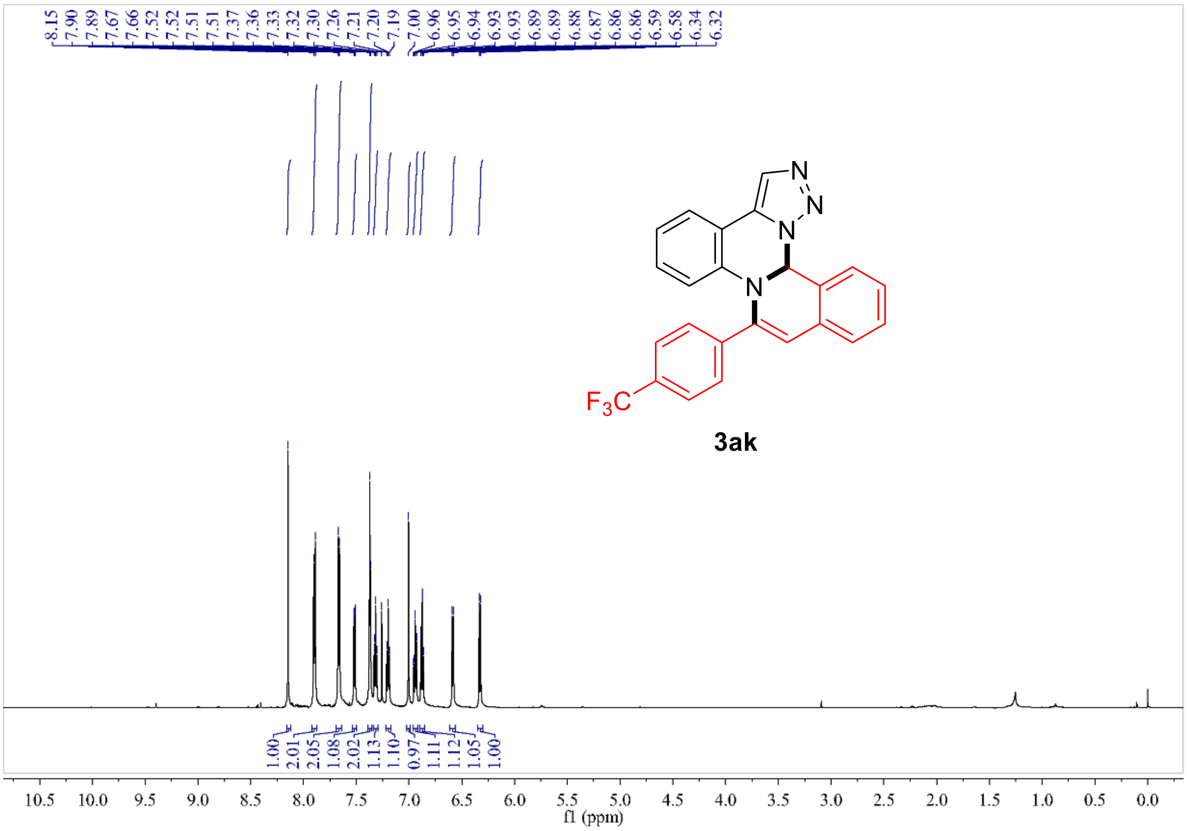

3ak
F3C
N
f1 (ppm)

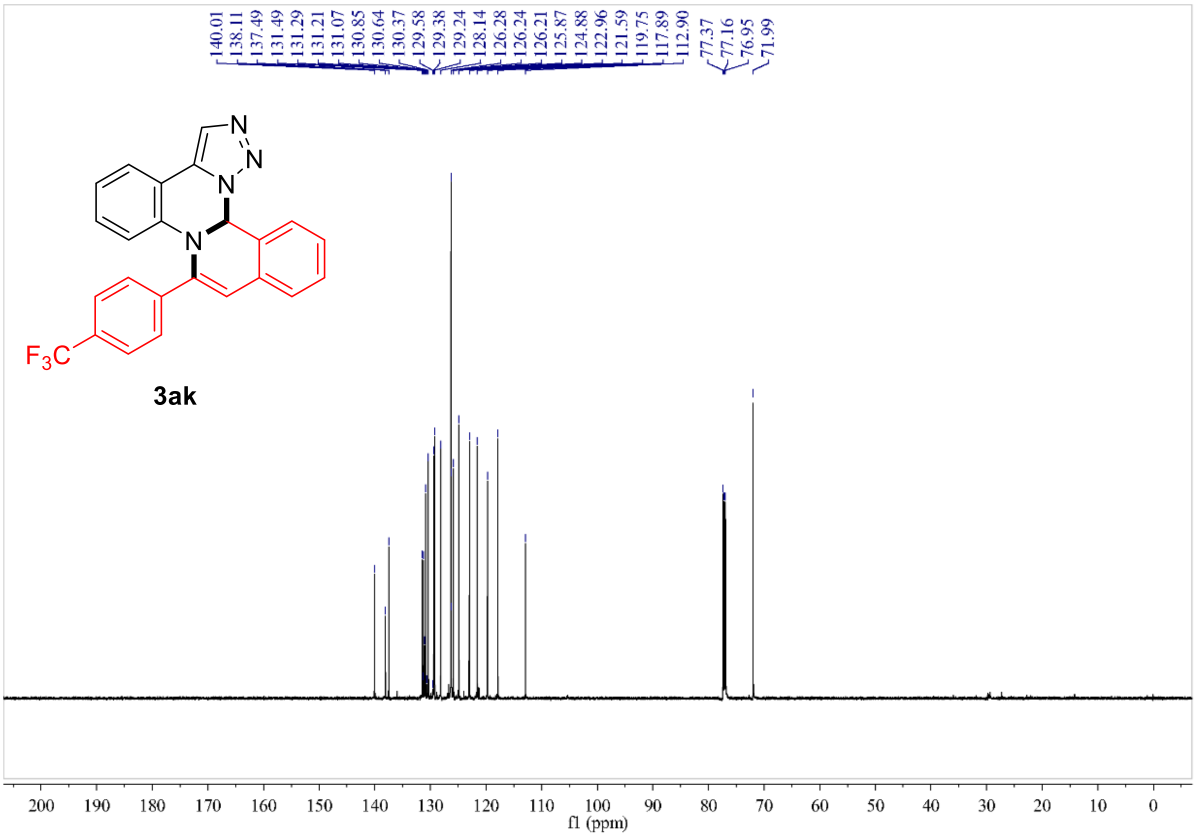

3ak
F3C
N
f1 (ppm)

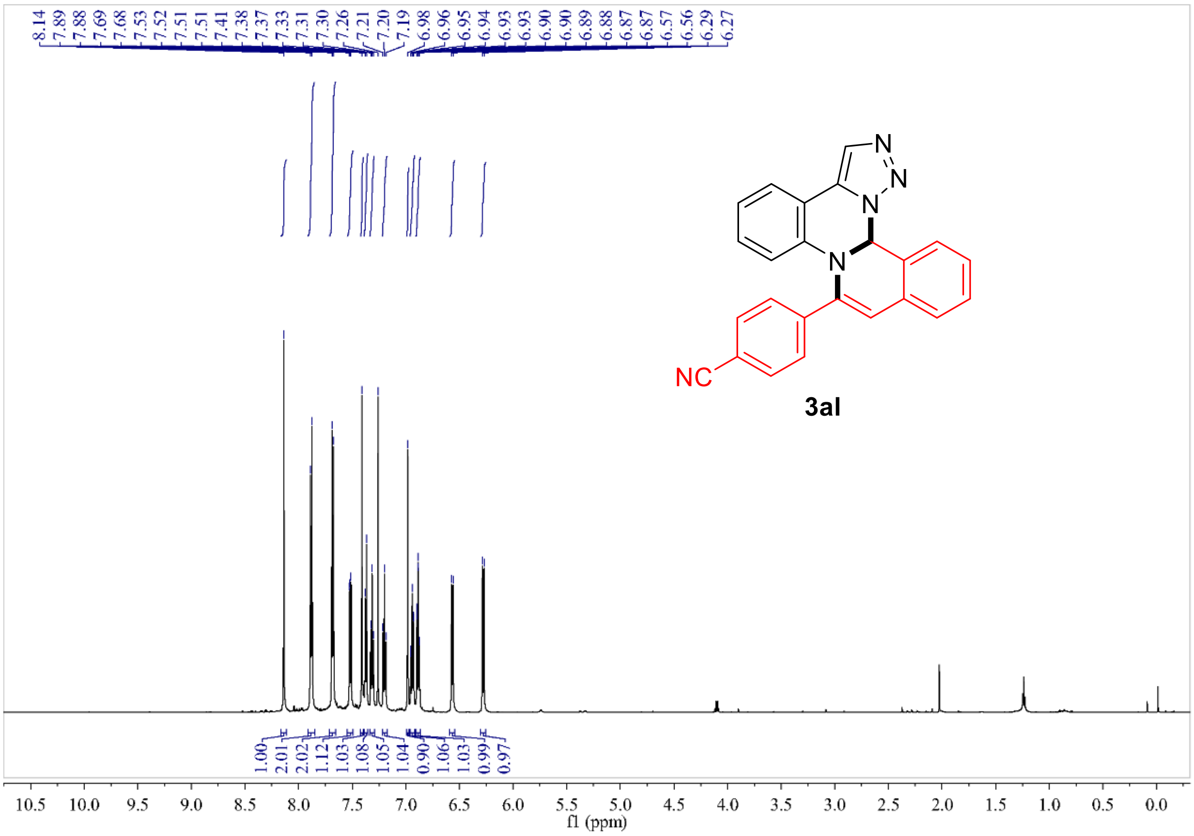
8.14 7.89 7.88 7.69 7.68 7.53 7.52 7.51 7.51 7.41 7.38 7.37 7.33 7.31 7.30 7.26 7.21 7.20 7.19 6.98 6.96 6.95 6.94 6.93 6.93 6.90 6.90 6.89 6.88 6.87 6.87 6.57 6.56 6.29 6.27
N
N
N
N
NC
3al
1.00 2.01 2.02 1.12 1.03 1.08 1.05 1.04 0.90 1.06 1.03 0.99 0.97
10.5 10.0 9.5 9.0 8.5 8.0 7.5 7.0 6.5 6.0 5.5 5.0 4.5 4.0 3.5 3.0 2.5 2.0 1.5 1.0 0.5 0.0
f1 (ppm)

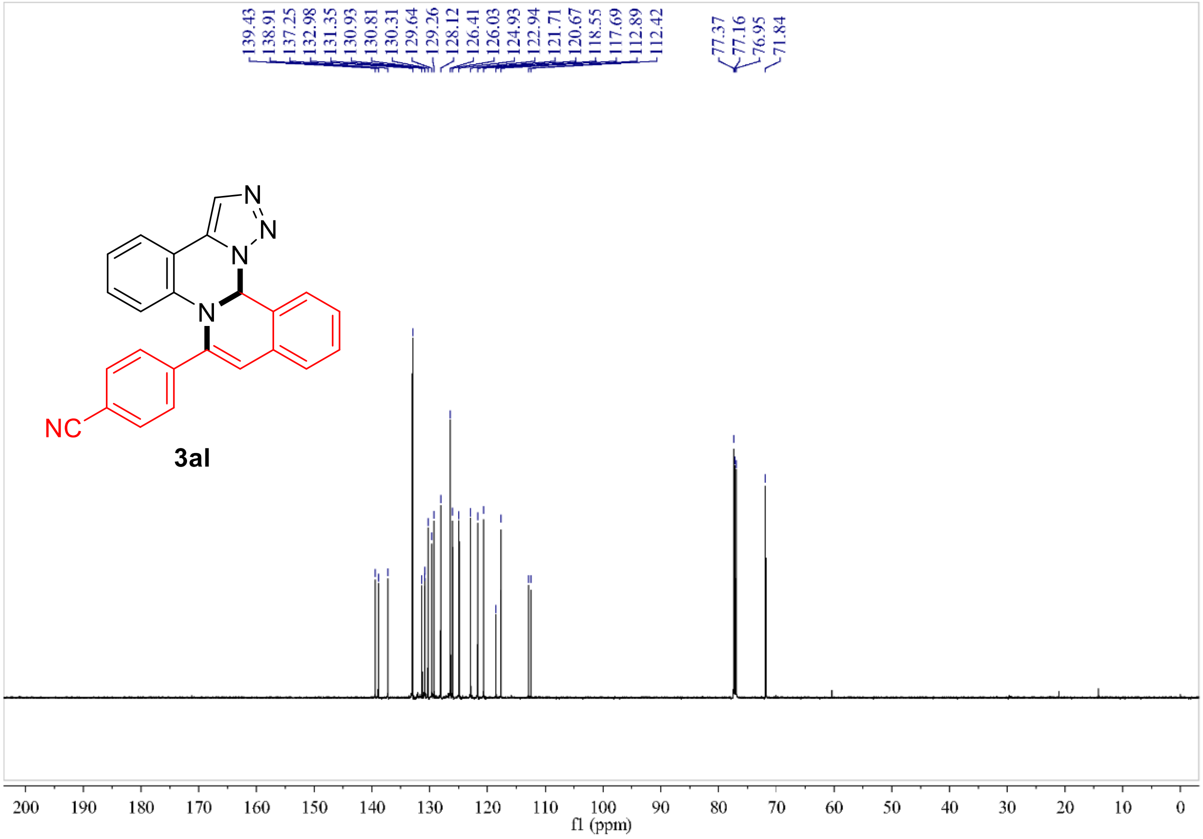
139.43 138.91 137.25 132.98 131.35 130.93 130.81 130.31 129.64 129.26 128.12 126.41 126.03 124.93 122.94 121.71 120.67 118.55 117.69 112.89 112.42
77.37 77.16 76.95 71.84
N
N
N
N
NC
3al
200 190 180 170 160 150 140 130 120 110 100 90 80 70 60 50 40 30 20 10 0
f1 (ppm)

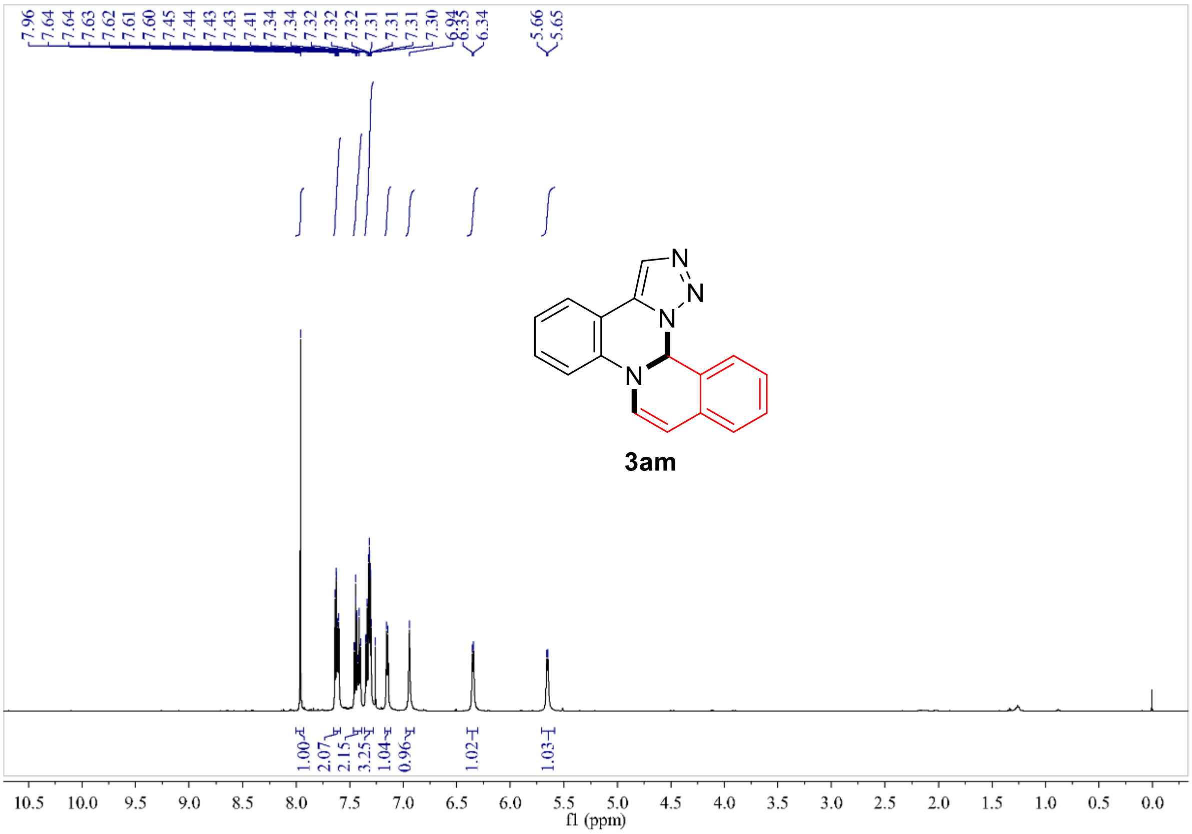
7.96
7.64
7.64
7.63
7.62
7.61
7.60
7.45
7.44
7.43
7.43
7.41
7.34
7.34
7.32
7.32
7.32
7.31
7.31
7.31
7.30
6.94
6.35
6.34
5.66
5.65
3am
1.00
2.07
2.15
3.25
1.04
0.96
1.02
1.03
f1 (ppm)

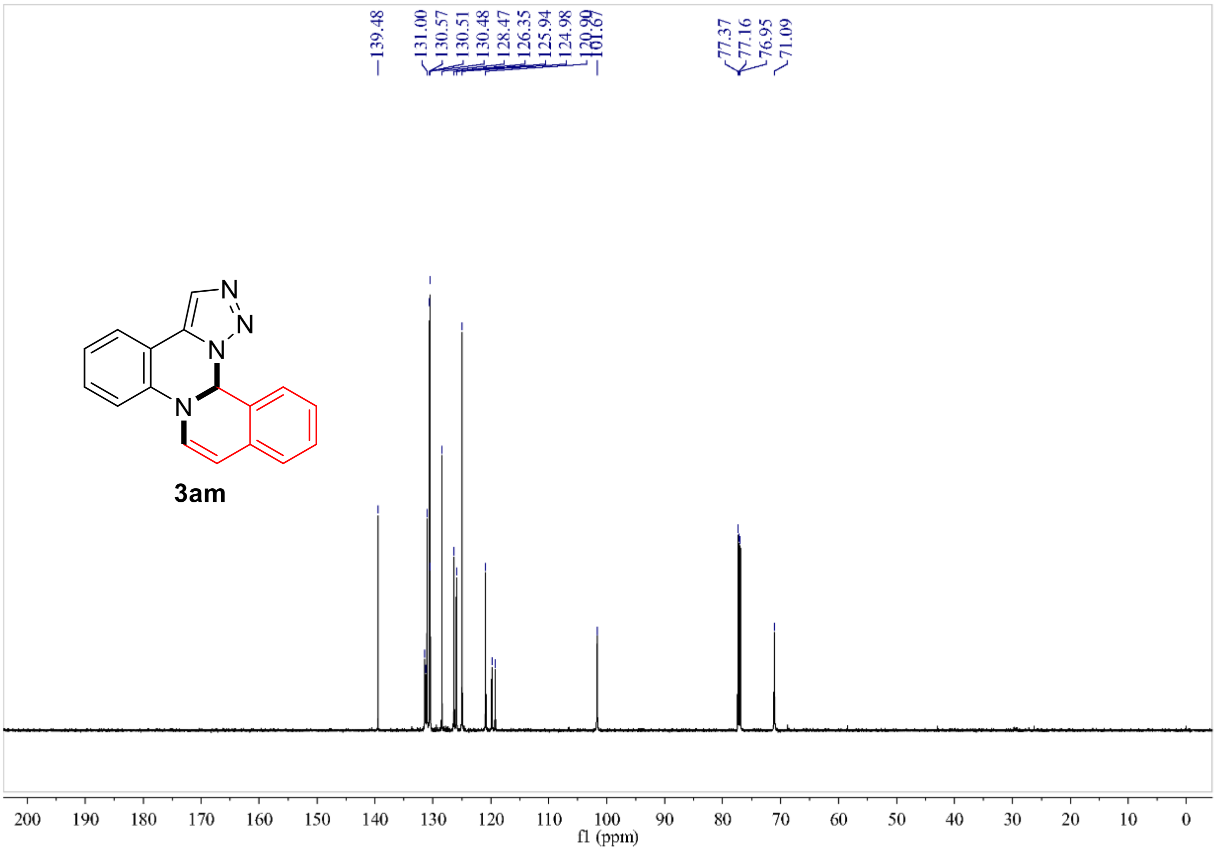
139.48
131.00
130.57
130.51
130.48
128.47
126.35
125.94
124.98
120.99
101.67
77.37
77.16
76.95
71.09
3am
f1 (ppm)

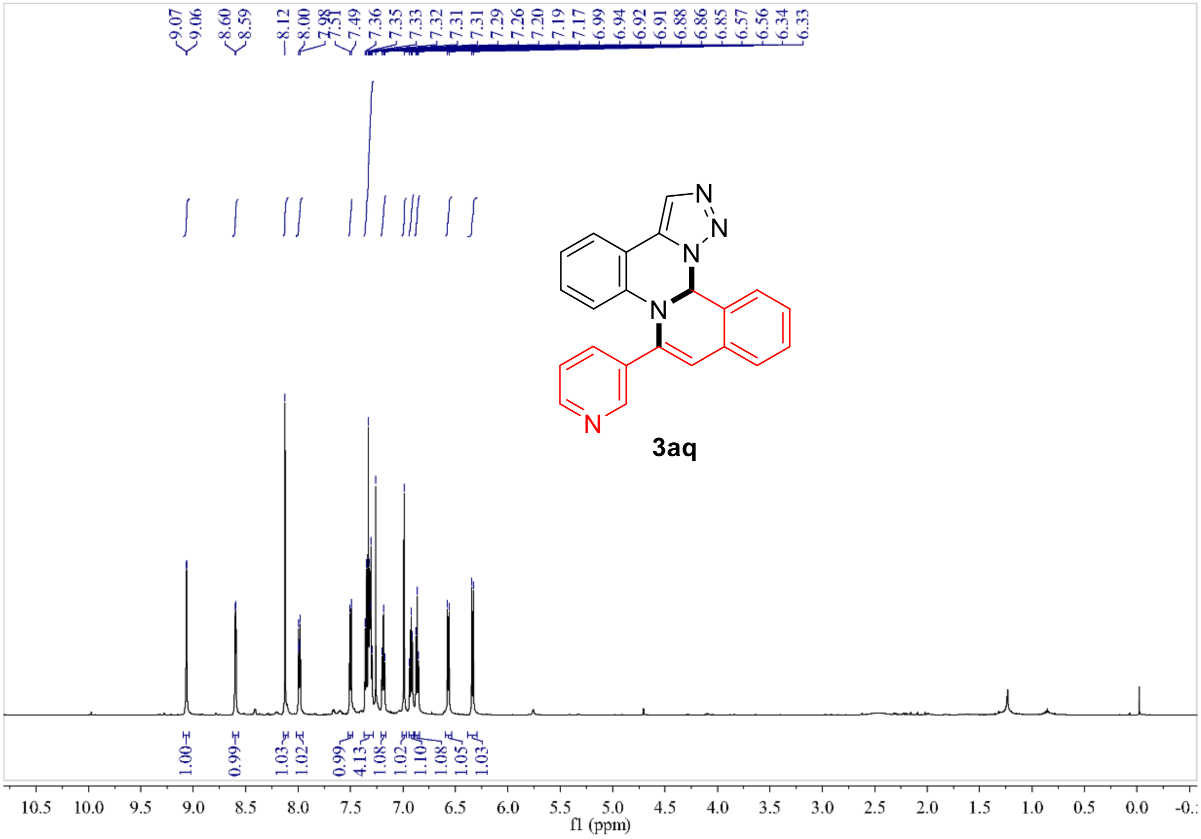
9.07
9.06
8.60
8.59
8.12
8.00
7.98
7.51
7.49
7.36
7.35
7.33
7.32
7.31
7.31
7.29
7.26
7.20
7.19
7.17
6.99
6.94
6.92
6.91
6.88
6.86
6.85
6.57
6.56
6.34
6.33
N
N
N
N
N
3aq
1.00
0.99
1.03
1.02
0.99
4.13
1.08
1.02
1.10
1.08
1.05
1.03
f1 (ppm)

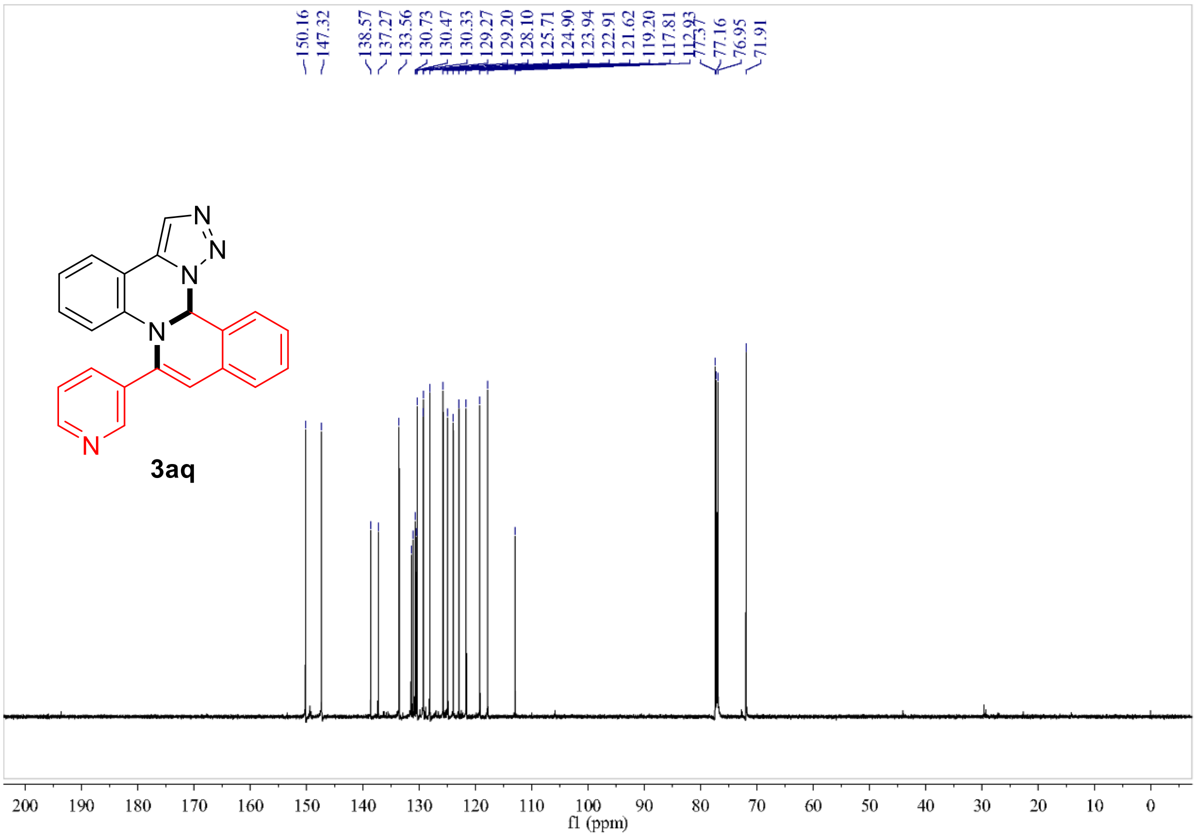
150.16
147.32
138.57
137.27
133.56
130.73
130.47
130.33
129.27
129.20
128.10
125.71
124.90
123.94
122.91
121.62
119.20
117.81
112.93
77.37
77.16
76.95
71.91
N
N
N
N
N
3aq
f1 (ppm)

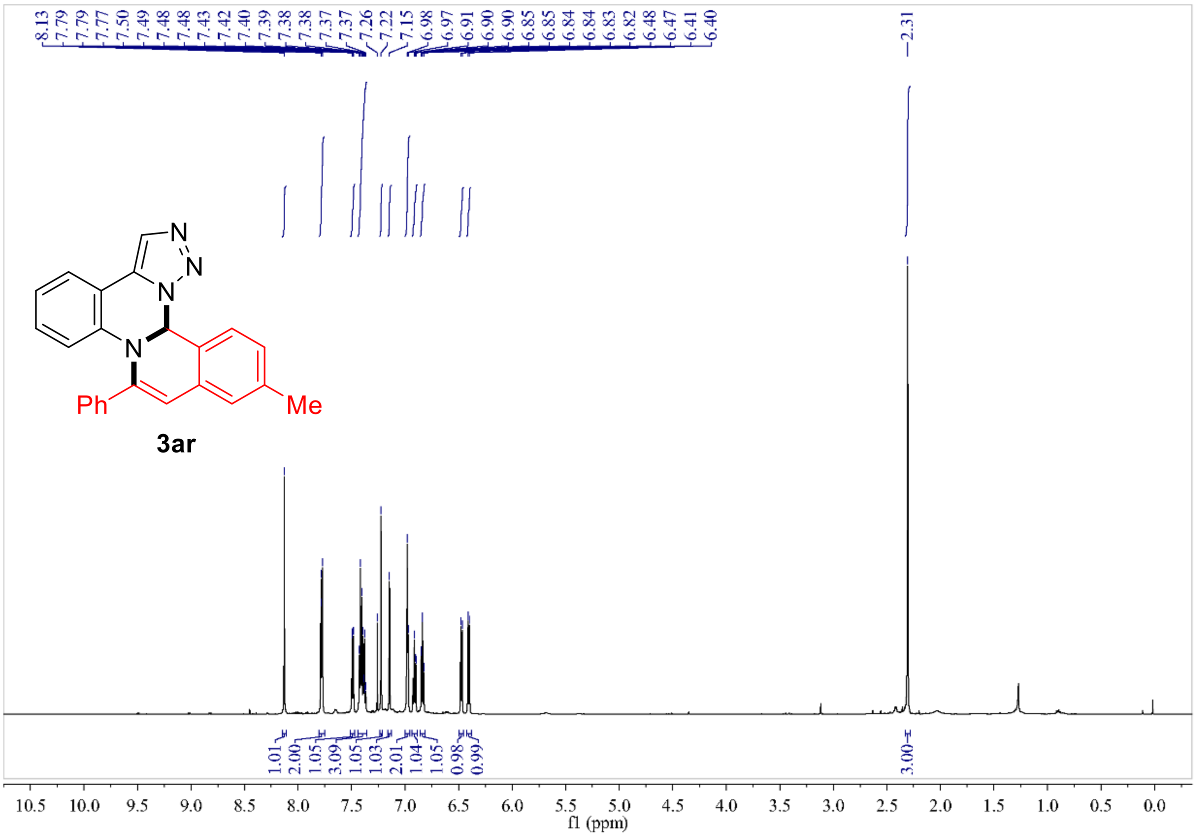
8.13
7.79
7.79
7.77
7.50
7.49
7.48
7.48
7.43
7.42
7.40
7.39
7.38
7.38
7.37
7.37
7.26
7.22
7.15
6.98
6.97
6.91
6.90
6.90
6.85
6.85
6.84
6.84
6.83
6.82
6.48
6.47
6.41
6.40
2.31
N
N
N
N
Ph
Me
3ar
1.01
2.00
1.05
3.09
1.05
1.03
2.01
1.04
1.05
0.98
0.99
3.00
f1 (ppm)

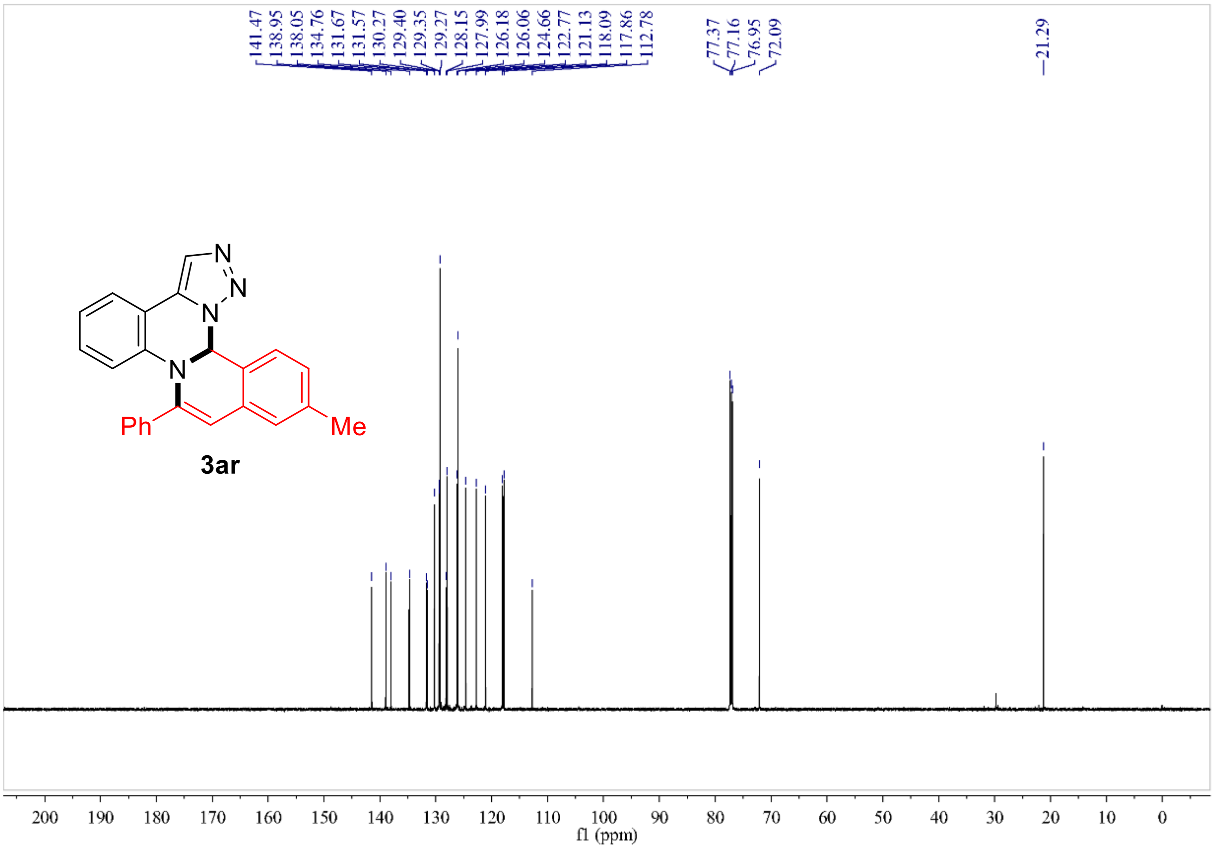
141.47
138.95
138.05
134.76
131.67
131.57
130.27
129.40
129.35
129.27
128.15
127.99
126.18
126.06
124.66
122.77
121.13
118.09
117.86
112.78
77.37
77.16
76.95
72.09
21.29
N
N
N
N
Ph
Me
3ar
f1 (ppm)

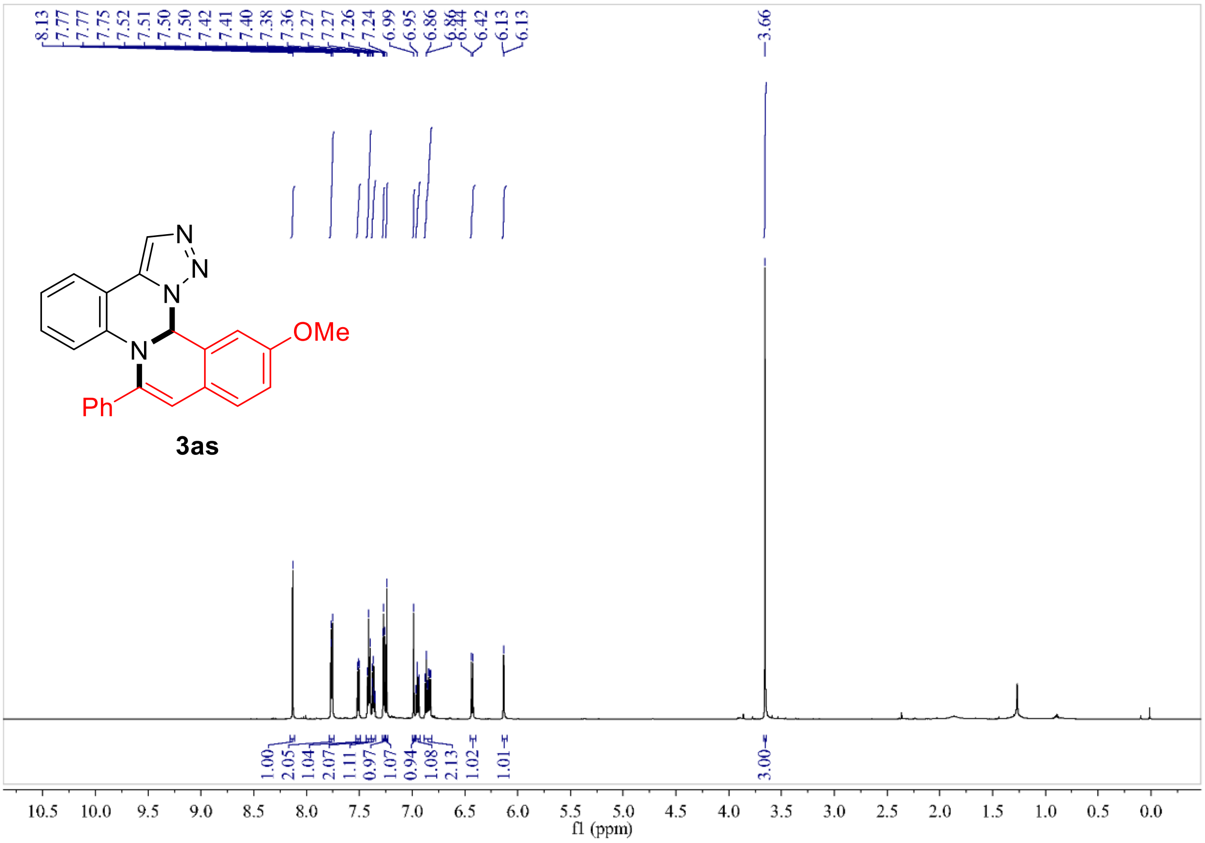

8.13 7.77 7.77 7.75 7.52 7.51 7.50 7.50 7.42 7.41 7.40 7.38 7.36 7.27 7.27 7.26 7.24 6.99 6.95 6.86 6.86 6.44 6.42 6.13 6.13
3.66
OMe
Ph
3as
1.00 2.05 1.04 2.07 1.11 0.97 1.07 0.94 1.08 2.13 1.02 1.01
3.00
10.5 10.0 9.5 9.0 8.5 8.0 7.5 7.0 6.5 6.0 5.5 5.0 4.5 4.0 3.5 3.0 2.5 2.0 1.5 1.0 0.5 0.0
f1 (ppm)

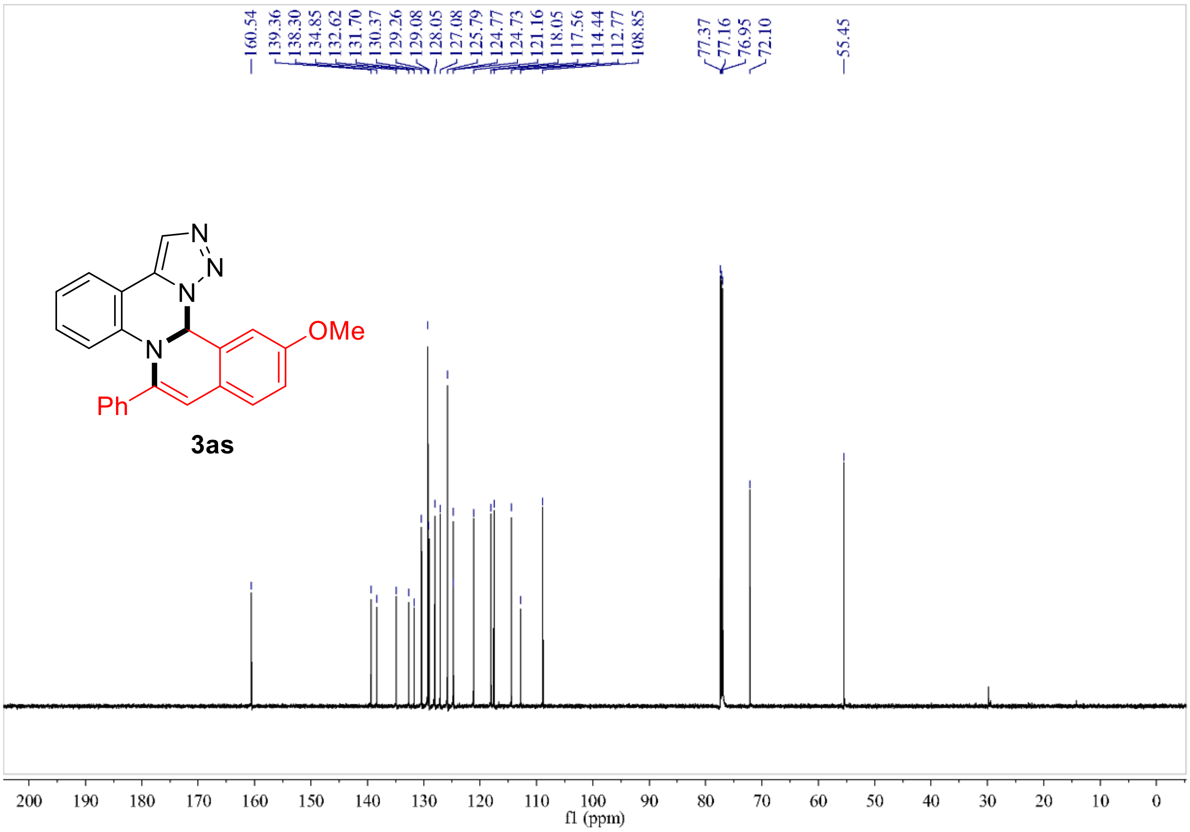

160.54 139.36 138.30 134.85 132.62 131.70 130.37 129.26 129.08 128.05 127.08 125.79 124.77 124.73 121.16 118.05 117.56 114.44 112.77 108.85
77.37 77.16 76.95 72.10
55.45
OMe
Ph
3as
200 190 180 170 160 150 140 130 120 110 100 90 80 70 60 50 40 30 20 10 0
f1 (ppm)

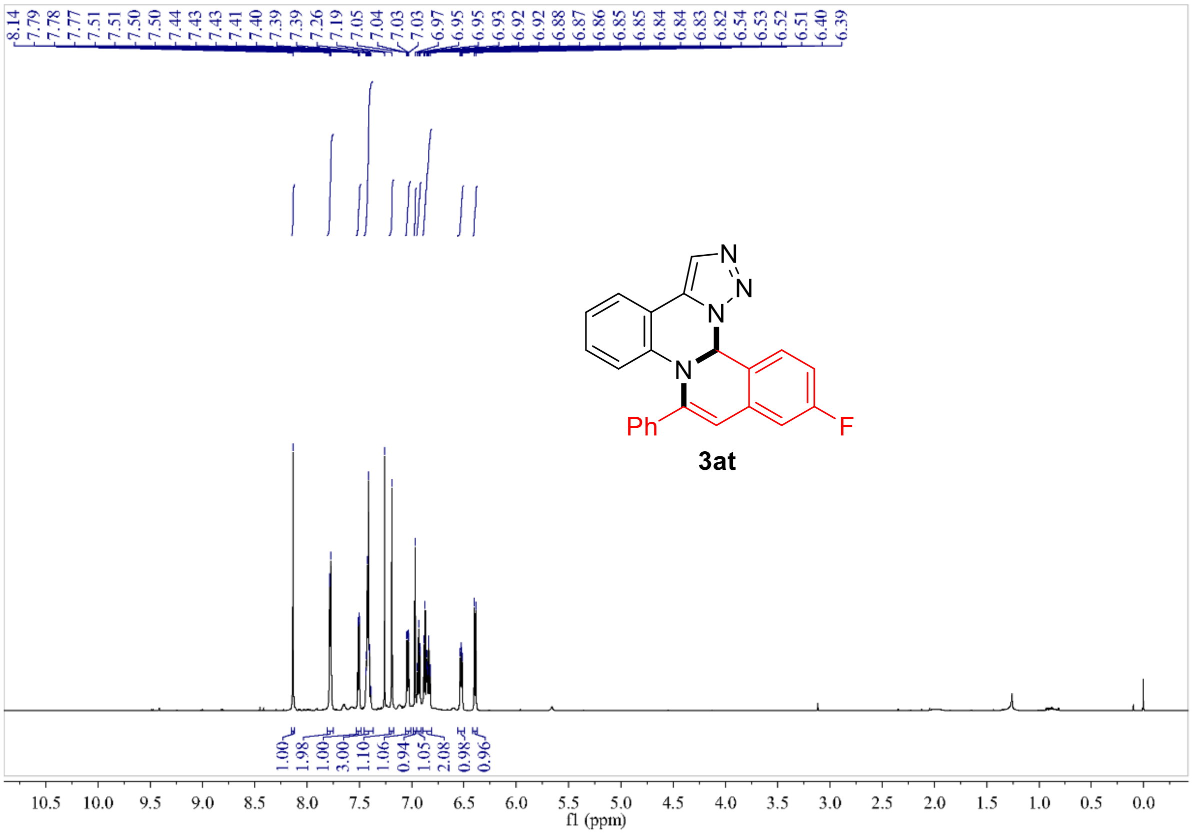

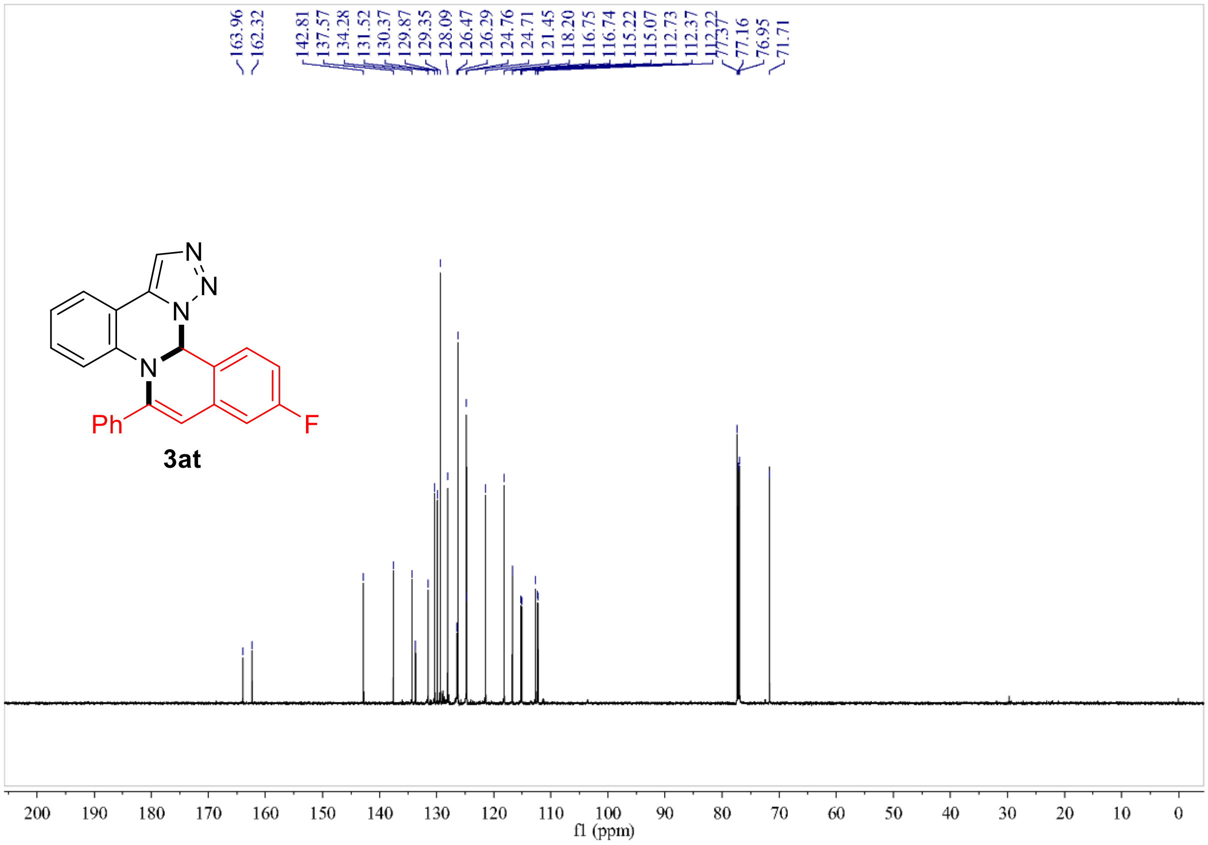

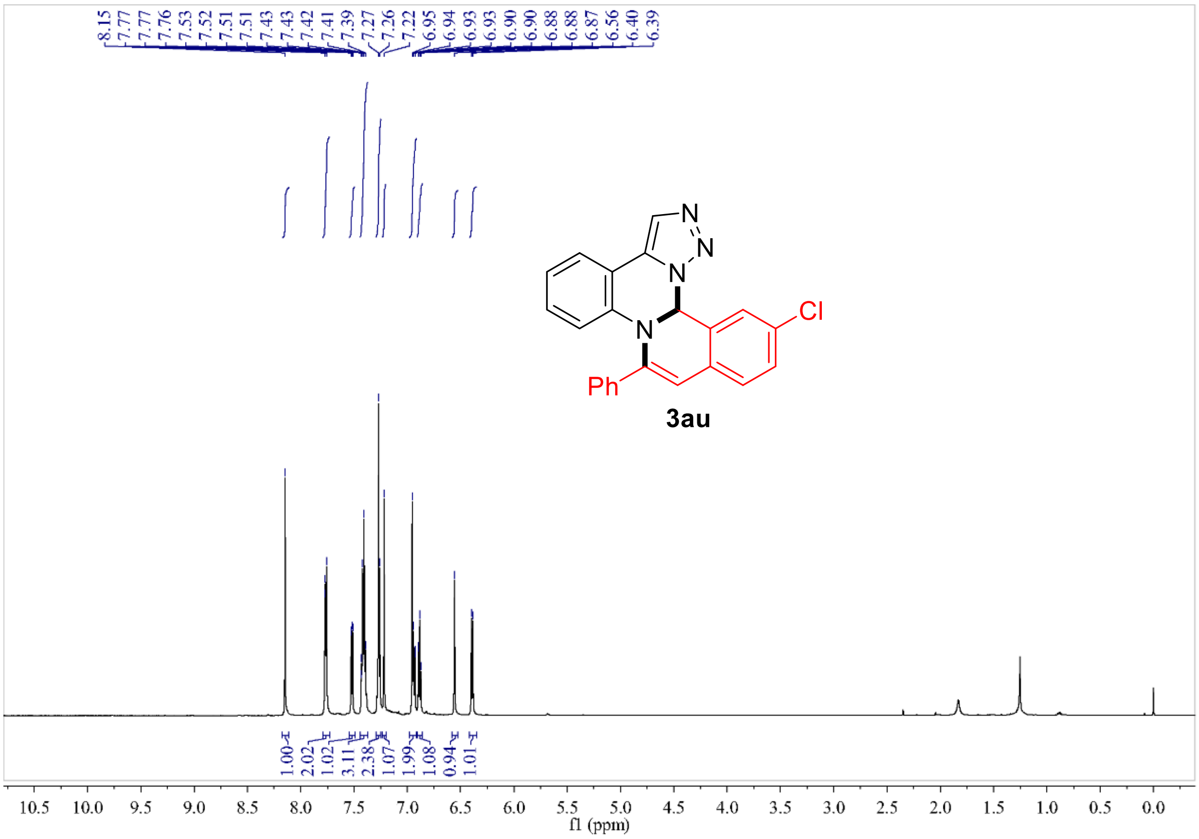
8.15
7.77
7.77
7.76
7.53
7.52
7.51
7.51
7.43
7.43
7.42
7.41
7.39
7.27
7.26
7.22
6.95
6.94
6.93
6.93
6.90
6.90
6.88
6.88
6.87
6.56
6.40
6.39
N
N
N
N
Cl
Ph
3au
1.00
2.02
1.02
3.11
2.38
1.07
1.99
1.08
0.94
1.01
10.5 10.0 9.5 9.0 8.5 8.0 7.5 7.0 6.5 6.0 5.5 5.0 4.5 4.0 3.5 3.0 2.5 2.0 1.5 1.0 0.5 0.0
f1 (ppm)

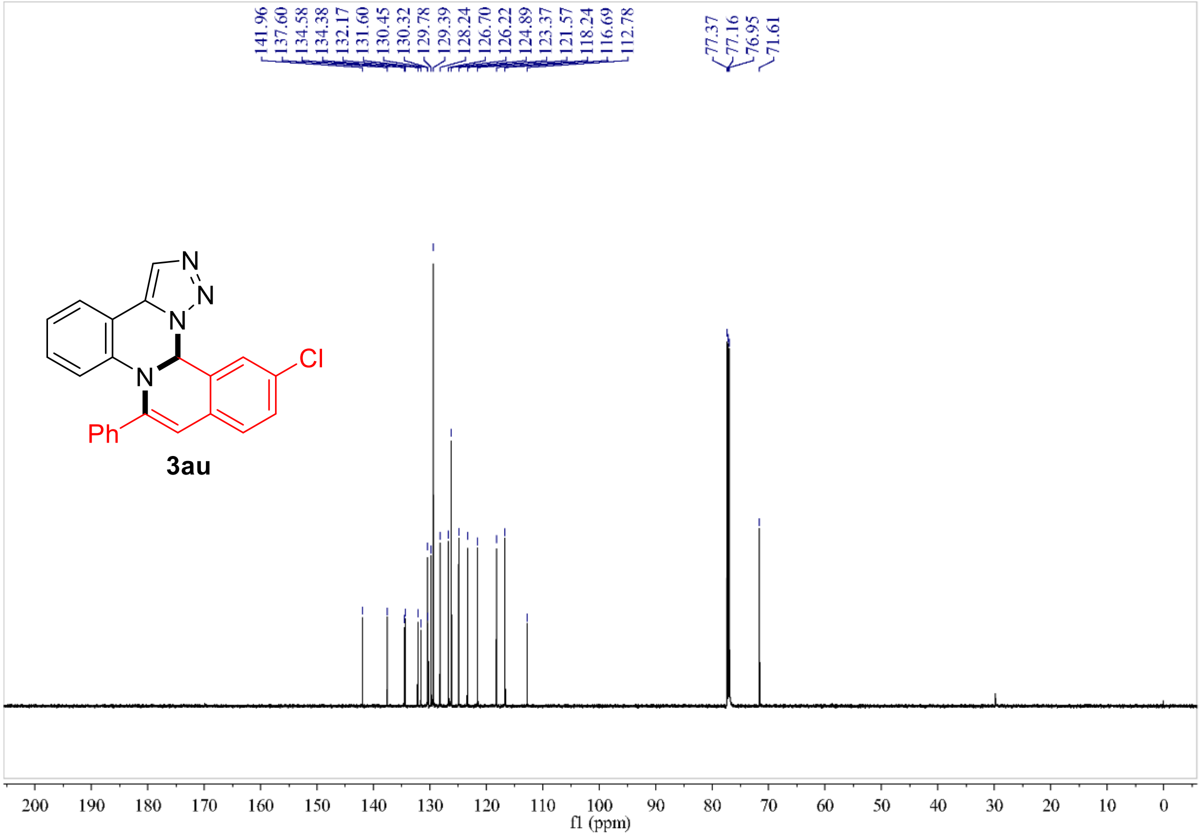
141.96
137.60
134.58
134.38
132.17
131.60
130.45
130.32
129.78
129.39
128.24
126.70
126.22
124.89
123.37
121.57
118.24
116.69
112.78
77.37
77.16
76.95
71.61
N
N
N
N
Cl
Ph
3au
200 190 180 170 160 150 140 130 120 110 100 90 80 70 60 50 40 30 20 10 0
f1 (ppm)

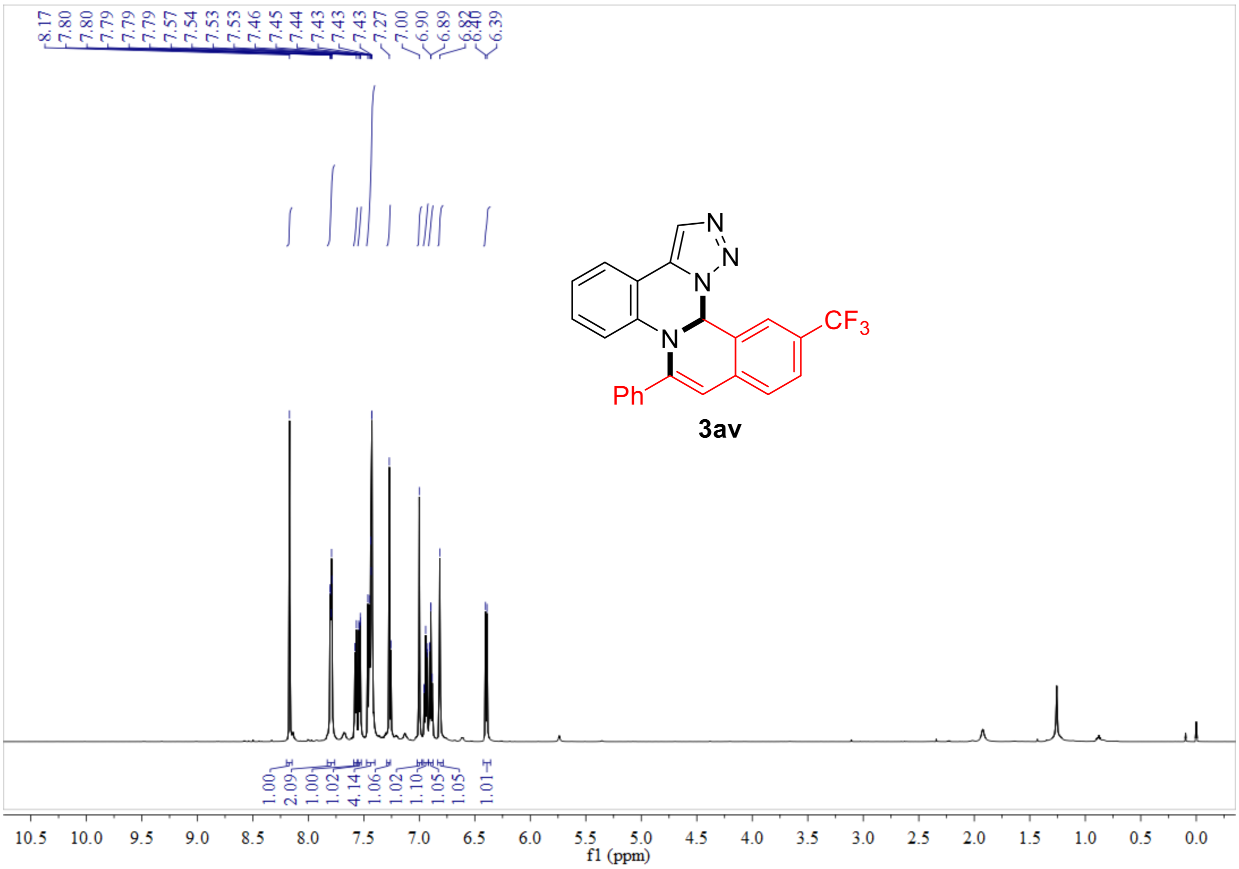

8.17
7.80
7.80
7.79
7.79
7.79
7.57
7.54
7.53
7.53
7.46
7.45
7.44
7.43
7.43
7.43
7.27
7.00
6.90
6.89
6.82
6.40
6.39
CF3
Ph
N
3av
1.00
2.09
1.00
1.02
4.14
1.06
1.02
1.10
1.05
1.05
1.01
10.5 10.0 9.5 9.0 8.5 8.0 7.5 7.0 6.5 6.0 5.5 5.0 4.5 4.0 3.5 3.0 2.5 2.0 1.5 1.0 0.5 0.0
f1 (ppm)

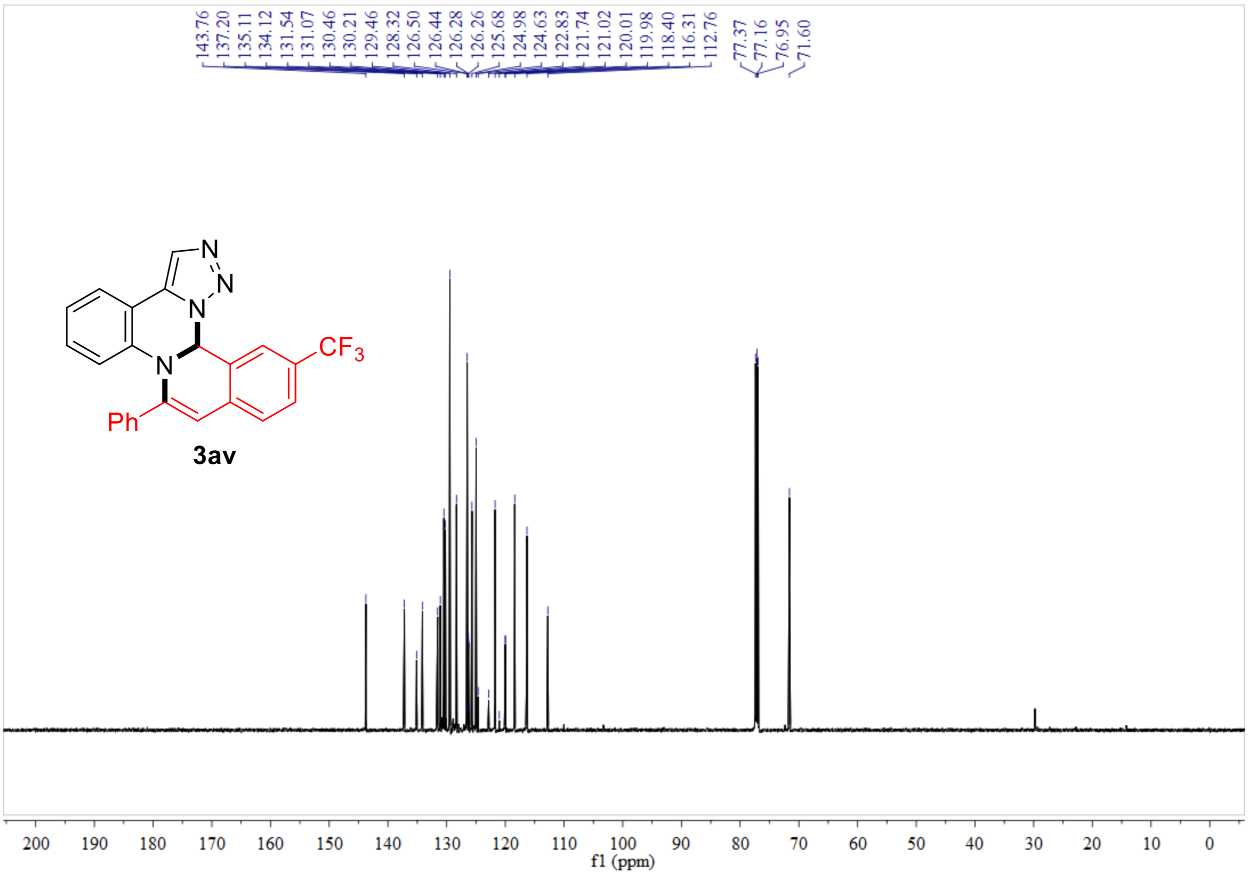

143.76
137.20
135.11
134.12
131.54
131.07
130.46
130.21
129.46
128.32
126.50
126.44
126.28
126.26
125.68
124.98
124.63
122.83
121.74
121.02
120.01
119.98
118.40
116.31
112.76
77.37
77.16
76.95
71.60
CF3
Ph
N
3av
200 190 180 170 160 150 140 130 120 110 100 90 80 70 60 50 40 30 20 10 0
f1 (ppm)

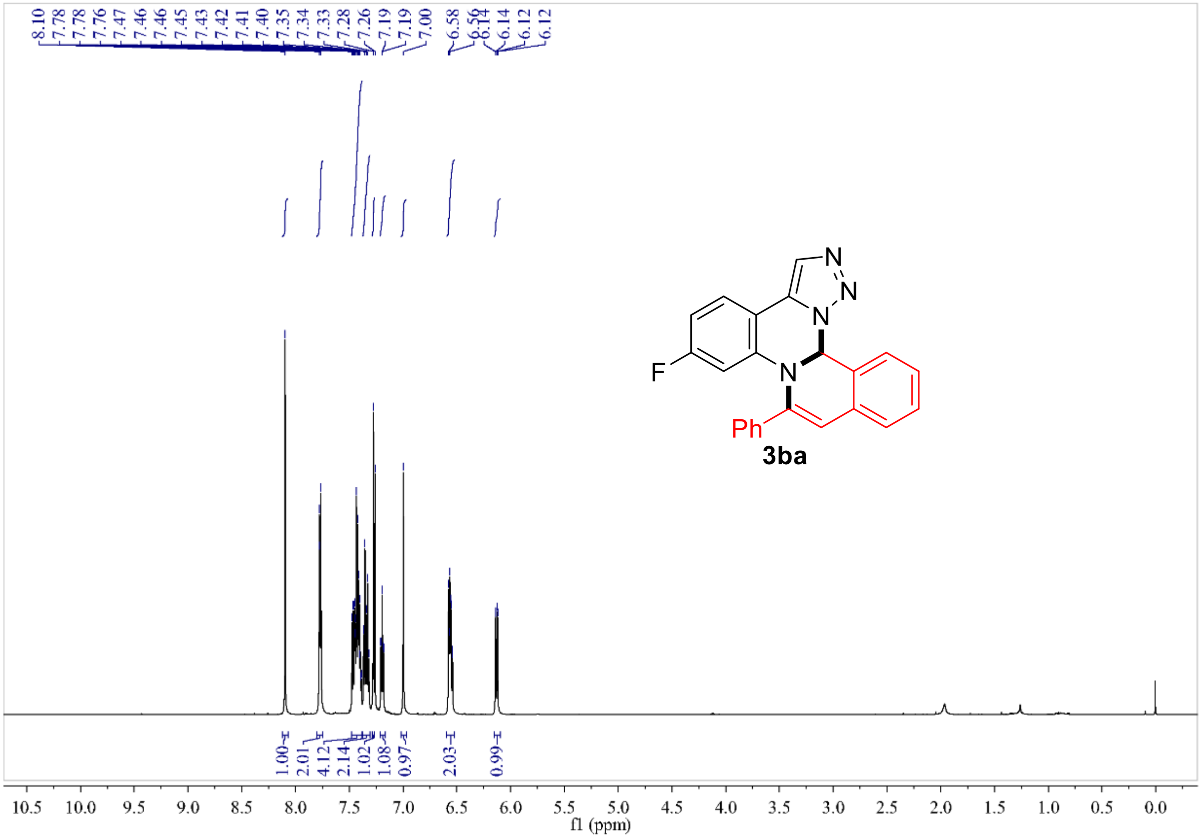
8.10
7.78
7.78
7.76
7.47
7.46
7.46
7.45
7.43
7.42
7.41
7.40
7.35
7.34
7.33
7.28
7.26
7.19
7.19
7.00
6.58
6.56
6.14
6.14
6.12
6.12
F
N
N
N
N
Ph
3ba
1.00
2.01
4.12
2.14
1.02
1.08
0.97
2.03
0.99
f1 (ppm)

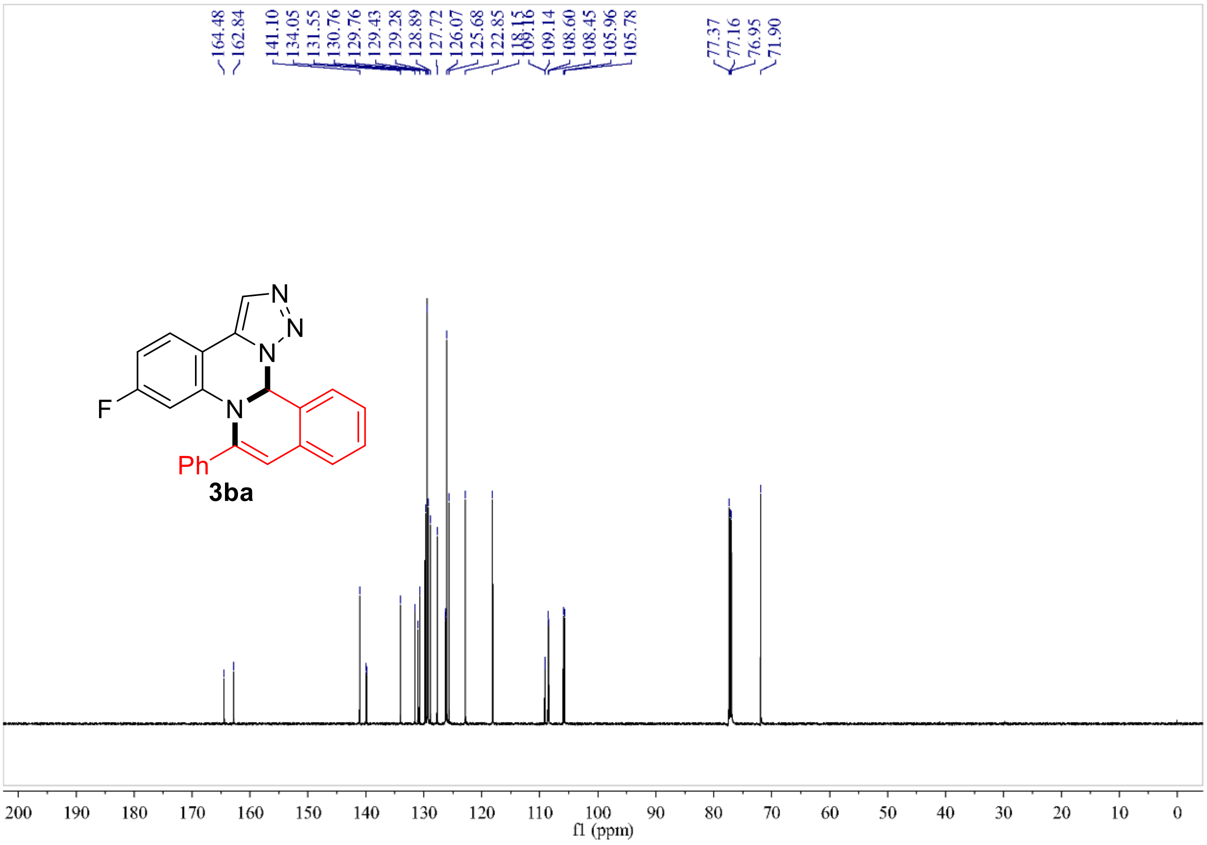
164.48
162.84
141.10
134.05
131.55
130.76
129.76
129.43
129.28
128.89
127.72
126.07
125.68
122.85
118.15
118.16
109.14
108.60
108.45
105.96
105.78
77.37
77.16
76.95
71.90
F
N
N
N
N
Ph
3ba
f1 (ppm)

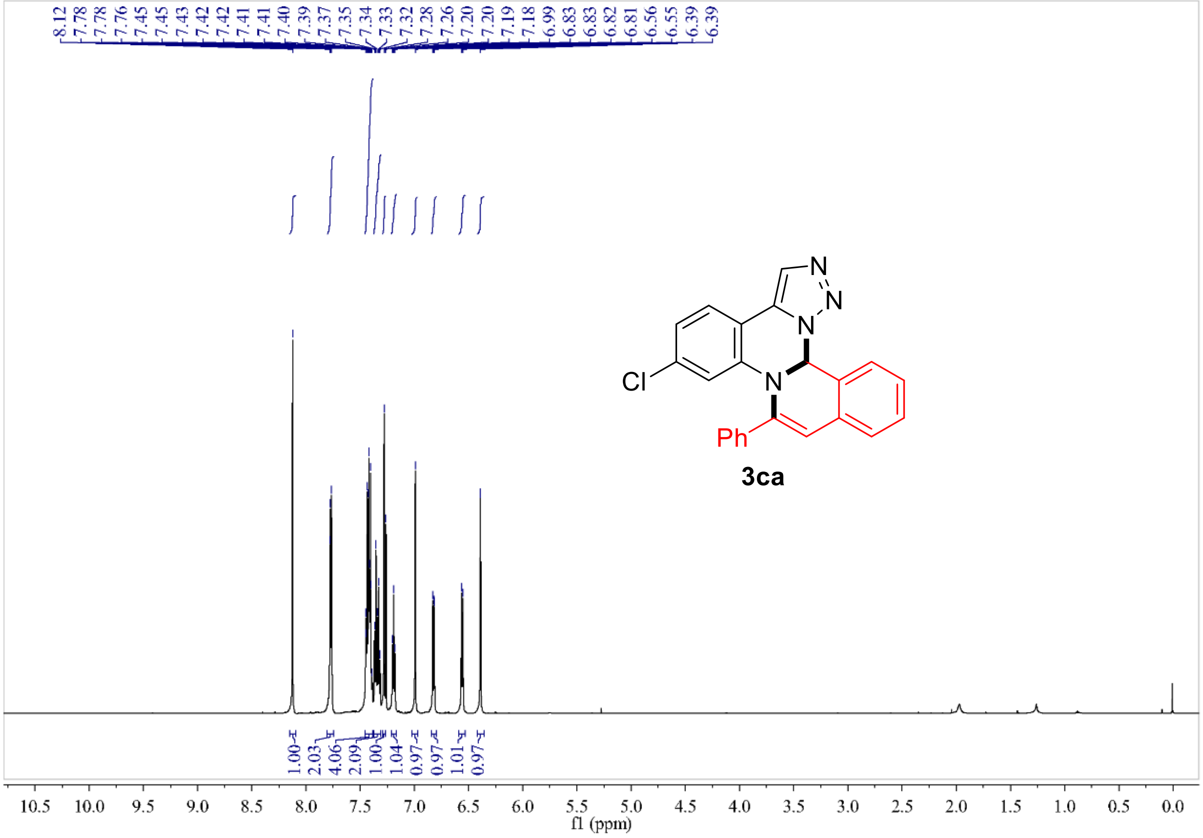

8.12
7.78
7.78
7.76
7.45
7.45
7.43
7.42
7.42
7.41
7.41
7.40
7.39
7.37
7.35
7.34
7.33
7.32
7.28
7.26
7.20
7.20
7.19
7.18
6.99
6.83
6.83
6.82
6.81
6.56
6.55
6.39
6.39
Cl
N
N
N
N
Ph
3ca
1.00
2.03
4.06
2.09
1.00
1.04
0.97
0.97
1.01
0.97
10.5
10.0
9.5
9.0
8.5
8.0
7.5
7.0
6.5
6.0
5.5
5.0
4.5
4.0
3.5
3.0
2.5
2.0
1.5
1.0
0.5
0.0
f1 (ppm)

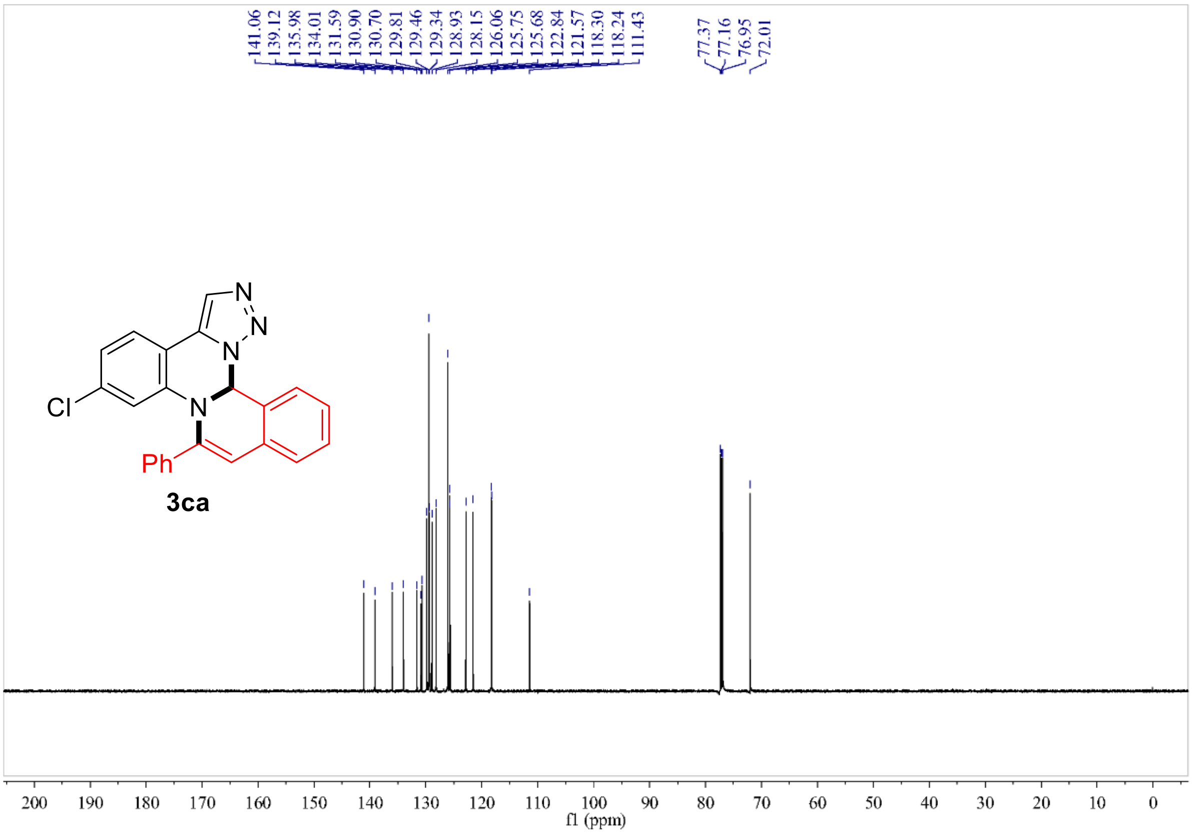

141.06
139.12
135.98
134.01
131.59
130.90
130.70
129.81
129.46
129.34
128.93
128.15
126.06
125.75
125.68
122.84
121.57
118.30
118.24
111.43
77.37
77.16
76.95
72.01
Cl
N
N
N
N
Ph
3ca
200
190
180
170
160
150
140
130
120
110
100
90
80
70
60
50
40
30
20
10
0
f1 (ppm)

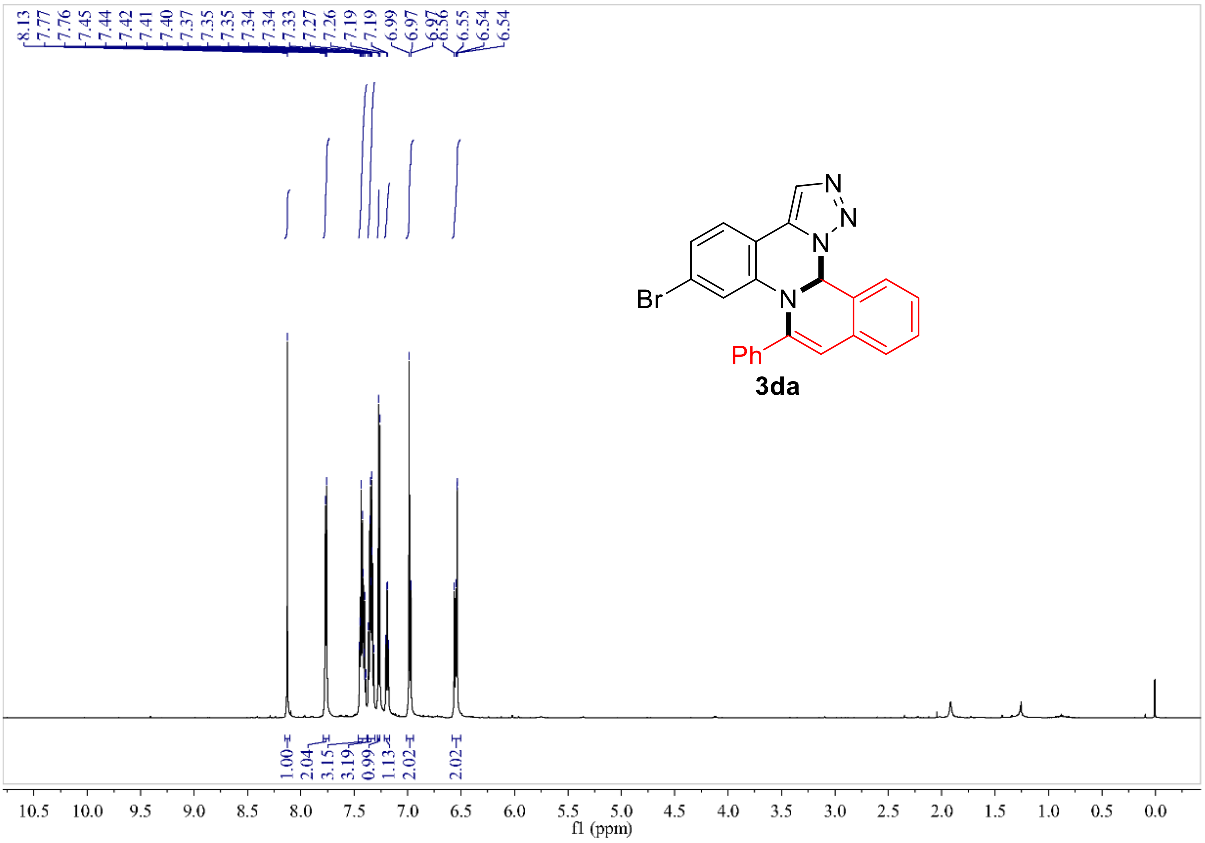
8.13
7.77
7.76
7.45
7.44
7.42
7.41
7.40
7.37
7.35
7.35
7.34
7.34
7.33
7.27
7.26
7.19
7.19
6.99
6.97
6.97
6.56
6.55
6.54
6.54
Br
N
N
N
N
Ph
3da
1.00
2.04
3.15
3.19
0.99
1.13
2.02
2.02
f1 (ppm)

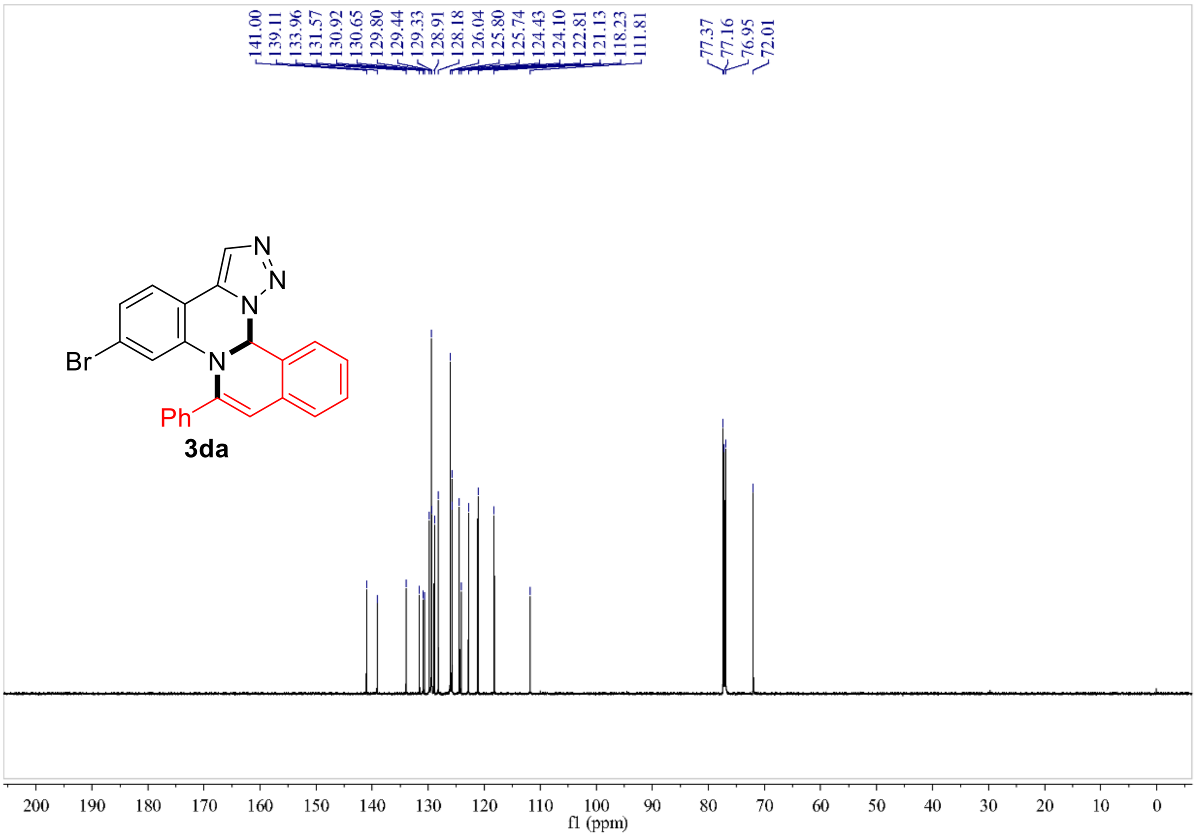
141.00
139.11
133.96
131.57
130.92
130.65
129.80
129.44
129.33
128.91
128.18
126.04
125.80
125.74
124.43
124.10
122.81
121.13
118.23
111.81
77.37
77.16
76.95
72.01
Br
N
N
N
N
Ph
3da
f1 (ppm)

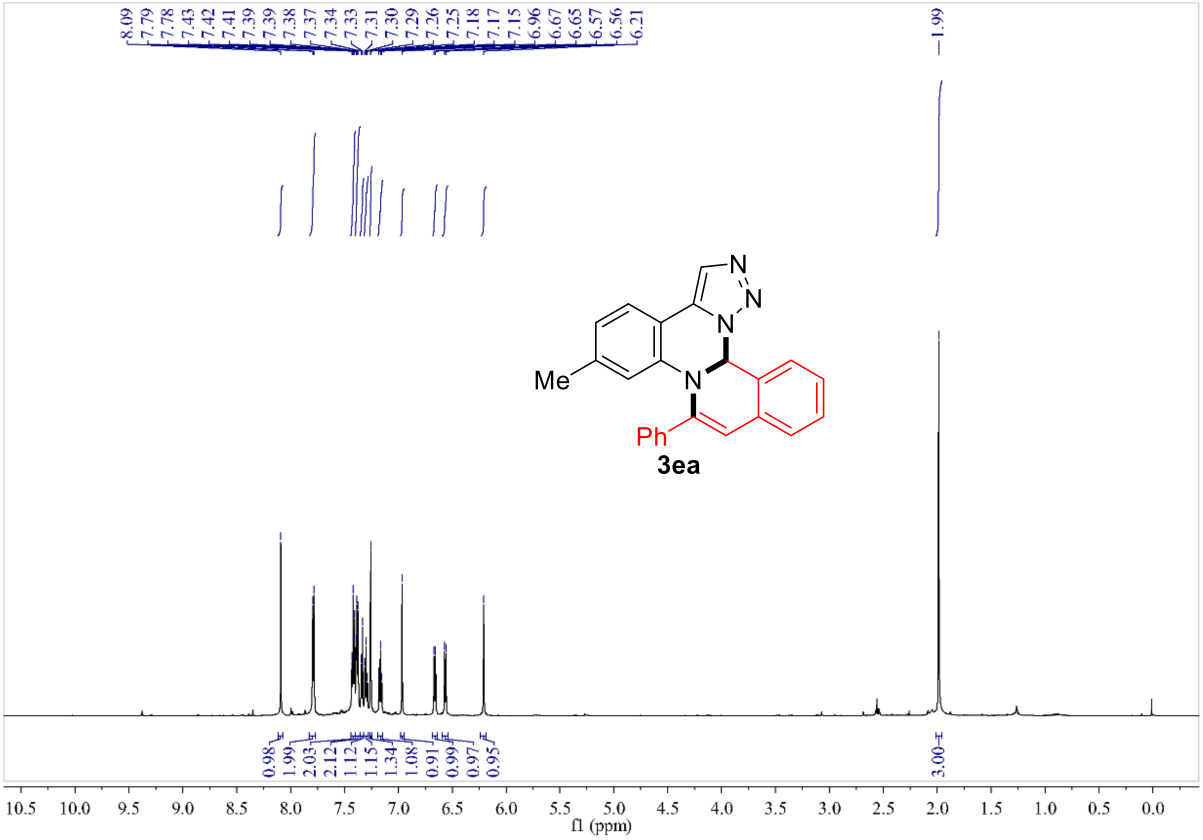
8.09
7.79
7.78
7.43
7.42
7.41
7.39
7.39
7.38
7.37
7.34
7.33
7.31
7.30
7.29
7.26
7.25
7.18
7.17
7.15
6.96
6.67
6.65
6.57
6.56
6.21
1.99
Me
Ph
3ea
0.98
1.99
2.03
2.12
1.12
1.15
1.34
1.08
0.91
0.99
0.97
0.95
3.00
f1 (ppm)

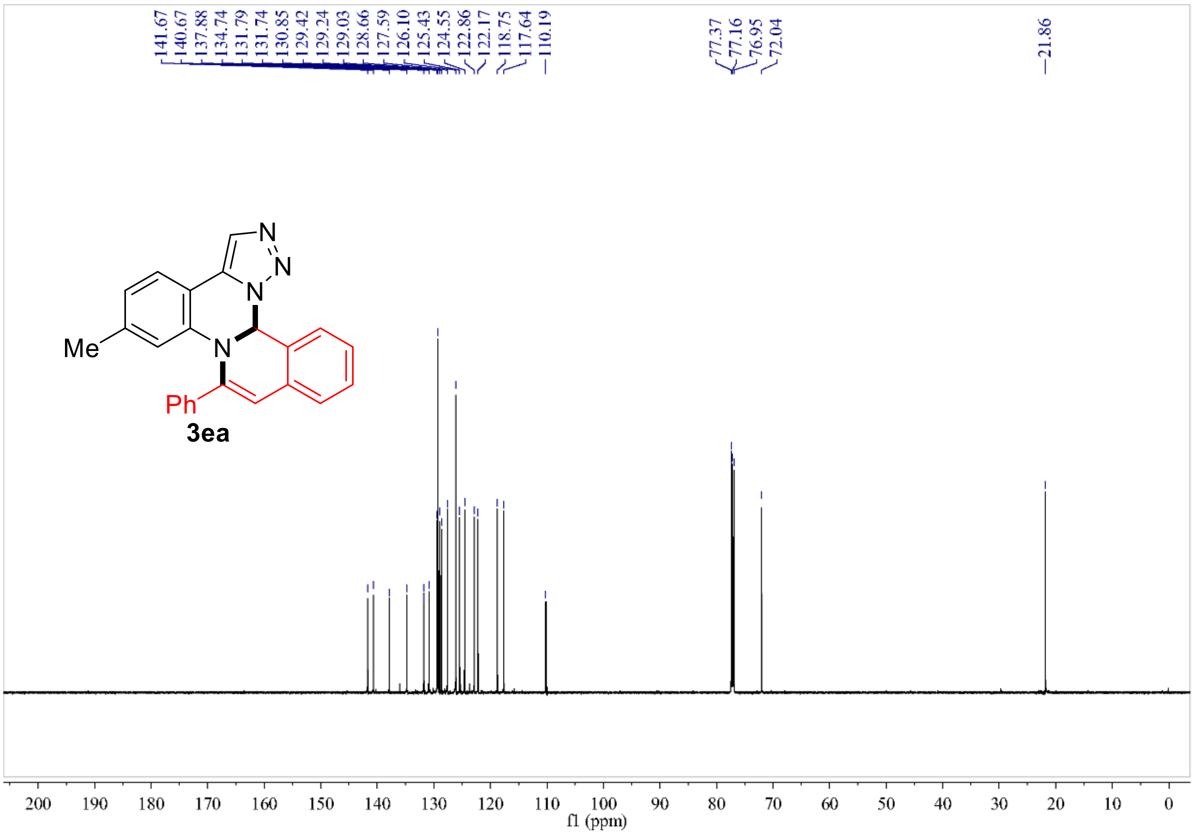
141.67
140.67
137.88
134.74
131.79
131.74
130.85
129.42
129.24
129.03
128.66
127.59
126.10
125.43
124.55
122.86
122.17
118.75
117.64
110.19
77.37
77.16
76.95
72.04
21.86
Me
Ph
3ea
f1 (ppm)

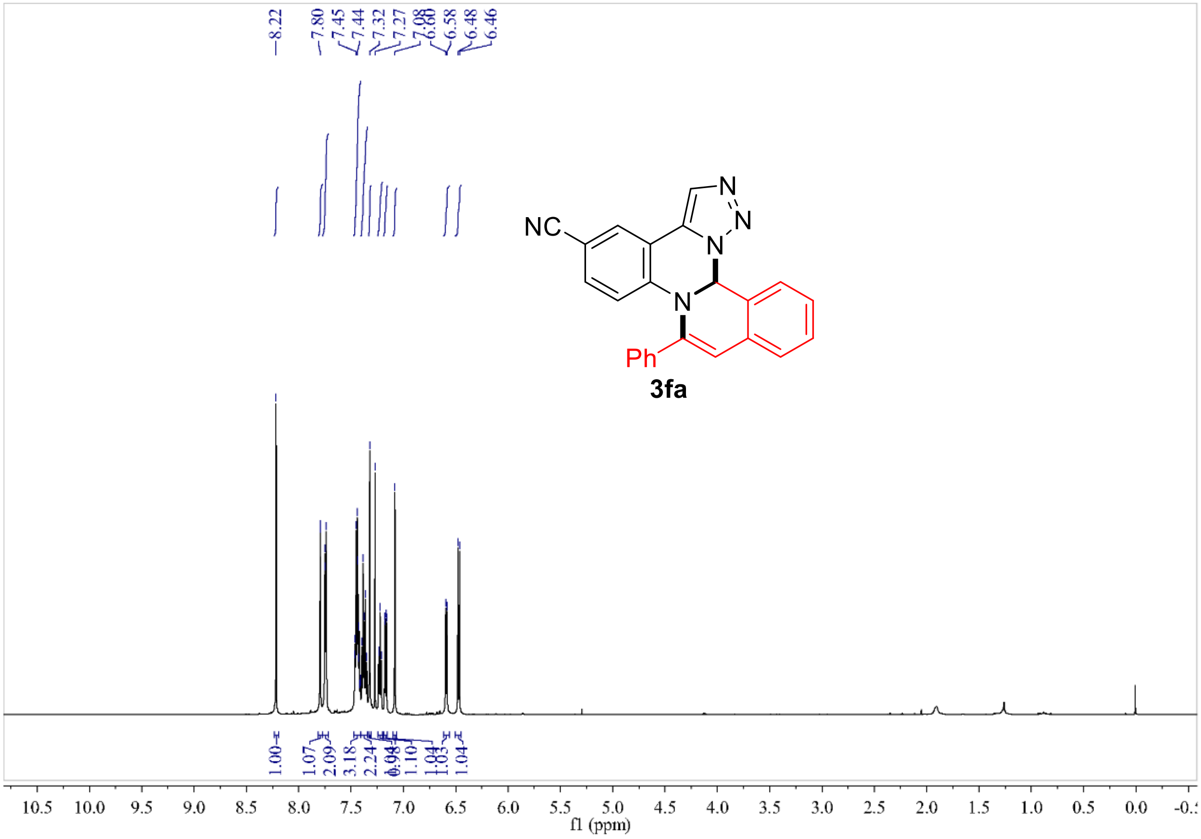
8.22
7.80
7.45
7.44
7.32
7.27
7.08
6.58
6.48
6.46
NC
N
N
N
N
Ph
3fa
1.00
1.07
2.09
3.18
2.24
1.10
1.04
1.03
1.04
10.5
10.0
9.5
9.0
8.5
8.0
7.5
7.0
6.5
6.0
5.5
5.0
4.5
4.0
3.5
3.0
2.5
2.0
1.5
1.0
0.5
0.0
f1 (ppm)

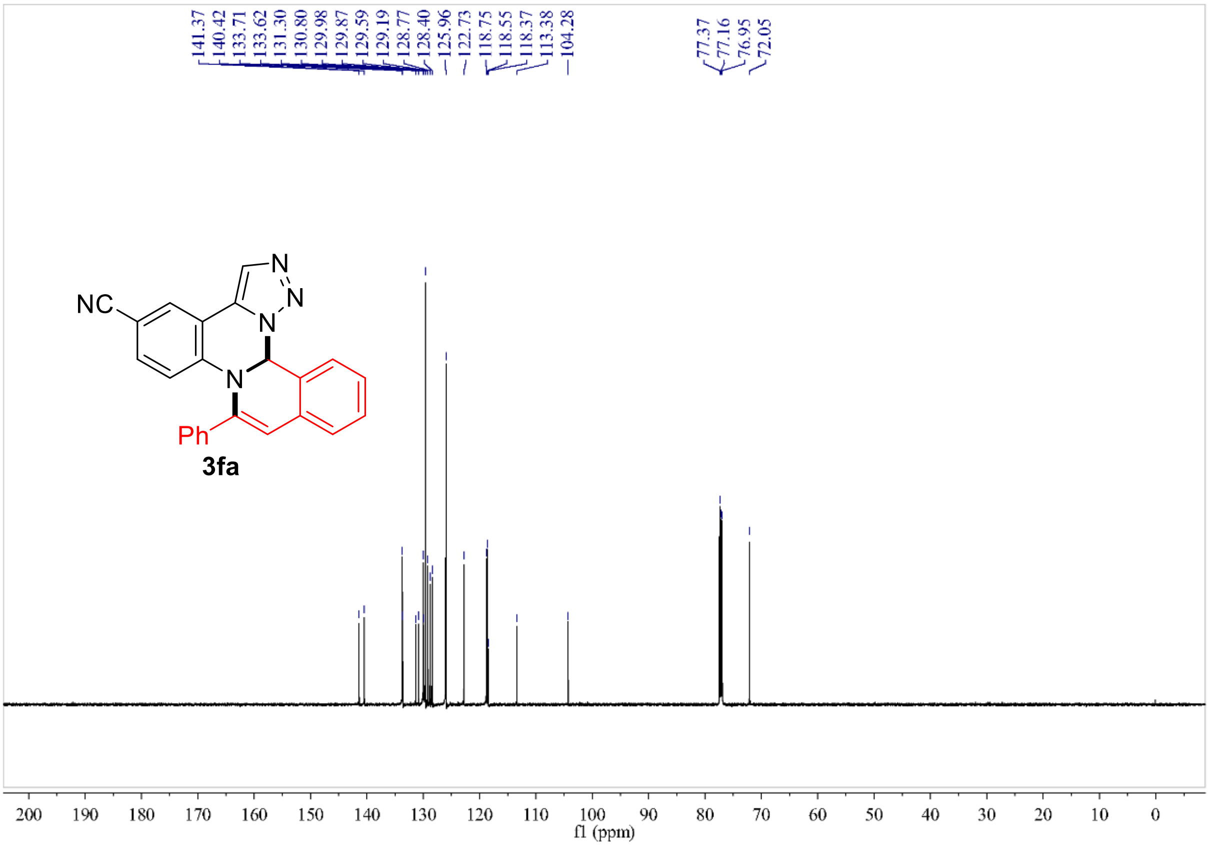
141.37
140.42
133.71
133.62
131.30
130.80
129.98
129.87
129.59
129.19
128.77
128.40
125.96
122.73
118.75
118.55
118.37
113.38
104.28
77.37
77.16
76.95
72.05
NC
N
N
N
N
Ph
3fa
200
190
180
170
160
150
140
130
120
110
100
90
80
70
60
50
40
30
20
10
0
f1 (ppm)

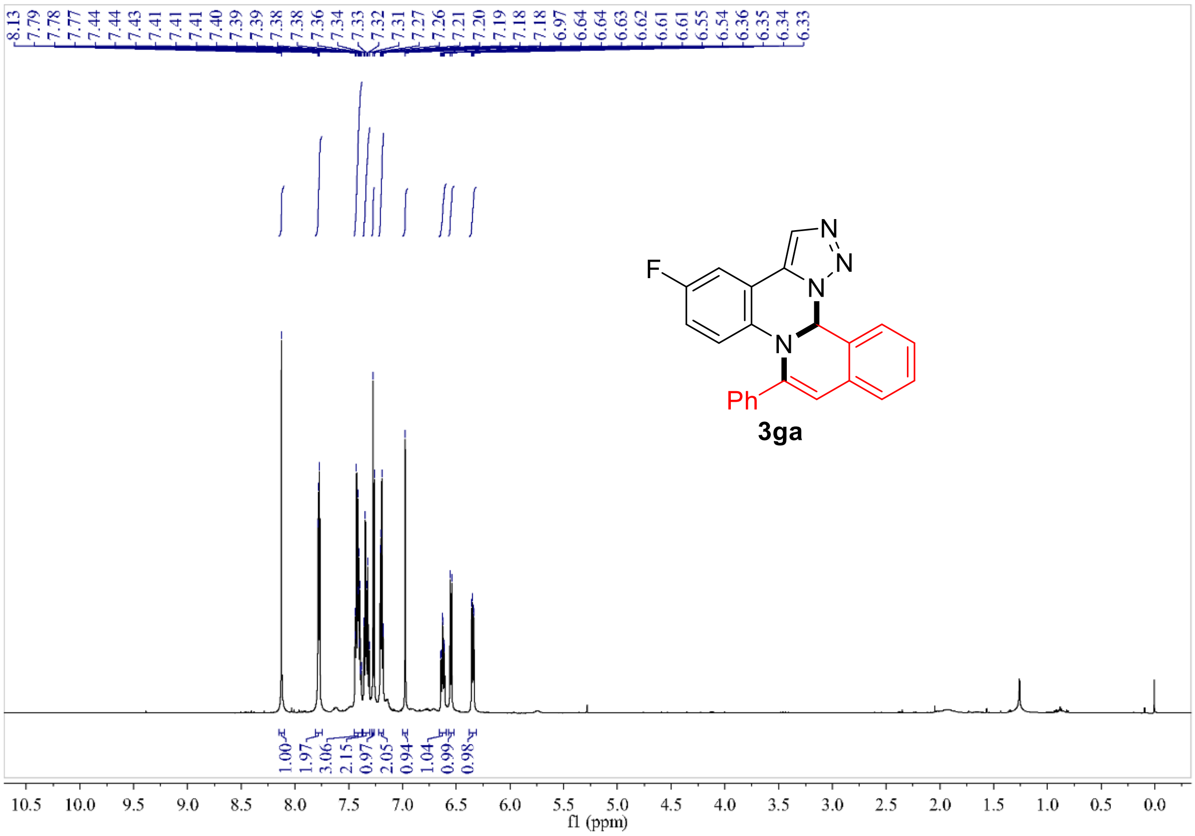

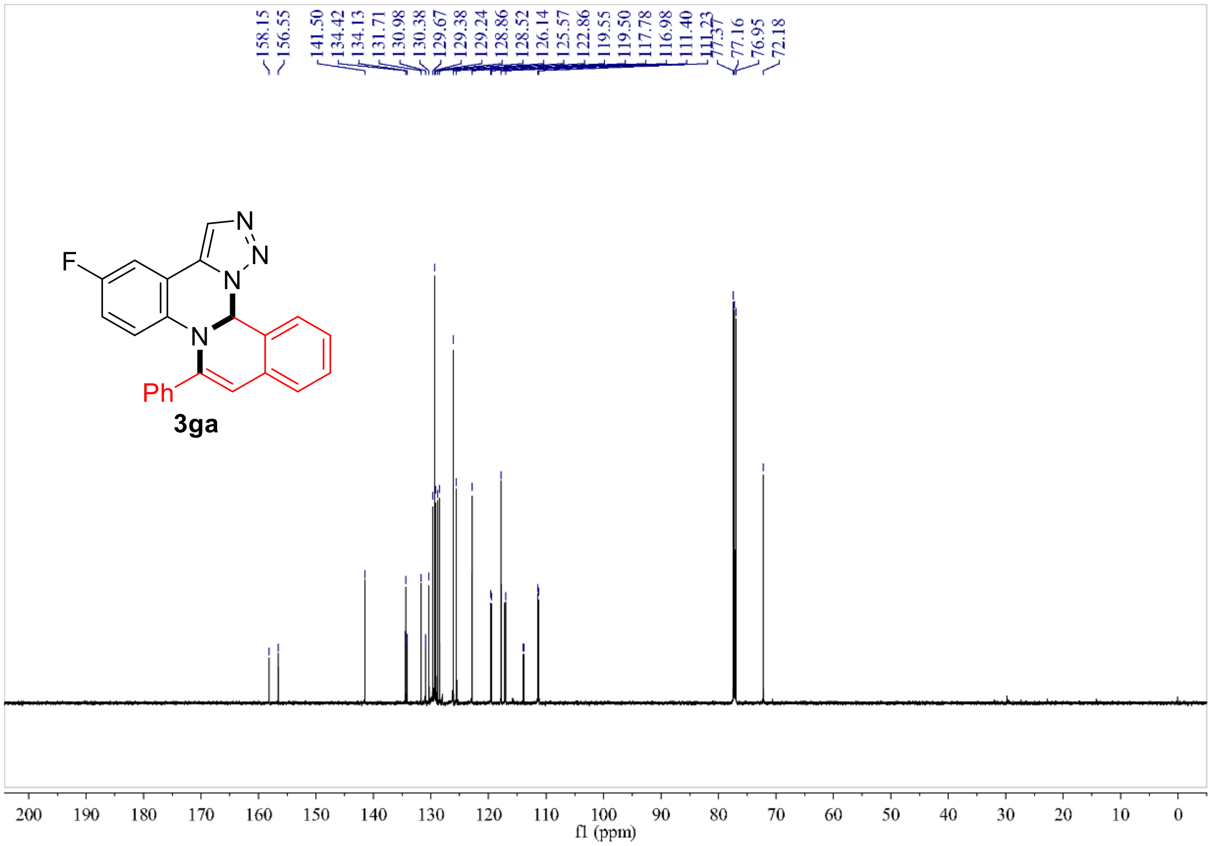

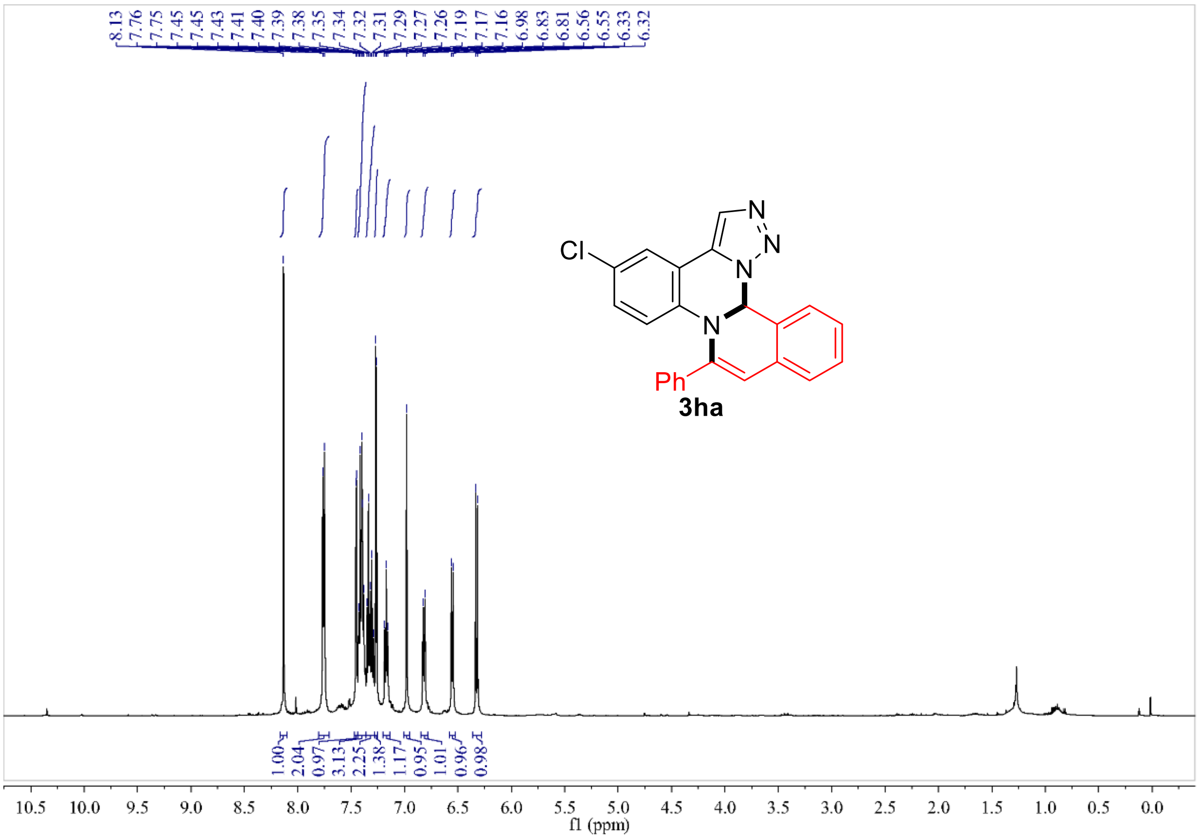
8.13 7.76 7.75 7.45 7.45 7.43 7.41 7.40 7.39 7.38 7.35 7.34 7.32 7.31 7.29 7.27 7.26 7.19 7.17 7.16 6.98 6.83 6.81 6.56 6.55 6.33 6.32
Cl
N
N
N
N
Ph
3ha
1.00 2.04 0.97 3.13 2.25 1.38 1.17 0.95 1.01 0.96 0.98
10.5 10.0 9.5 9.0 8.5 8.0 7.5 7.0 6.5 6.0 5.5 5.0 4.5 4.0 3.5 3.0 2.5 2.0 1.5 1.0 0.5 0.0
f1 (ppm)

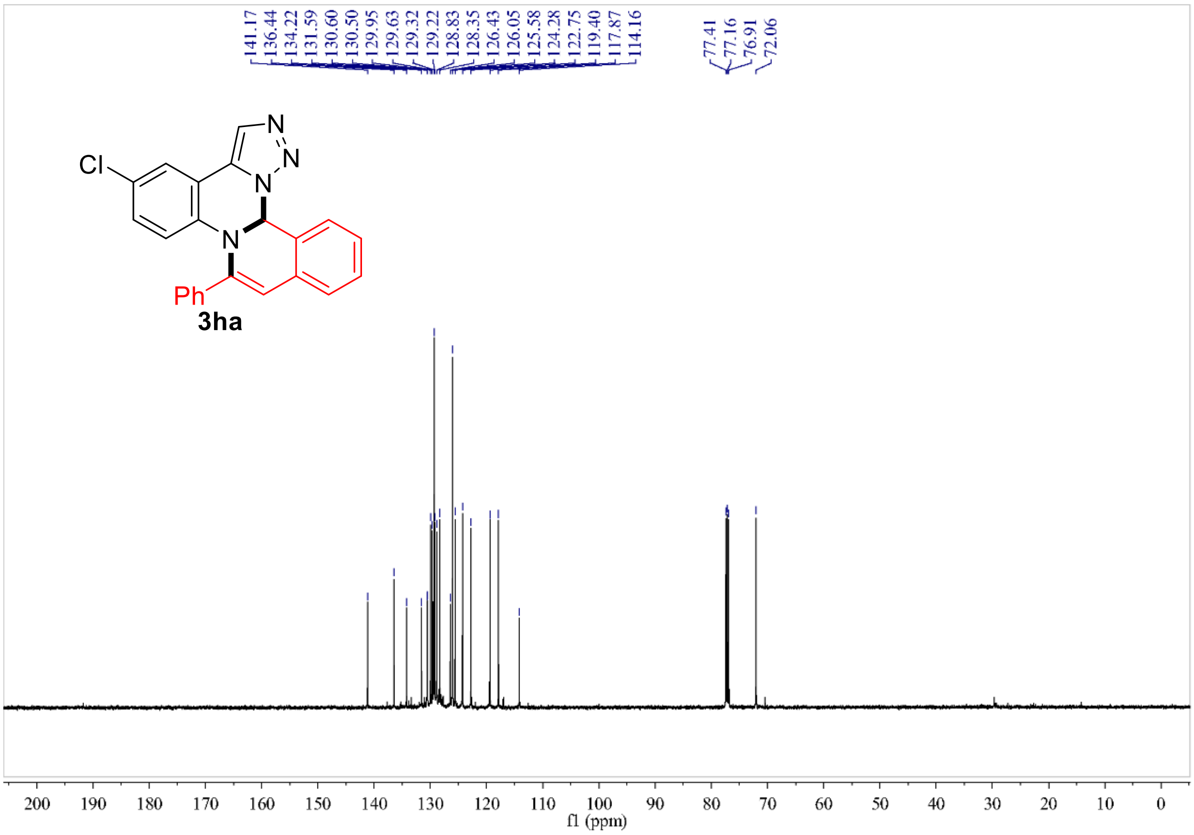
141.17 136.44 134.22 131.59 130.60 130.50 129.95 129.63 129.32 129.22 128.83 128.35 126.43 126.05 125.58 124.28 122.75 119.40 117.87 114.16
77.41 77.16 76.91 72.06
Cl
N
N
N
N
Ph
3ha
200 190 180 170 160 150 140 130 120 110 100 90 80 70 60 50 40 30 20 10 0
f1 (ppm)

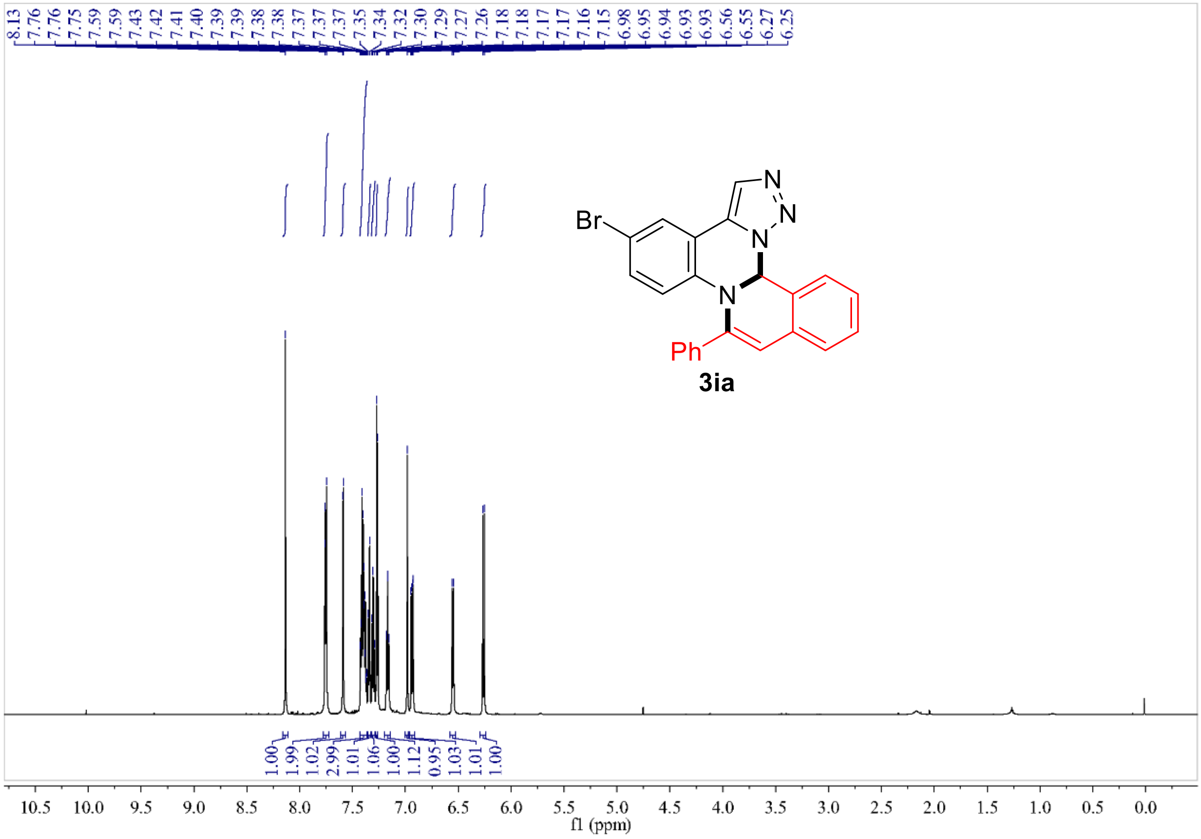
8.13
7.76
7.76
7.75
7.59
7.59
7.43
7.42
7.41
7.40
7.39
7.39
7.38
7.38
7.37
7.37
7.37
7.35
7.34
7.32
7.30
7.29
7.27
7.26
7.18
7.18
7.17
7.17
7.16
7.15
6.98
6.95
6.94
6.93
6.93
6.56
6.55
6.27
6.25
Br
N
N
N
N
Ph
3ia
1.00
1.99
1.02
2.99
1.01
1.06
1.00
1.12
0.95
1.03
1.01
1.00
f1 (ppm)

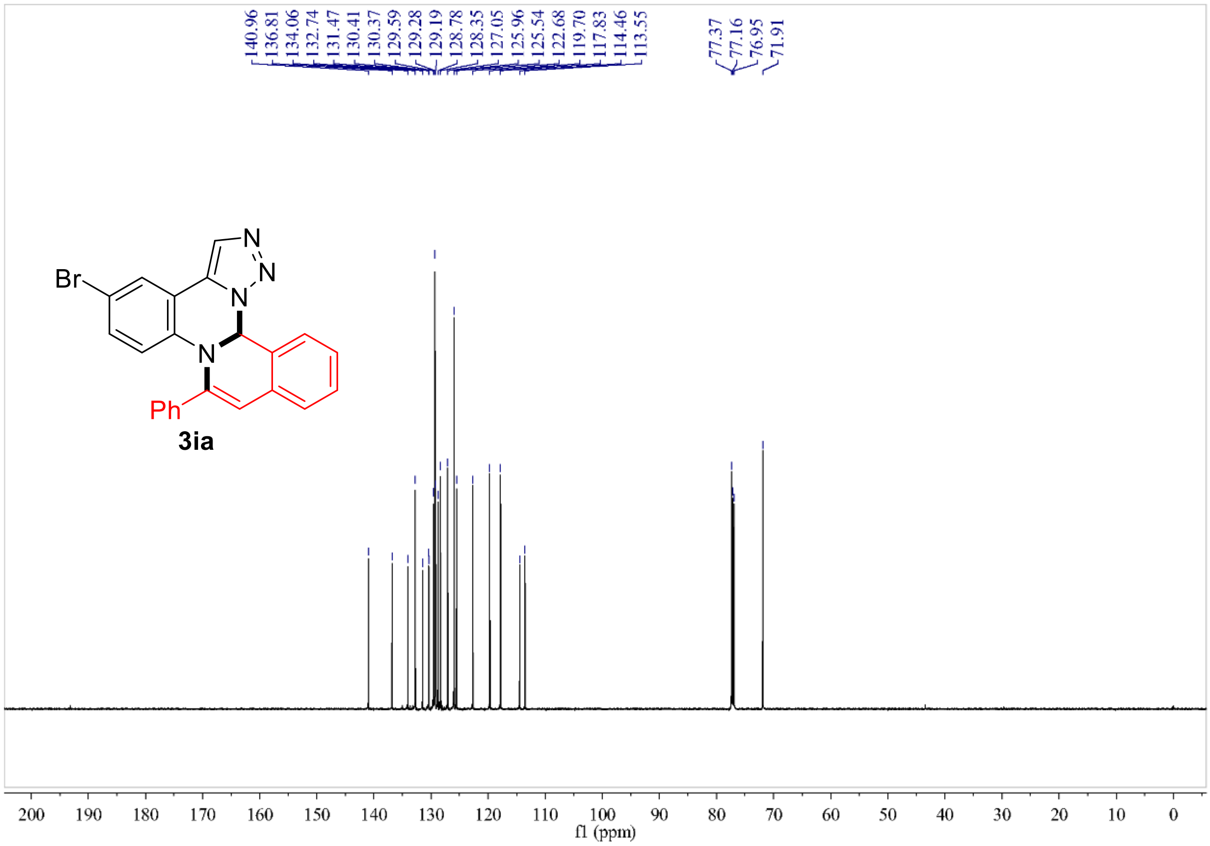
140.96
136.81
134.06
132.74
131.47
130.41
130.37
129.59
129.28
129.19
128.78
128.35
127.05
125.96
125.54
122.68
119.70
117.83
114.46
113.55
77.37
77.16
76.95
71.91
Br
N
N
N
N
Ph
3ia
f1 (ppm)

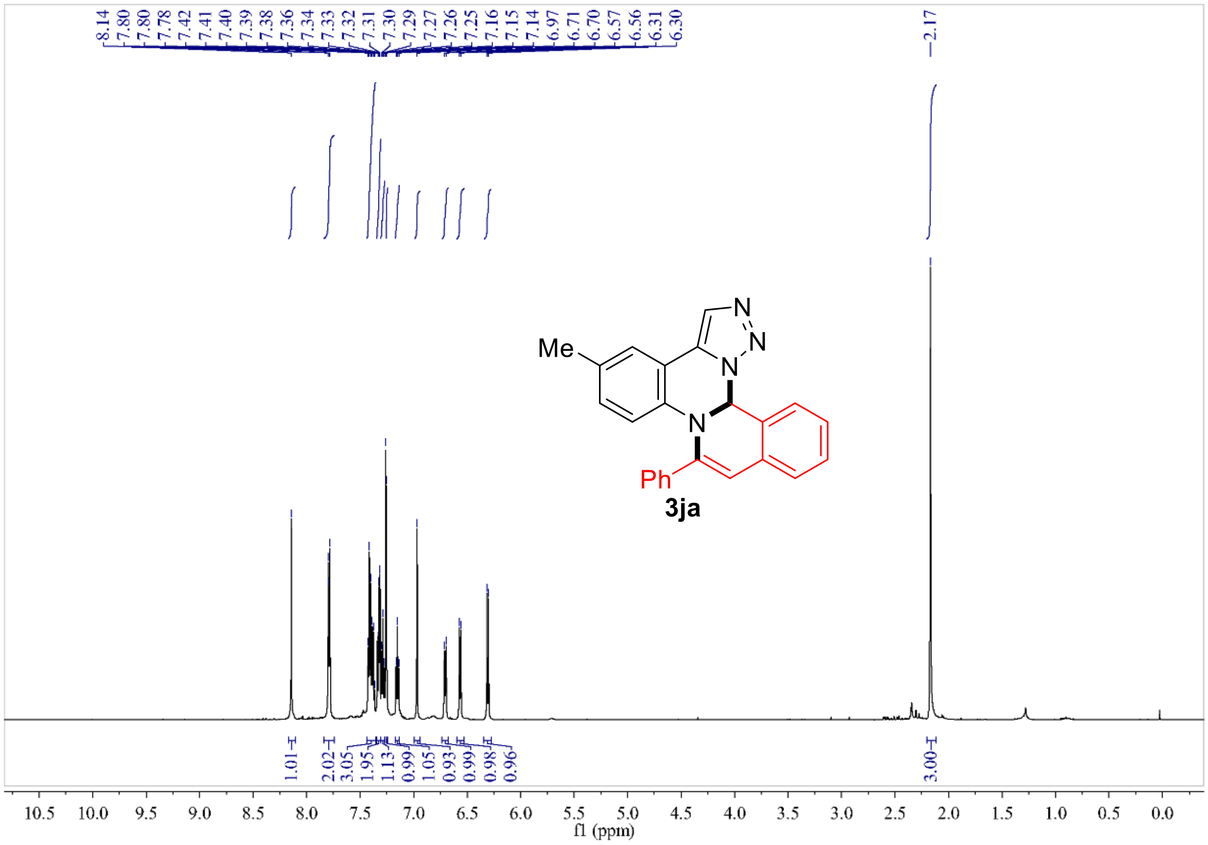
8.14 7.80 7.80 7.78 7.42 7.41 7.40 7.39 7.38 7.36 7.34 7.33 7.32 7.31 7.30 7.29 7.27 7.26 7.25 7.16 7.15 7.14 6.97 6.71 6.70 6.57 6.56 6.31 6.30
2.17
Me
N
N
N
N
Ph
3ja
1.01 2.02 3.05 1.95 1.13 0.99 1.05 0.93 0.99 0.99 0.98 0.96
3.00
10.5 10.0 9.5 9.0 8.5 8.0 7.5 7.0 6.5 6.0 5.5 5.0 4.5 4.0 3.5 3.0 2.5 2.0 1.5 1.0 0.5 0.0
f1 (ppm)

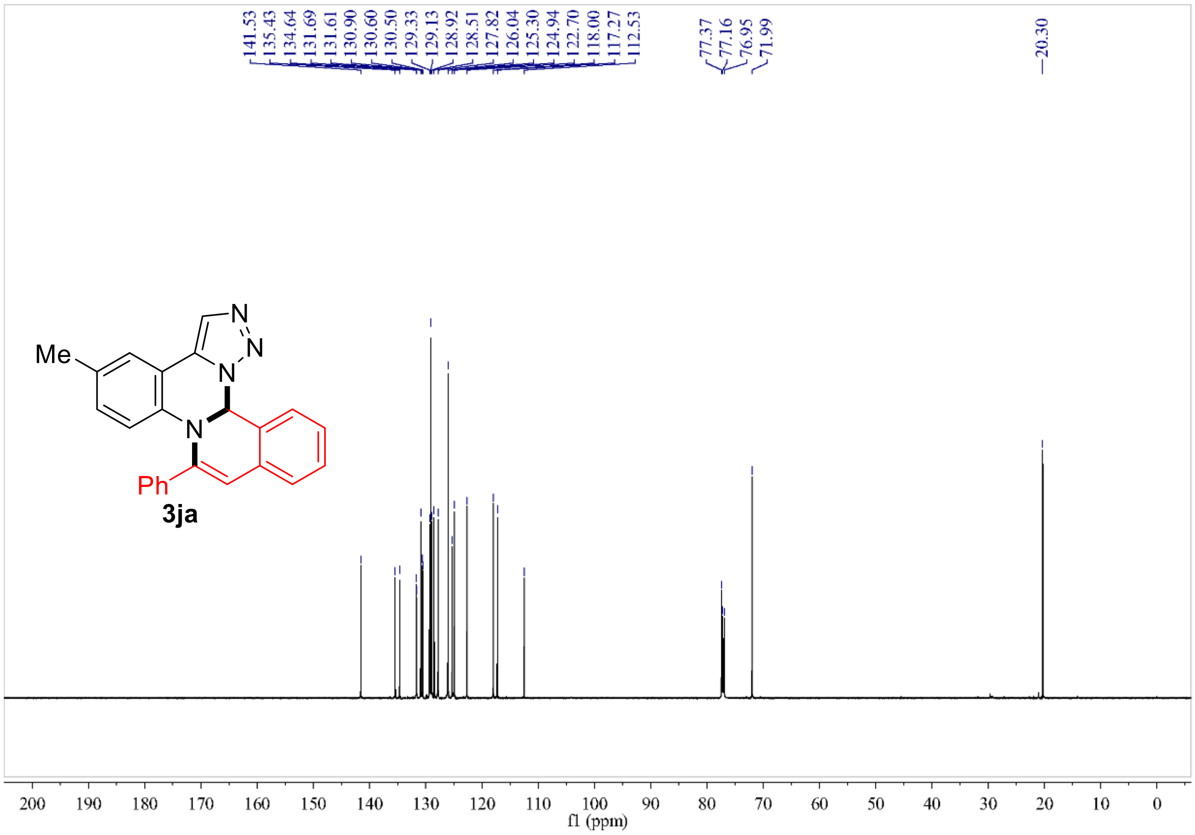
141.53 135.43 134.64 131.69 131.61 130.90 130.60 130.50 129.33 129.13 128.92 128.51 127.82 126.04 125.30 124.94 122.70 118.00 117.27 112.53
77.37 77.16 76.95 71.99
20.30
Me
N
N
N
N
Ph
3ja
200 190 180 170 160 150 140 130 120 110 100 90 80 70 60 50 40 30 20 10 0
f1 (ppm)

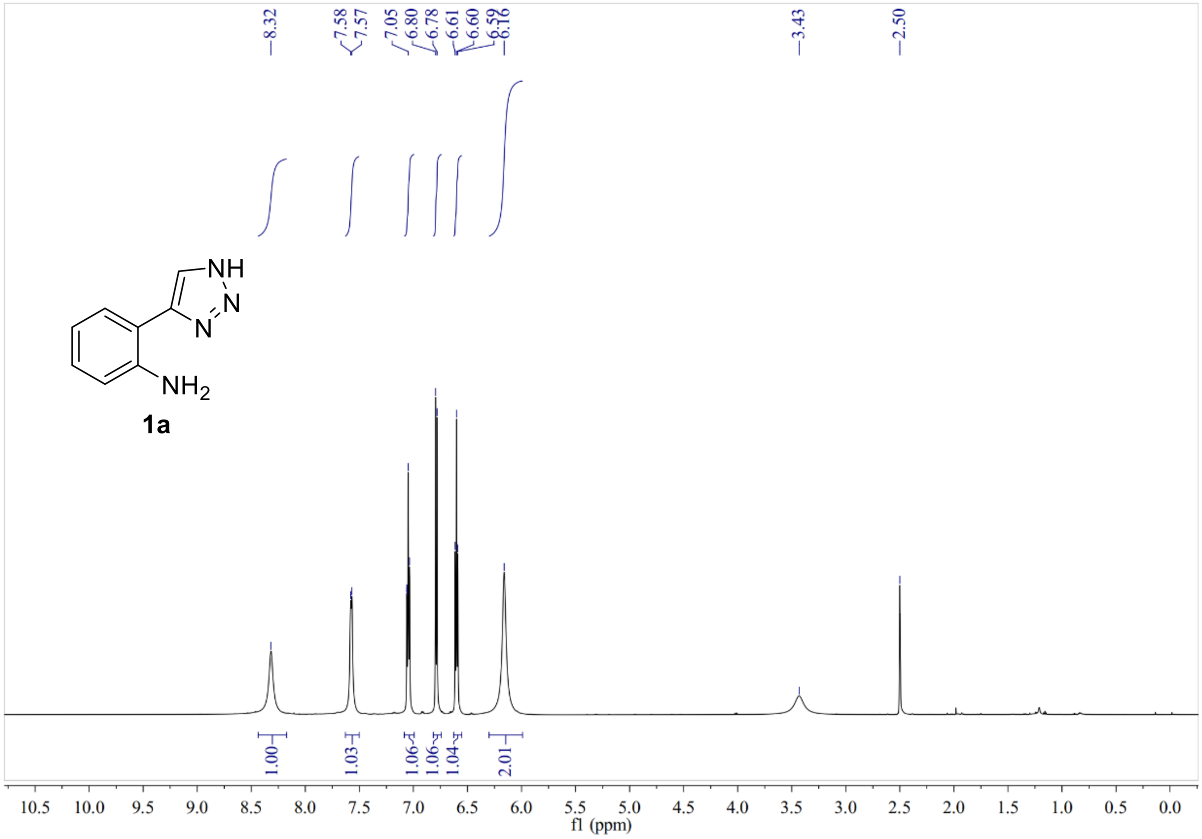
8.32
7.58
7.57
7.05
6.80
6.78
6.61
6.60
6.59
6.16
3.43
2.50
NH
N
N
NH2
1a
1.00
1.03
1.06
1.06
1.04
2.01
10.5 10.0 9.5 9.0 8.5 8.0 7.5 7.0 6.5 6.0 5.5 5.0 4.5 4.0 3.5 3.0 2.5 2.0 1.5 1.0 0.5 0.0
f1 (ppm)

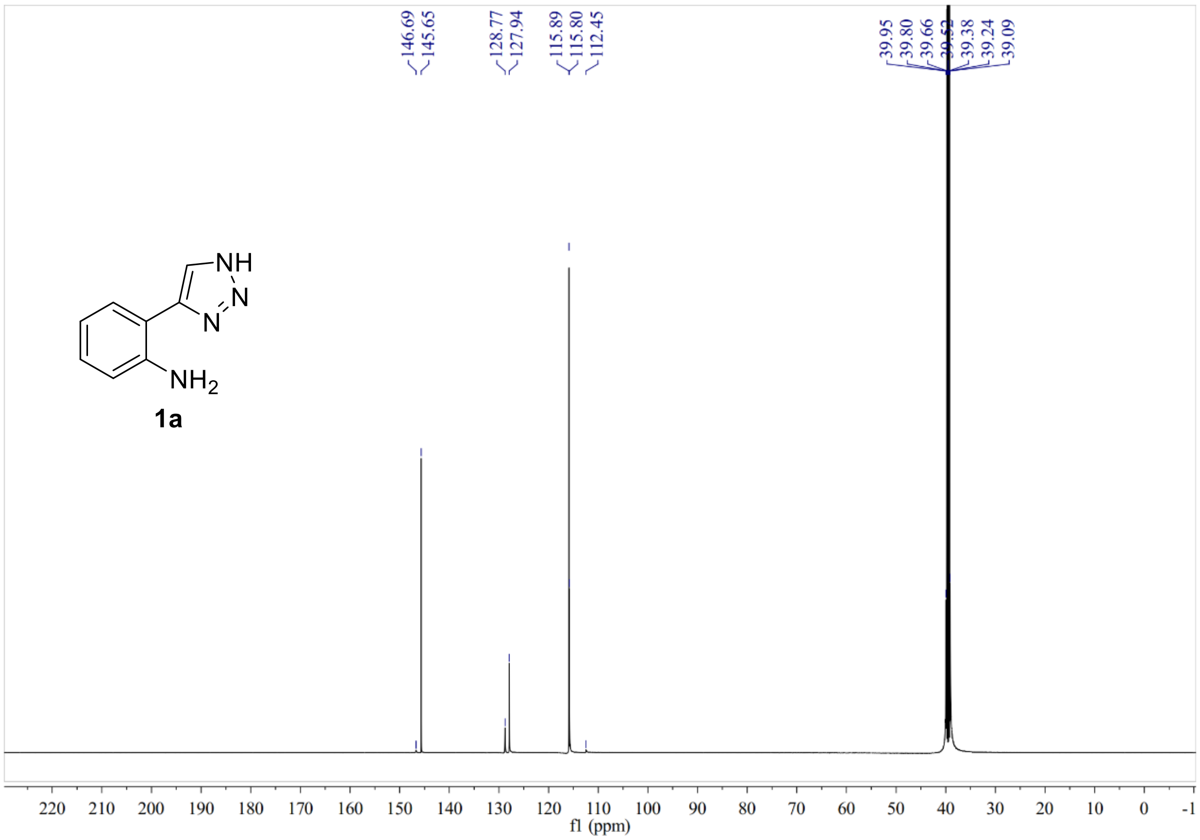
146.69
145.65
128.77
127.94
115.89
115.80
112.45
39.95
39.80
39.66
39.52
39.38
39.24
39.09
NH
N
N
NH2
1a
220 210 200 190 180 170 160 150 140 130 120 110 100 90 80 70 60 50 40 30 20 10 0 -1
f1 (ppm)

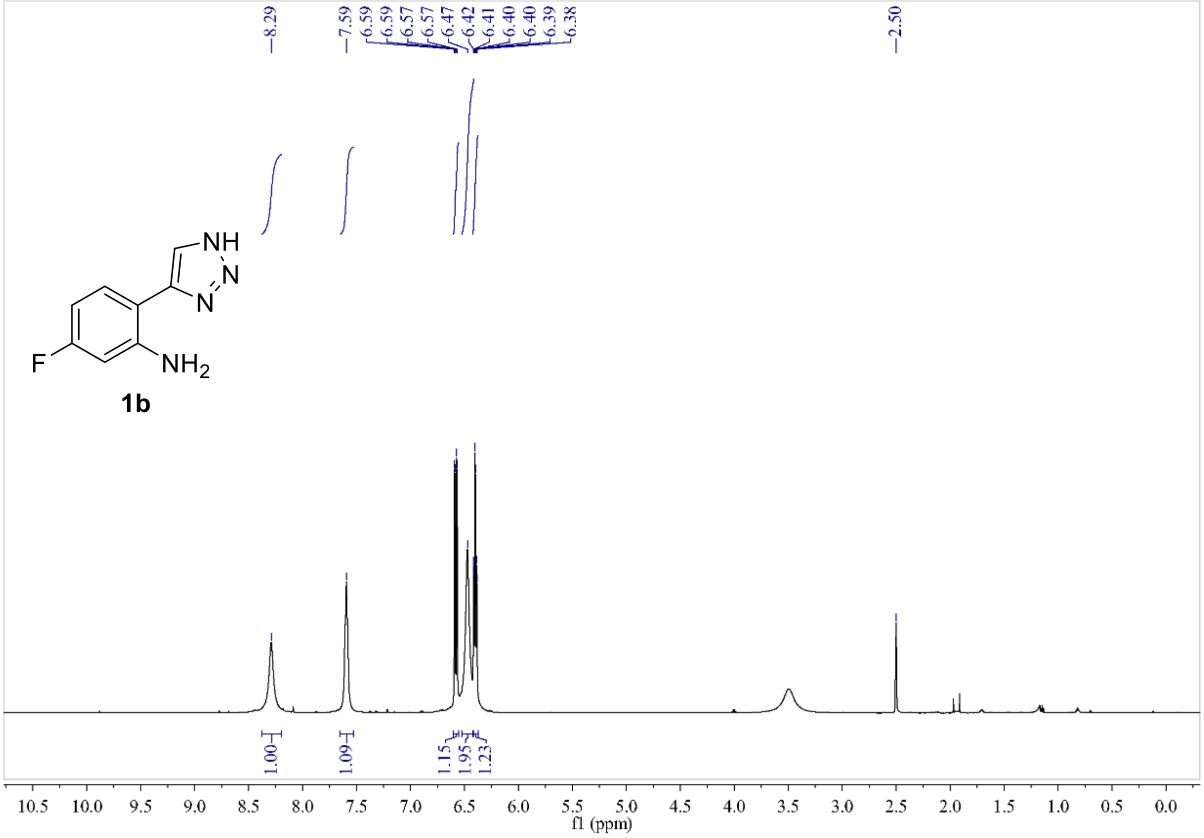
8.29
7.59
6.59
6.59
6.57
6.57
6.47
6.42
6.41
6.40
6.40
6.39
6.38
2.50
NH
N
N
F
NH2
1b
1.00
1.09
1.15
1.95
1.23
10.5 10.0 9.5 9.0 8.5 8.0 7.5 7.0 6.5 6.0 5.5 5.0 4.5 4.0 3.5 3.0 2.5 2.0 1.5 1.0 0.5 0.0
f1 (ppm)

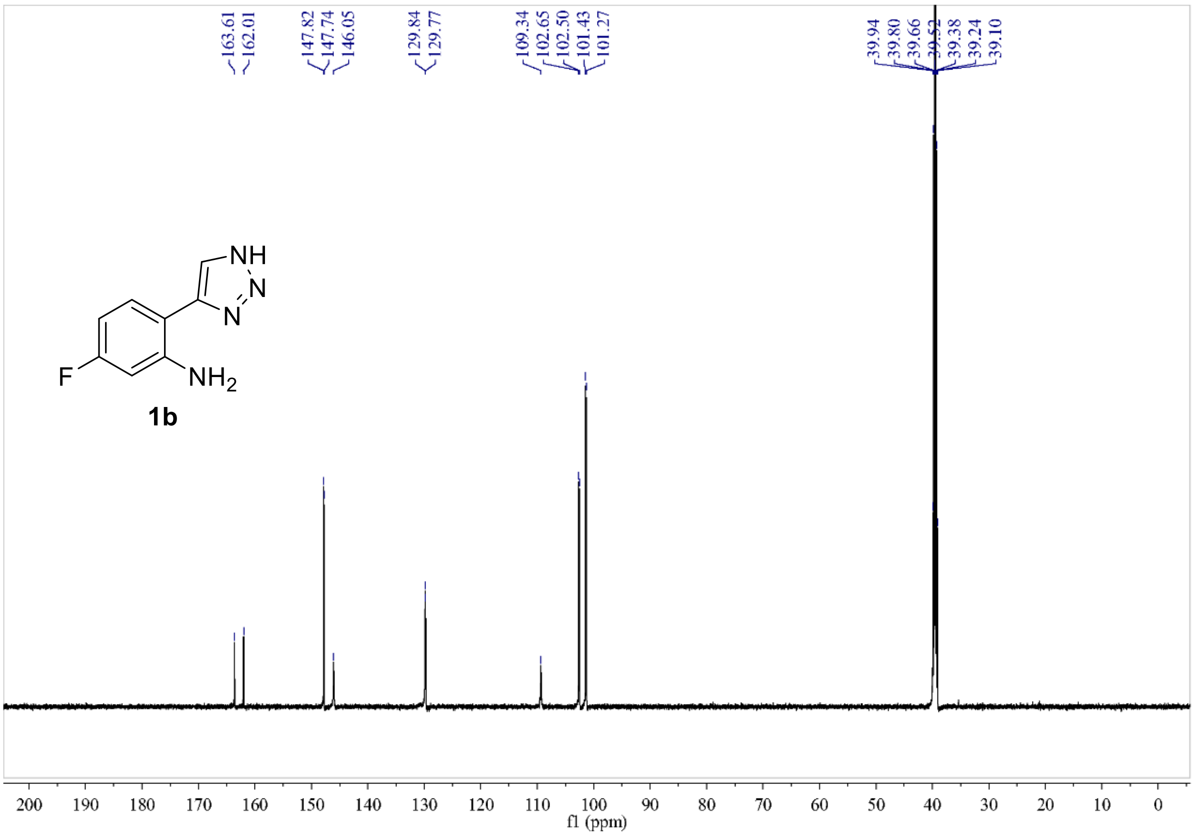
163.61
162.01
147.82
147.74
146.05
129.84
129.77
109.34
102.65
102.50
101.43
101.27
39.94
39.80
39.66
39.52
39.38
39.24
39.10
NH
N
N
F
NH2
1b
200 190 180 170 160 150 140 130 120 110 100 90 80 70 60 50 40 30 20 10 0
f1 (ppm)

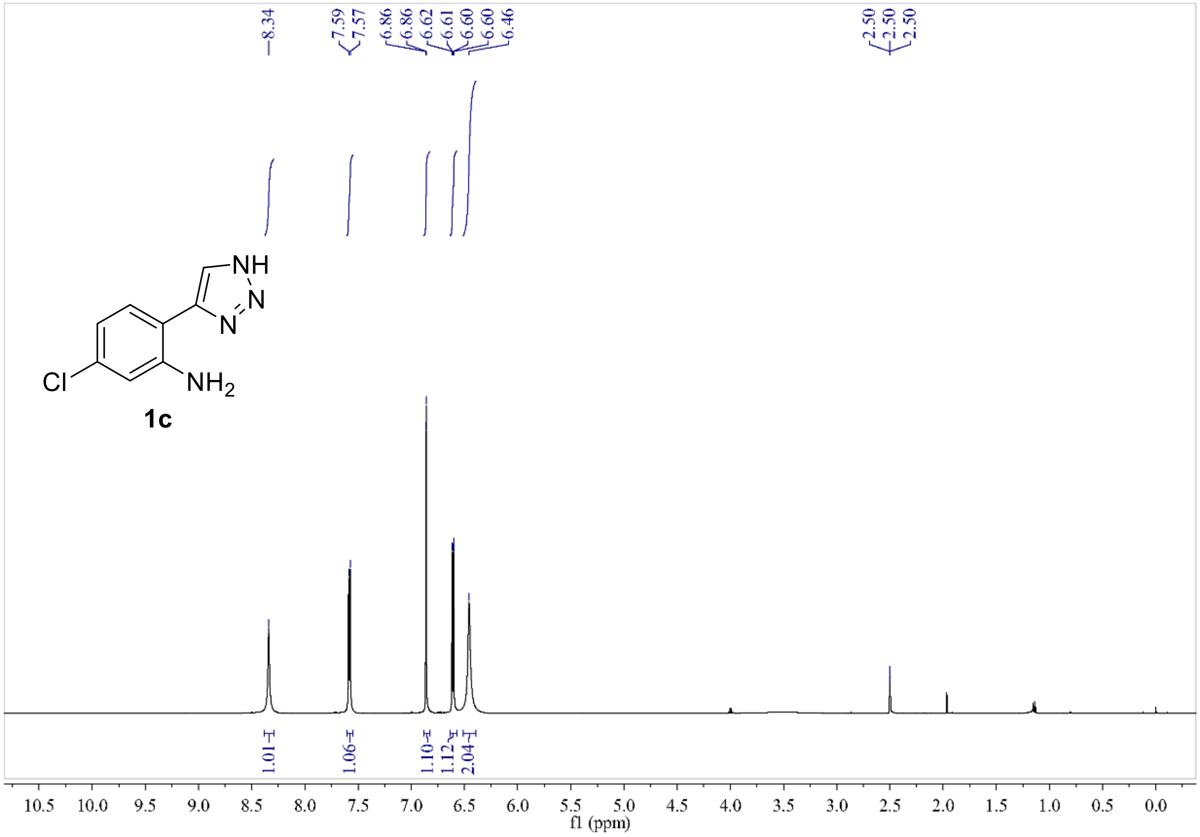

8.34
7.59
7.57
6.86
6.86
6.62
6.61
6.60
6.60
6.46
2.50
2.50
2.50
NH
N
N
Cl
NH2
1c
1.01
1.06
1.10
1.12
2.04
10.5 10.0 9.5 9.0 8.5 8.0 7.5 7.0 6.5 6.0 5.5 5.0 4.5 4.0 3.5 3.0 2.5 2.0 1.5 1.0 0.5 0.0
f1 (ppm)

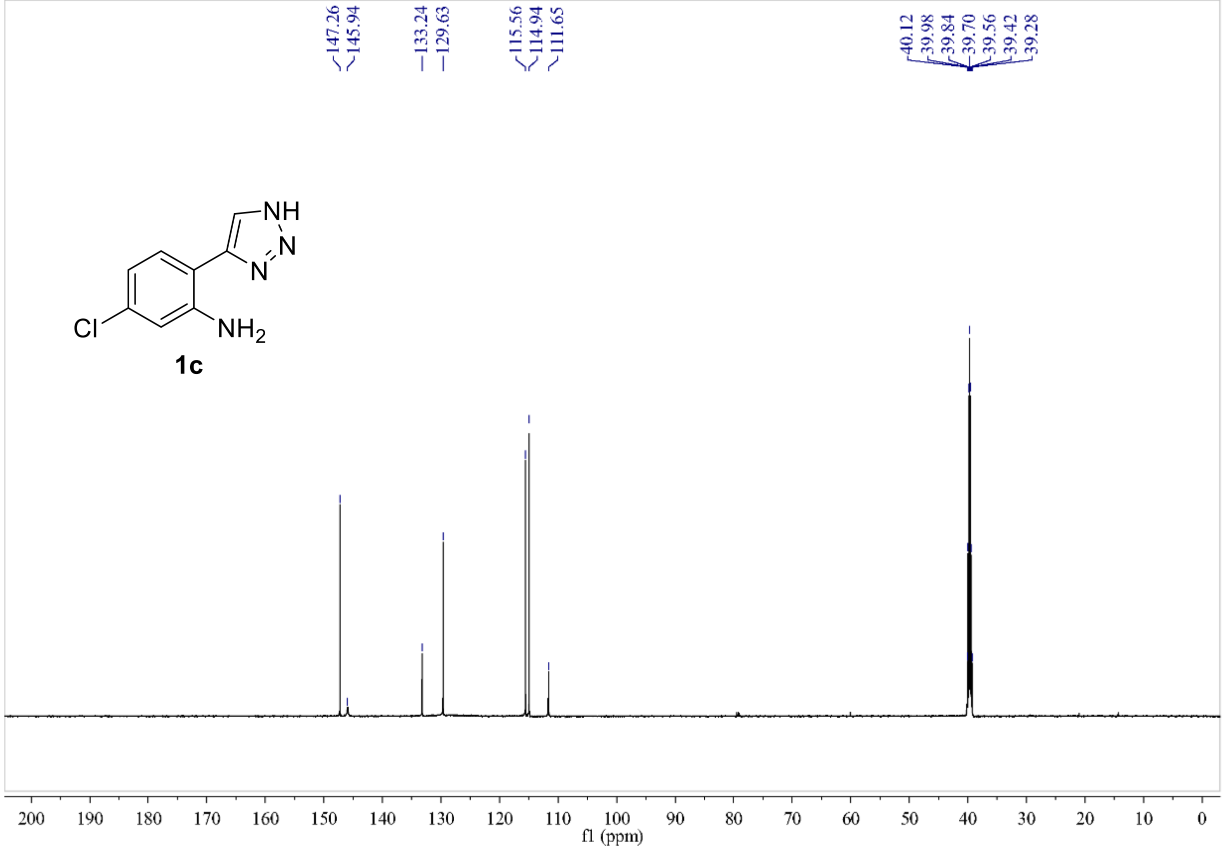

147.26
145.94
133.24
129.63
115.56
114.94
111.65
40.12
39.98
39.84
39.70
39.56
39.42
39.28
NH
N
N
Cl
NH2
1c
200 190 180 170 160 150 140 130 120 110 100 90 80 70 60 50 40 30 20 10 0
f1 (ppm)

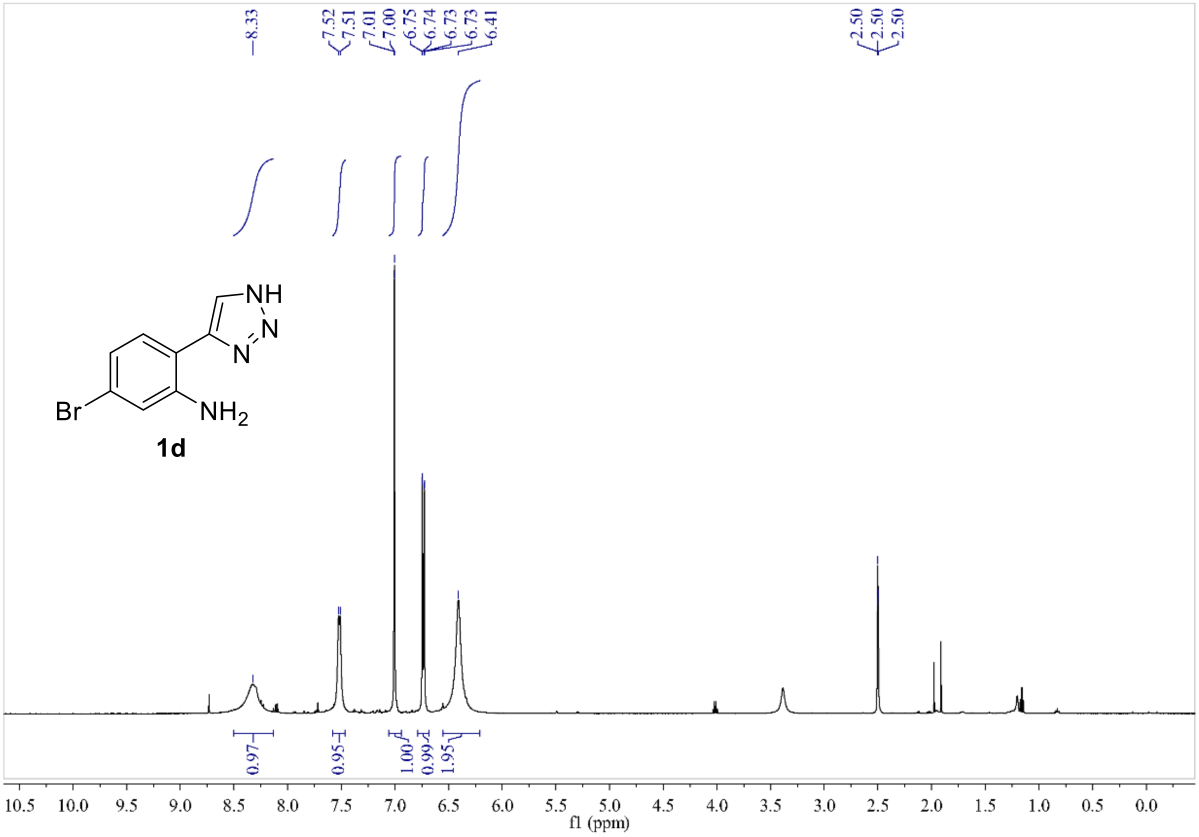
8.33
7.52
7.51
7.01
7.00
6.75
6.74
6.73
6.73
6.41
2.50
2.50
2.50
NH
N
N
Br
NH2
1d
0.97
0.95
1.00
0.99
1.95
10.5 10.0 9.5 9.0 8.5 8.0 7.5 7.0 6.5 6.0 5.5 5.0 4.5 4.0 3.5 3.0 2.5 2.0 1.5 1.0 0.5 0.0
f1 (ppm)

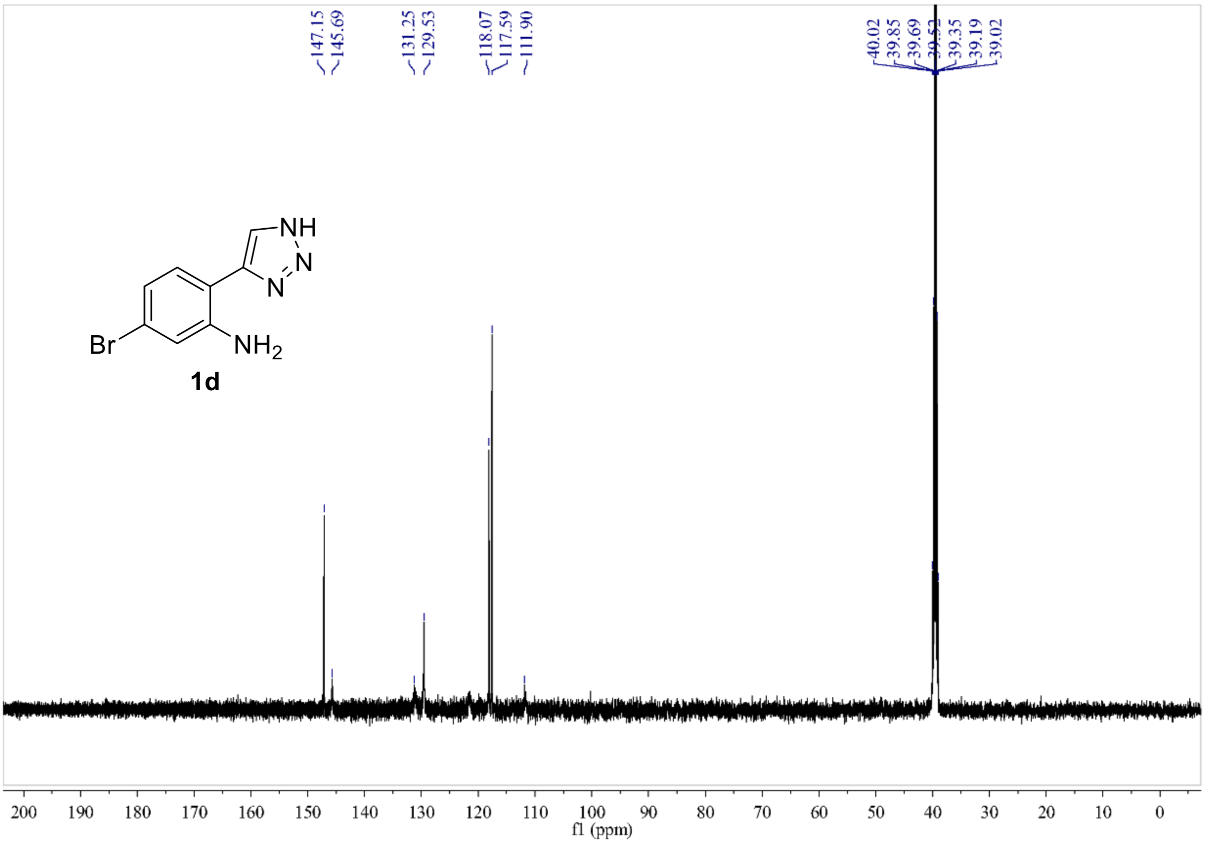
147.15
145.69
131.25
129.53
118.07
117.59
111.90
40.02
39.85
39.69
39.52
39.35
39.19
39.02
NH
N
N
Br
NH2
1d
200 190 180 170 160 150 140 130 120 110 100 90 80 70 60 50 40 30 20 10 0
f1 (ppm)

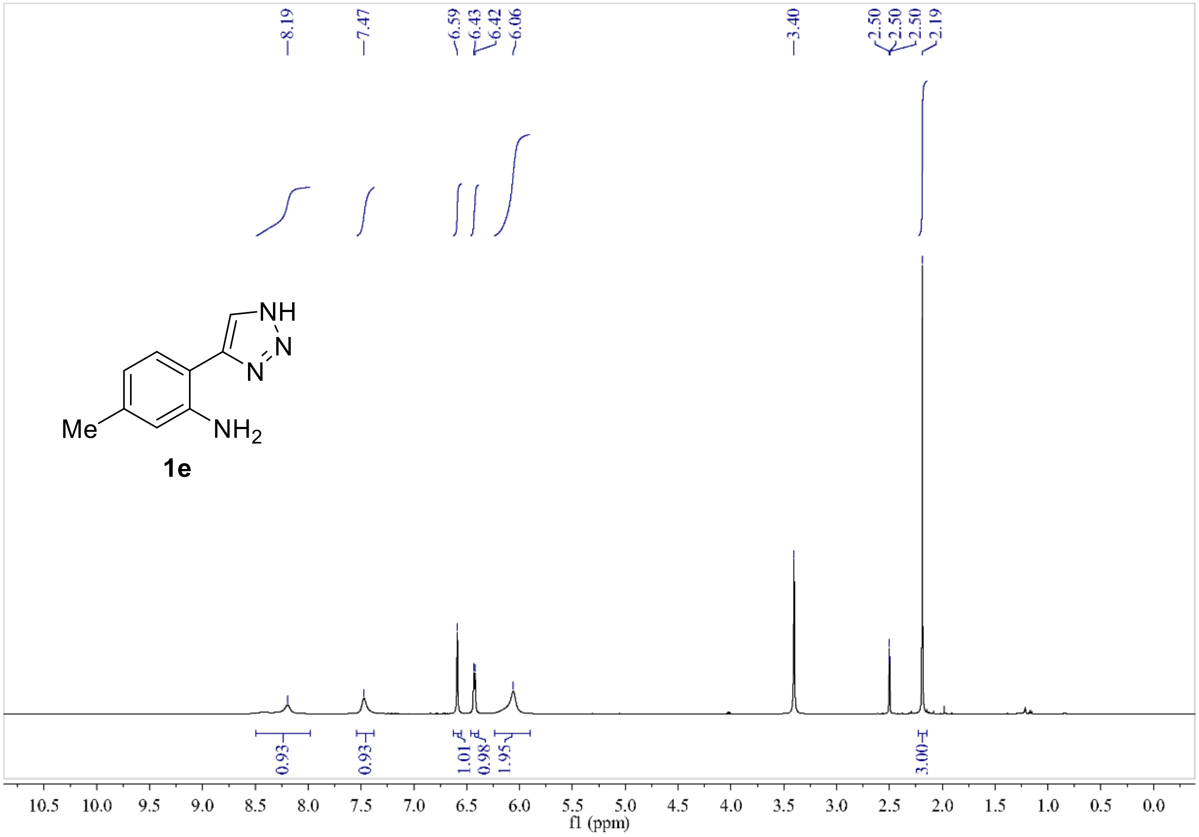
8.19
7.47
6.59
6.43
6.42
6.06
3.40
2.50
2.50
2.50
2.19
NH
N
N
Me
NH2
1e
0.93
0.93
1.01
0.98
1.95
3.00
10.5 10.0 9.5 9.0 8.5 8.0 7.5 7.0 6.5 6.0 5.5 5.0 4.5 4.0 3.5 3.0 2.5 2.0 1.5 1.0 0.5 0.0
f1 (ppm)

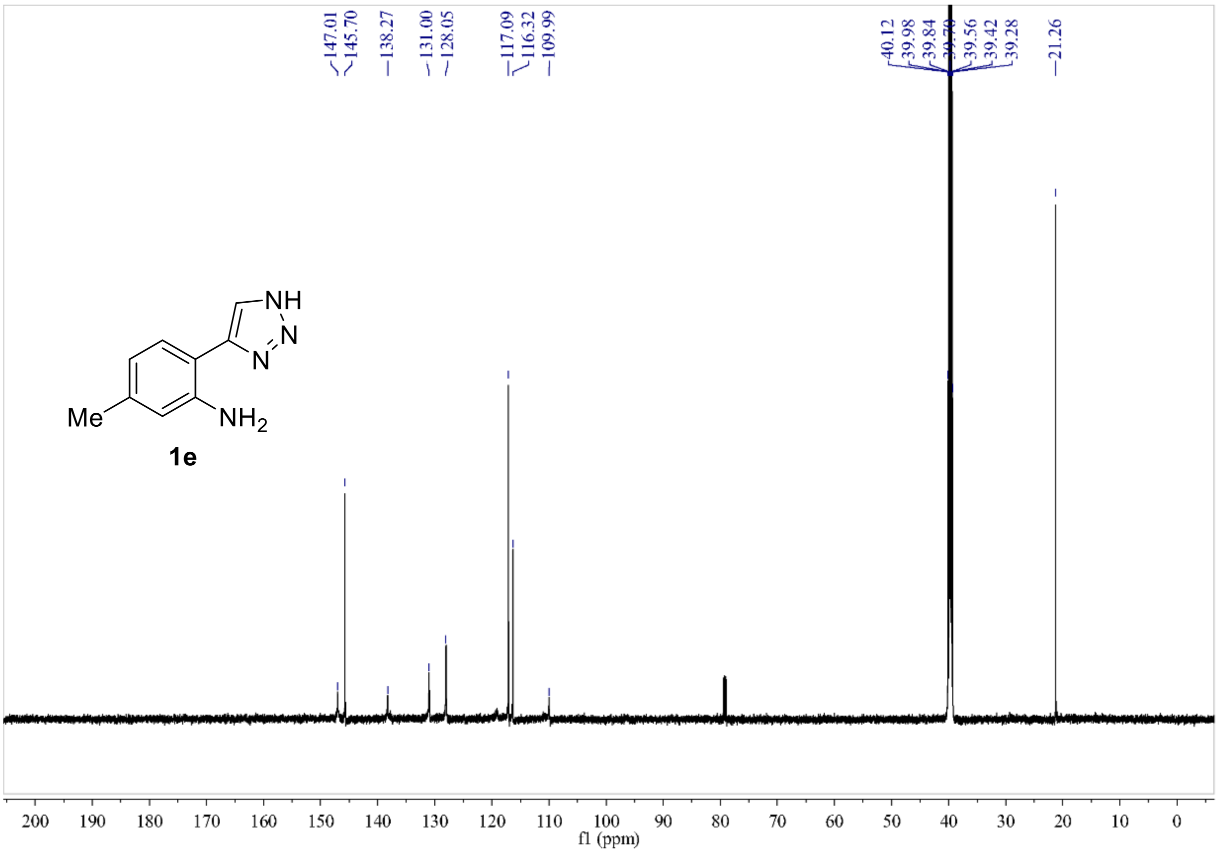
147.01
145.70
138.27
131.00
128.05
117.09
116.32
109.99
40.12
39.98
39.84
39.56
39.42
39.28
21.26
NH
N
N
Me
NH2
1e
200 190 180 170 160 150 140 130 120 110 100 90 80 70 60 50 40 30 20 10 0
f1 (ppm)

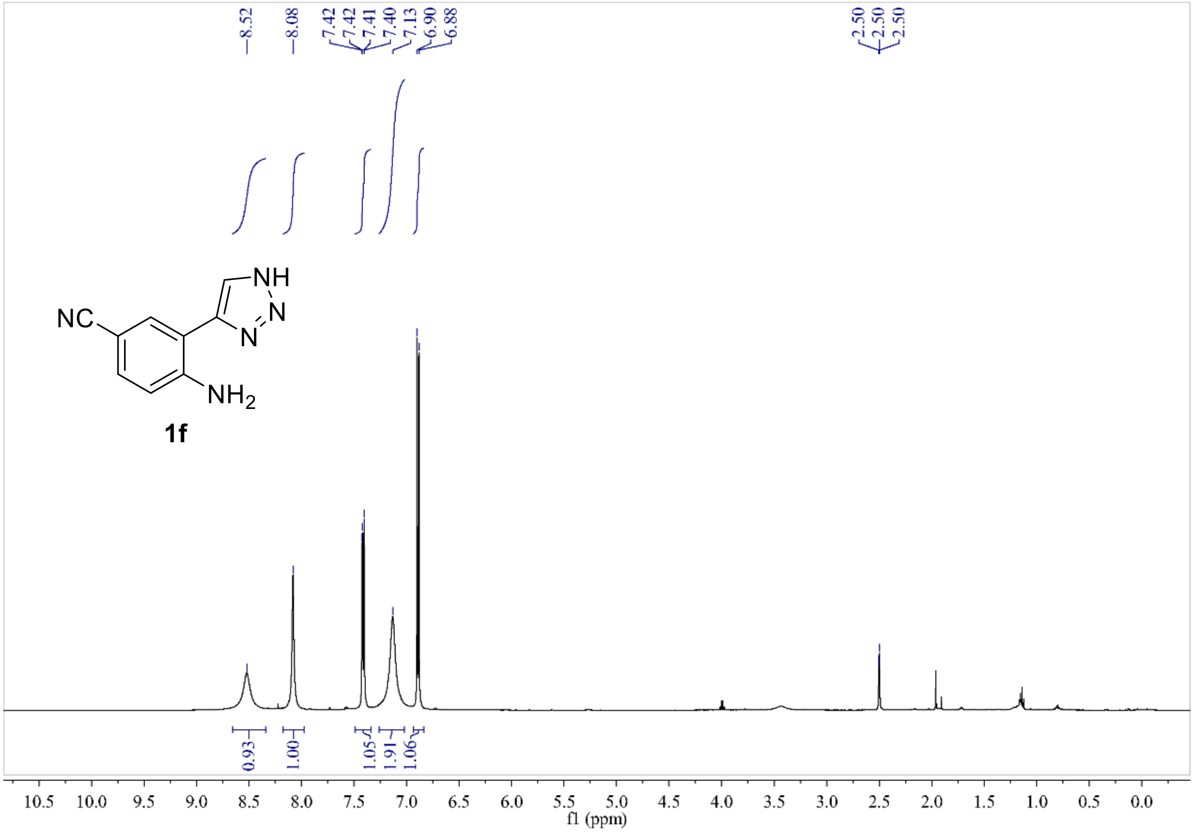
8.52
8.08
7.42
7.42
7.41
7.40
7.13
6.90
6.88
2.50
2.50
2.50
NC
NH
N
N
NH2
1f
0.93
1.00
1.05
1.91
1.06
10.5 10.0 9.5 9.0 8.5 8.0 7.5 7.0 6.5 6.0 5.5 5.0 4.5 4.0 3.5 3.0 2.5 2.0 1.5 1.0 0.5 0.0
f1 (ppm)

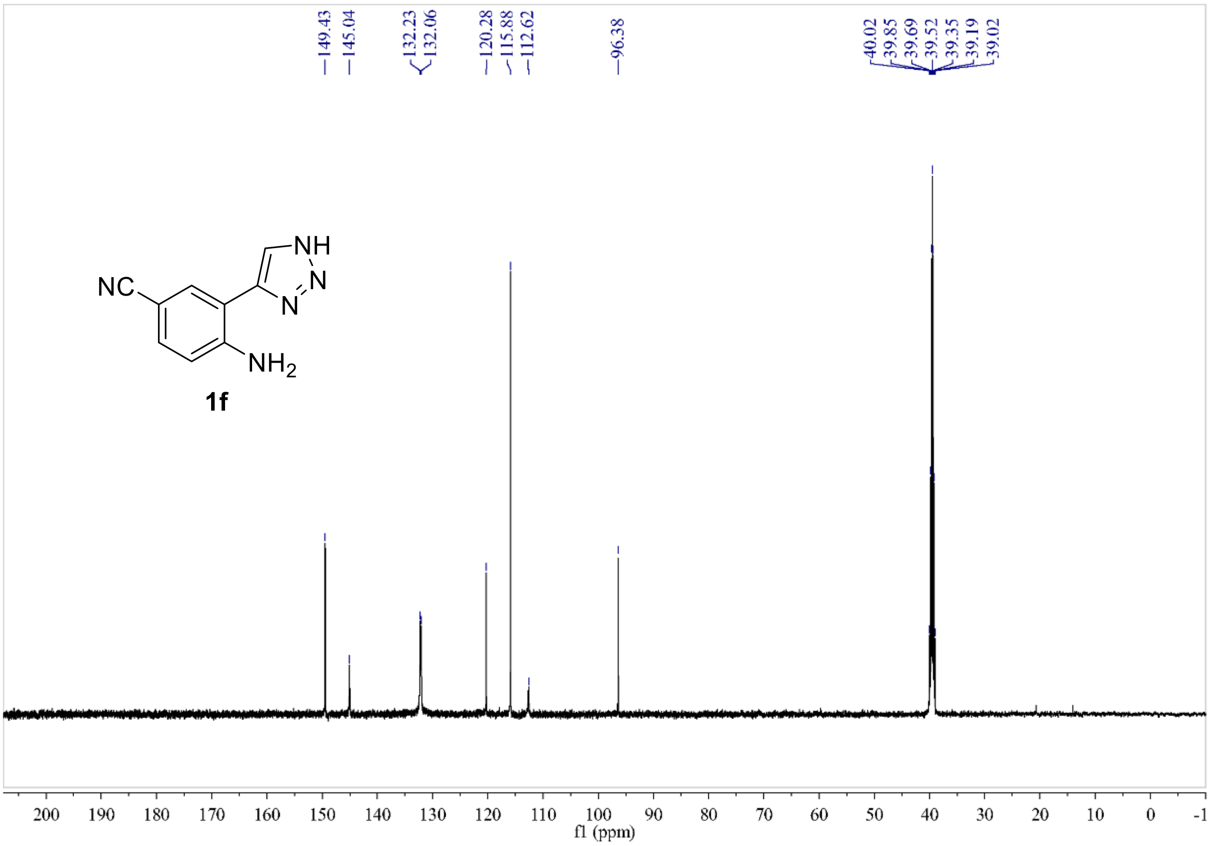
149.43
145.04
132.23
132.06
120.28
115.88
112.62
96.38
40.02
39.85
39.69
39.52
39.35
39.19
39.02
NC
NH
N
N
NH2
1f
200 190 180 170 160 150 140 130 120 110 100 90 80 70 60 50 40 30 20 10 0 -1
f1 (ppm)

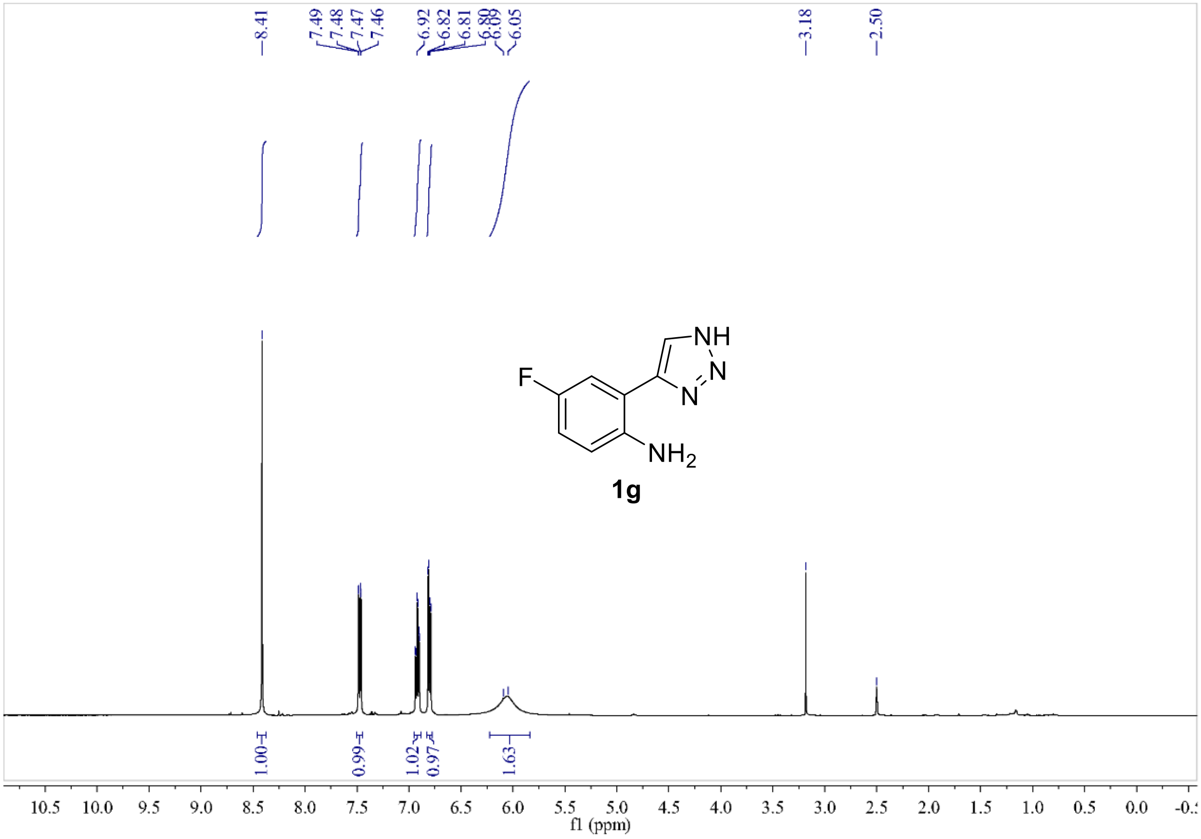
8.41
7.49
7.48
7.47
7.46
6.92
6.82
6.81
6.80
6.09
6.05
3.18
2.50
F
NH
N
N
NH2
1g
1.00
0.99
1.02
0.97
1.63
10.5 10.0 9.5 9.0 8.5 8.0 7.5 7.0 6.5 6.0 5.5 5.0 4.5 4.0 3.5 3.0 2.5 2.0 1.5 1.0 0.5 0.0
f1 (ppm)

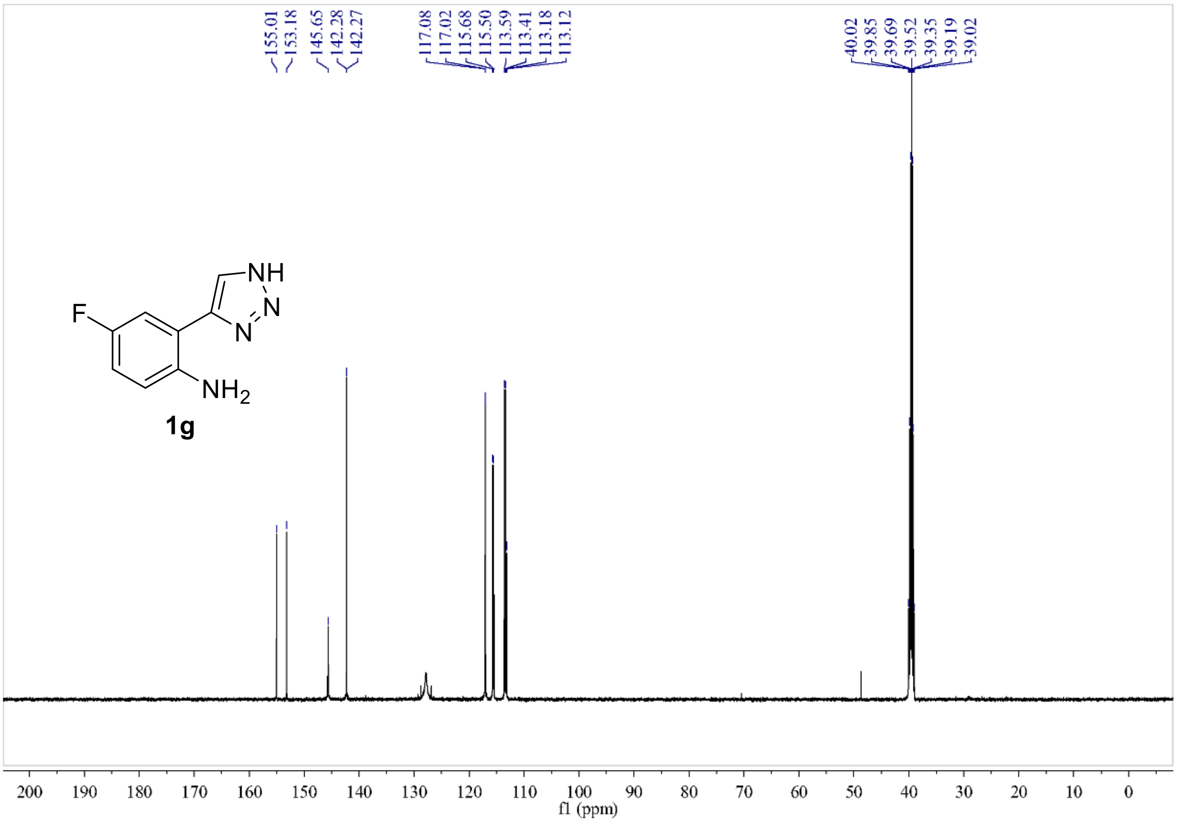
155.01
153.18
145.65
142.28
142.27
117.08
117.02
115.68
115.50
113.59
113.41
113.18
113.12
40.02
39.85
39.69
39.52
39.35
39.19
39.02
F
NH
N
N
NH2
1g
200 190 180 170 160 150 140 130 120 110 100 90 80 70 60 50 40 30 20 10 0
f1 (ppm)

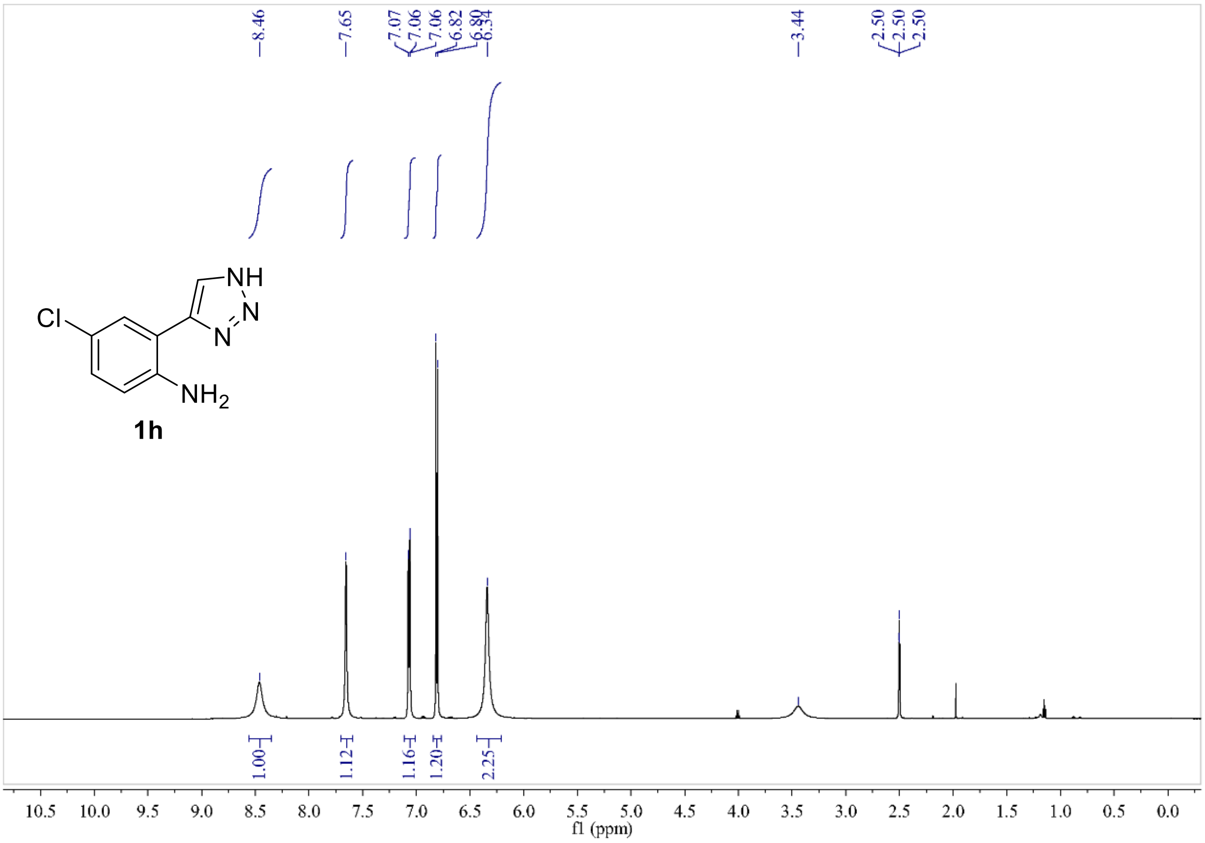
8.46
7.65
7.07
7.06
7.06
6.82
6.80
6.34
3.44
2.50
2.50
2.50
Cl
NH
N
N
NH2
1h
1.00
1.12
1.16
1.20
2.25
10.5 10.0 9.5 9.0 8.5 8.0 7.5 7.0 6.5 6.0 5.5 5.0 4.5 4.0 3.5 3.0 2.5 2.0 1.5 1.0 0.5 0.0
f1 (ppm)

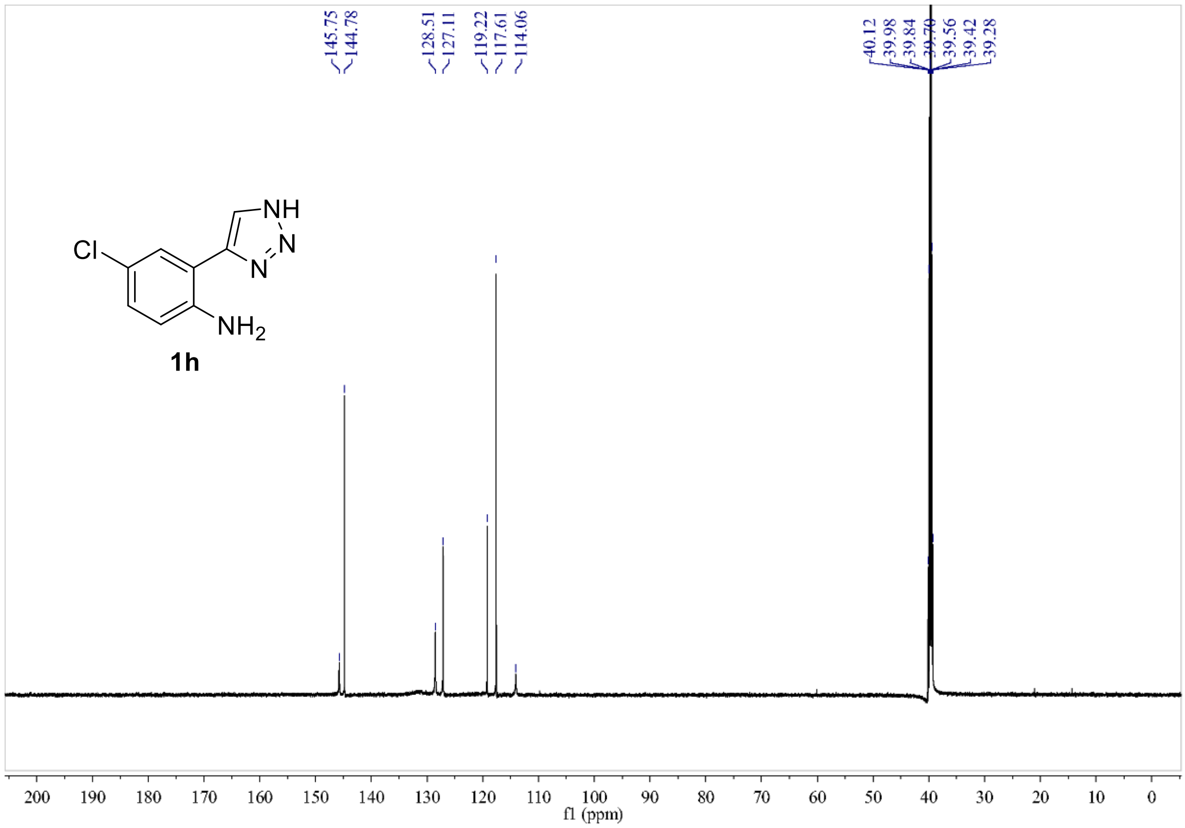
145.75
144.78
128.51
127.11
119.22
117.61
114.06
40.12
39.98
39.84
39.70
39.56
39.42
39.28
Cl
NH
N
N
NH2
1h
200 190 180 170 160 150 140 130 120 110 100 90 80 70 60 50 40 30 20 10 0
f1 (ppm)

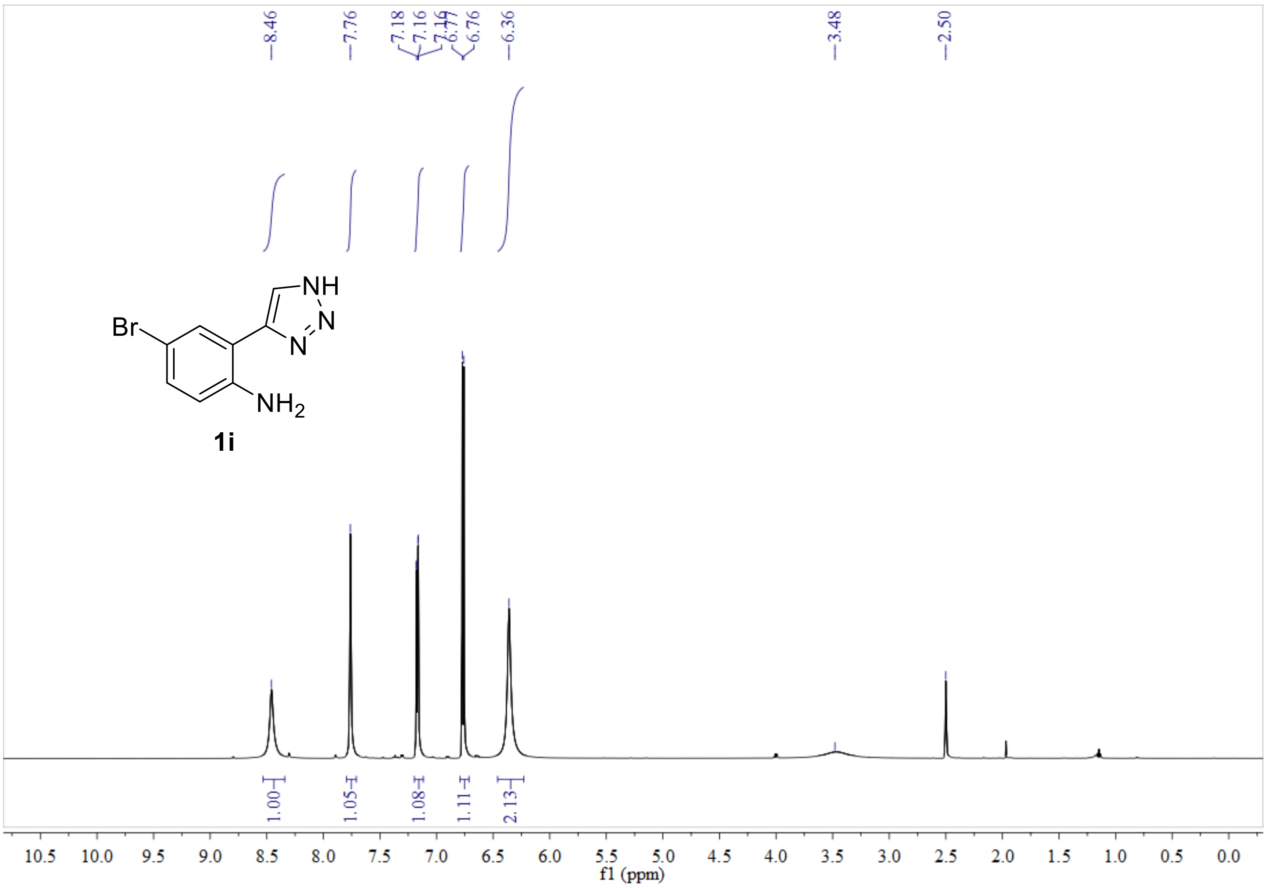
8.46
7.76
7.18
7.16
6.76
6.36
3.48
2.50
Br
NH
N
N
NH2
1i
1.00
1.05
1.08
1.11
2.13
f1 (ppm)

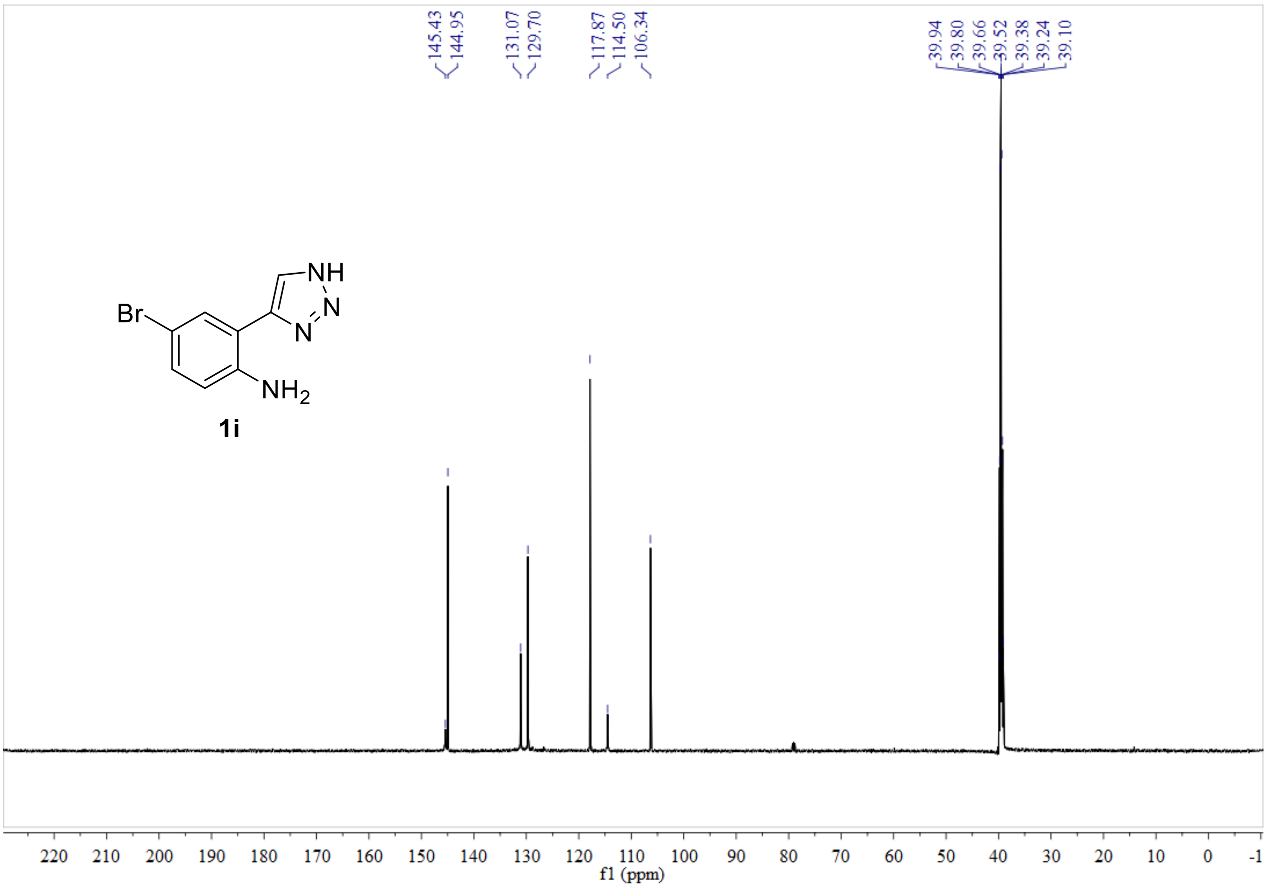
145.43
144.95
131.07
129.70
117.87
114.50
106.34
39.94
39.80
39.66
39.52
39.38
39.24
39.10
Br
NH
N
N
NH2
1i
f1 (ppm)

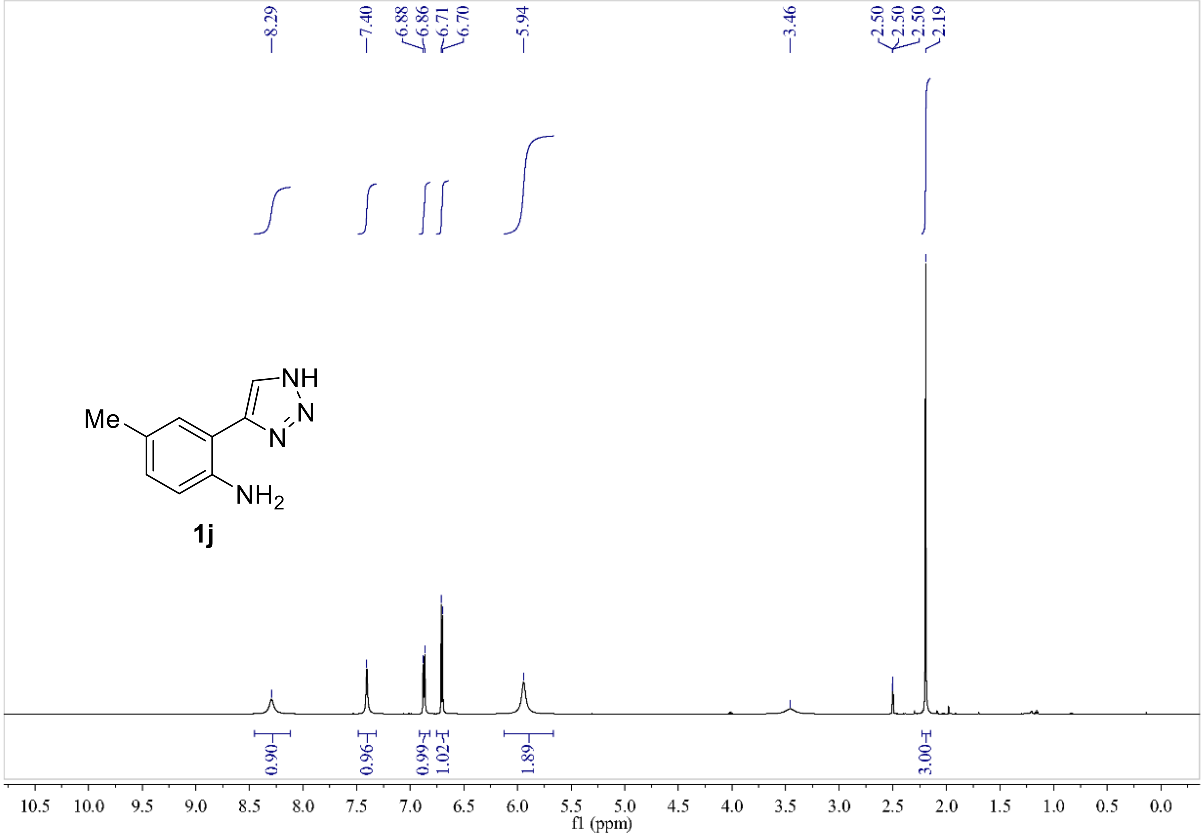
8.29
7.40
6.88
6.86
6.71
6.70
5.94
3.46
2.50
2.50
2.50
2.19
Me
NH
N
N
NH2
1j
0.90
0.96
0.99
1.02
1.89
3.00
10.5 10.0 9.5 9.0 8.5 8.0 7.5 7.0 6.5 6.0 5.5 5.0 4.5 4.0 3.5 3.0 2.5 2.0 1.5 1.0 0.5 0.0
f1 (ppm)

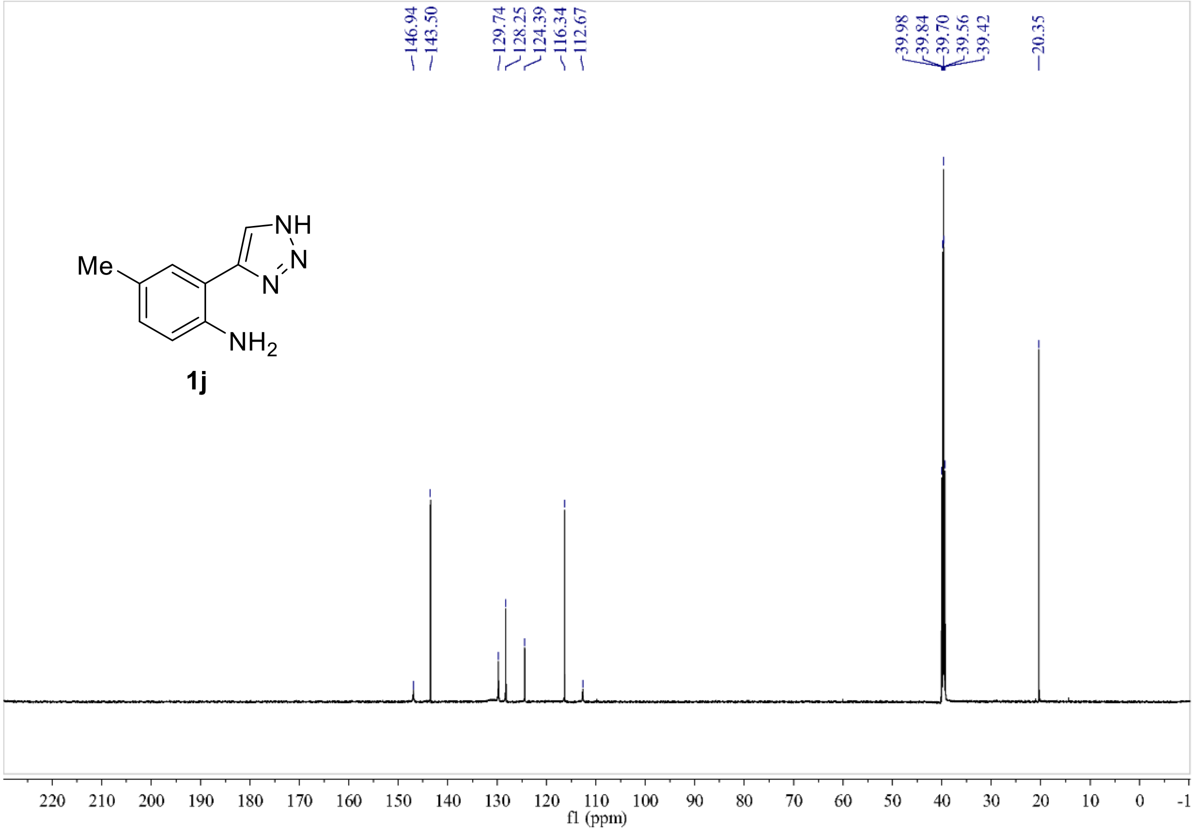
146.94
143.50
129.74
128.25
124.39
116.34
112.67
39.98
39.84
39.70
39.56
39.42
20.35
Me
NH
N
N
NH2
1j
220 210 200 190 180 170 160 150 140 130 120 110 100 90 80 70 60 50 40 30 20 10 0 -1
f1 (ppm)

6. Crystallographic date of 3aa

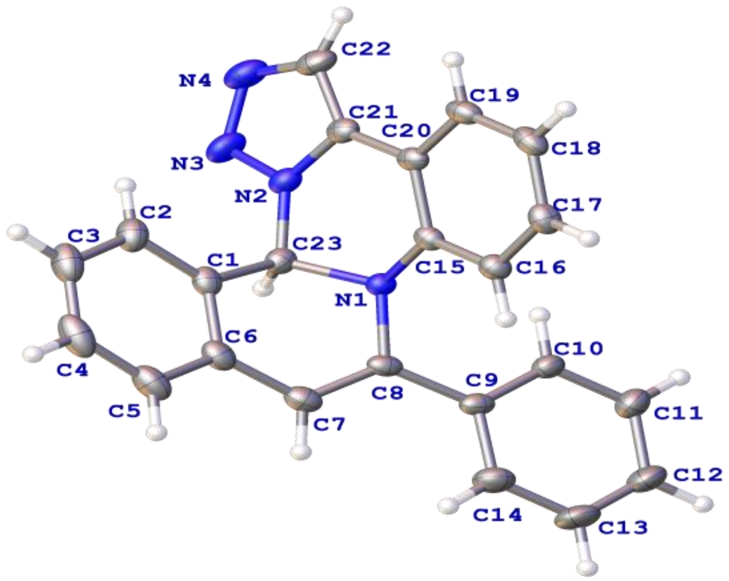

Datablock: 3aa

Bond precision:	C-C = 0.0030 A		Wavelength=0.71073
Cell:	a=12.3967(13)	b=24.631(3)	c=7.5204(7)
	alpha=90	beta=99.062(9)	gamma=90
Temperature:	200 K		

	Calculated	Reported
Volume	2267.6(4)	2267.6(4)
Space group	P 21/c	P 1 21/c 1
Hall group	-P 2ybc	-P 2ybc
Moiety formula	C23 H16 N4 [+ solvent]	C23 H16 N4
Sum formula	C23 H16 N4 [+ solvent]	C23 H16 N4
Mr	348.40	348.40
Dx,g cm-3	1.020	1.020
Z	4	4
Mu (mm-1)	0.062	0.062
F000	728.0	728.0
F000′	728.24	
h,k,lmax	14,29,8	14,29,8
Nref	3996	3991
Tmin,Tmax	0.991,0.993	0.409,1.000

Tmin' 0.991

Correction method= # Reported T Limits: Tmin=0.409 Tmax=1.000
AbsCorr = MULTI-SCAN

Data completeness= 0.999 Theta(max)= 24.996

R(reflections)= 0.0555(2905) wR2(reflections)=0.1521(3991)

S = 1.016 Npar= 244

Table 1 Crystal data and structure refinement for 3aa.

Identification code	3aa
Empirical formula	$C_{23}H_{16}N_4$
Formula weight	348.40
Temperature/K	200.00(10)
Crystal system	monoclinic
Space group	$P2_1/c$
a/Å	12.3967(13)
b/Å	24.631(3)
c/Å	7.5204(7)
α/°	90
β/°	99.062(9)
γ/°	90

Volume/Å^3	2267.6(4)
Z	4
ρ_{calc}g/cm^3	1.020
μ/mm^{-1}	0.062
F(000)	728.0
Crystal size/mm^3	0.15 × 0.14 × 0.12
Radiation	Mo Kα (λ = 0.71073)
2Θ range for data collection/°	4.692 to 49.992
Index ranges	-14 ≤ h ≤ 14, -24 ≤ k ≤ 29, -8 ≤ l ≤ 8
Reflections collected	9714
Independent reflections	3993 [R_{int} = 0.0363, R_{sigma} = 0.0497]
Data/restraints/parameters	3993/0/244
Goodness-of-fit on F^2	1.010
Final R indexes [I>=2σ (I)]	R_1 = 0.0564, wR_2 = 0.1459
Final R indexes [all data]	R_1 = 0.0749, wR_2 = 0.1587
Largest diff. peak/hole / e Å^{-3}	0.20/-0.22

Crystal structure determination of 3aa

Crystal Data for $C_{23}H_{16}N_4$ (M =348.40 g/mol): monoclinic, space group P2$_1$/c (no. 14), a = 12.3967(13) Å, b = 24.631(3) Å, c = 7.5204(7) Å, β = 99.062(9)°, V = 2267.6(4) Å^3, Z =

4, T = 200.00(10) K, μ(Mo Kα) = 0.062 mm^{-1}, $Dcalc$ = 1.020 g/cm^3, 9714 reflections measured (4.692° ≤ 2Θ ≤ 49.992°), 3993 unique (R_{int} = 0.0363, R_{sigma} = 0.0497) which were used in all calculations. The final R_1 was 0.0564 (I > 2σ(I)) and wR_2 was 0.1587 (all data).

Table 2 Fractional Atomic Coordinates (×10⁴) and Equivalent Isotropic Displacement Parameters (Å²×10³) for 3aa. U_{eq} is defined as 1/3 of of the trace of the orthogonalised U_{IJ} tensor.

**Atom**	*x*	*y*	*z*	**U(eq)**
N1	6274.5(12)	6402.5(6)	2411.8(19)	30.2(4)
N2	4387.0(13)	6538.5(7)	1688(2)	36.7(4)
N3	3482.6(14)	6610.5(8)	445(3)	51.6(5)
N4	2839.1(15)	6934.3(9)	1203(3)	57.7(5)
C1	5111.0(17)	5599.0(8)	2000(2)	36.9(5)
C2	4149.9(19)	5377.3(9)	2367(3)	48.2(6)
C3	4131(2)	4827.7(11)	2834(3)	58.8(7)
C4	5045(3)	4515.6(10)	2894(3)	62.7(8)
C5	6012(2)	4738.0(9)	2506(3)	53.3(6)
C6	6060.5(18)	5286.3(9)	2092(2)	40.8(5)
C7	7061.5(17)	5553.1(9)	1816(3)	41.1(5)
C8	7202.1(15)	6092.7(8)	2057(2)	32.8(5)
C9	8211.3(15)	6396.7(8)	1936(2)	35.2(5)

Atom	*x*	*y*	*z*	U(eq)
C10	8202.1(15)	6958.4(9)	1849(3)	37.9(5)
C11	9135.8(17)	7248.3(11)	1637(3)	51.3(6)
C12	10092.9(18)	6976.0(12)	1523(4)	60.3(7)
C13	10125.3(19)	6416.0(12)	1645(4)	65.7(7)
C14	9196.3(18)	6130.8(10)	1859(3)	53.1(6)
C15	6246.3(15)	6559.9(7)	4215(2)	29.2(4)
C16	7147.2(16)	6525.4(8)	5558(2)	35.7(5)
C17	7089.8(18)	6717.9(8)	7272(3)	41.1(5)
C18	6148.3(19)	6946.9(8)	7695(3)	43.7(5)
C19	5246.8(18)	6972.1(8)	6389(3)	40.5(5)
C20	5273.1(15)	6784.7(7)	4645(3)	32.5(5)
C21	4338.3(16)	6810.2(8)	3229(3)	36.1(5)
C22	3335.3(17)	7060.0(10)	2891(3)	49.5(6)
C23	5264.0(15)	6175.8(8)	1421(2)	34.7(5)

Table 3 Anisotropic Displacement Parameters (Å^2×10^3) for 3aa. The Anisotropic displacement factor exponent takes the form: $-2\pi^2[h^2a^{*2}U_{11}+2hka^*b^*U_{12}+\ldots]$.

Atom	U_{11}	U_{22}	U_{33}	U_{23}	U_{13}	U_{12}
N1	28.2(9)	32.2(9)	30.2(8)	-0.1(7)	4.6(6)	0.8(7)

Atom	U_{11}	U_{22}	U_{33}	U_{23}	U_{13}	U_{12}
N2	29.2(9)	39.6(10)	41.0(9)	5.6(8)	5.3(7)	-1.4(7)
N3	29.9(10)	68.3(14)	54.3(11)	6.2(10)	-0.9(9)	-0.7(9)
N4	34.3(11)	67.8(14)	70.9(13)	10.4(11)	8.6(10)	3.8(10)
C1	47.4(13)	36.5(12)	26.3(9)	-2.0(9)	4.5(8)	-9.4(10)
C2	56.8(14)	47.2(14)	40.7(11)	-1.4(11)	8.3(10)	-16.7(11)
C3	81.7(19)	50.9(16)	44.3(13)	-2.6(12)	11.2(12)	-28.8(14)
C4	112(2)	37.0(14)	37.6(12)	2.9(11)	6.4(14)	-20.4(16)
C5	83.4(18)	33.4(13)	42.1(12)	-0.1(10)	6.6(12)	-0.1(12)
C6	58.2(14)	33.4(12)	29.9(10)	-1.6(9)	3.8(9)	-1.3(10)
C7	50.1(13)	38.8(13)	34.6(10)	-2.6(10)	7.5(9)	7.9(10)
C8	35.1(11)	39.1(12)	24.1(9)	-2.8(9)	4.1(8)	9.5(9)
C9	31.8(11)	44.7(13)	28.9(9)	-3.8(9)	4.6(8)	7.6(9)
C10	28.6(11)	44.5(13)	40.9(11)	-4.5(10)	5.9(8)	0.2(9)
C11	37.5(13)	57.2(16)	59.0(13)	-5.1(12)	7.0(10)	-7.1(11)
C12	31.3(13)	73.8(19)	78.1(17)	-10.8(15)	16.2(11)	-2.6(12)
C13	33.1(14)	80(2)	85.7(19)	-10.8(16)	14.1(12)	12.5(13)
C14	43.8(14)	55.0(15)	61.1(14)	-1.4(12)	10.3(11)	12.9(11)
C15	37.7(11)	23.0(10)	27.9(9)	3.0(8)	8.3(8)	-0.6(8)
C16	40.2(12)	32.7(11)	34.3(10)	0.6(9)	6.6(9)	0.3(9)
C17	52.7(13)	37.5(12)	31.5(10)	0.7(9)	1.5(9)	-3.9(10)

Atom	U_{11}	U_{22}	U_{33}	U_{23}	U_{13}	U_{12}
C18	63.2(15)	32.8(12)	38.2(11)	-6.4(10)	18.0(11)	-5.8(10)
C19	51.1(13)	28.1(11)	47.2(12)	-0.1(10)	22.4(10)	1.6(9)
C20	36.4(11)	22.9(10)	40.3(10)	5.2(9)	13.0(9)	0.1(8)
C21	35.4(12)	30.0(11)	45.3(11)	5.9(10)	14.2(9)	-2.9(9)
C22	34.1(12)	53.2(15)	64.1(14)	9.1(12)	16.8(11)	2.3(10)
C23	33.9(11)	40.2(12)	29.3(9)	1.1(9)	3.1(8)	-2.2(9)

Table 4 Bond Lengths for 3aa.

Atom	Atom	Length/Å	Atom	Atom	Length/Å
N1	C8	1.440(2)	C7	C8	1.349(3)
N1	C15	1.416(2)	C8	C9	1.473(3)
N1	C23	1.464(2)	C9	C10	1.385(3)
N2	N3	1.354(2)	C9	C14	1.395(3)
N2	C21	1.348(3)	C10	C11	1.390(3)
N2	C23	1.445(3)	C11	C12	1.377(3)
N3	N4	1.319(3)	C12	C13	1.383(4)
N4	C22	1.357(3)	C13	C14	1.380(3)
C1	C2	1.377(3)	C15	C16	1.386(3)
C1	C6	1.399(3)	C15	C20	1.411(3)

Atom	Atom	Length/Å	Atom	Atom	Length/Å
C1	C23	1.507(3)	C16	C17	1.386(3)
C2	C3	1.400(3)	C17	C18	1.378(3)
C3	C4	1.364(4)	C18	C19	1.368(3)
C4	C5	1.390(4)	C19	C20	1.396(3)
C5	C6	1.389(3)	C20	C21	1.446(3)
C6	C7	1.448(3)	C21	C22	1.375(3)

Table 5 Bond Angles for 3aa.

Atom	Atom	Atom	Angle/˚	Atom	Atom	Atom	Angle/˚
C8	N1	C23	110.38(14)	C14	C9	C8	121.44(19)
C15	N1	C8	117.62(14)	C9	C10	C11	121.17(19)
C15	N1	C23	116.34(14)	C12	C11	C10	119.9(2)
N3	N2	C23	122.99(16)	C11	C12	C13	119.9(2)
C21	N2	N3	112.60(17)	C14	C13	C12	120.0(2)
C21	N2	C23	124.25(16)	C13	C14	C9	121.2(2)
N4	N3	N2	105.81(17)	C16	C15	N1	122.87(16)
N3	N4	C22	109.05(18)	C16	C15	C20	118.50(16)
C2	C1	C6	121.3(2)	C20	C15	N1	118.57(16)
C2	C1	C23	125.7(2)	C17	C16	C15	120.24(19)
C6	C1	C23	112.97(17)	C18	C17	C16	121.62(19)

Atom	Atom	Atom	Angle/°	Atom	Atom	Atom	Angle/°
C1	C2	C3	118.9(2)	C19	C18	C17	118.61(18)
C4	C3	C2	120.5(2)	C18	C19	C20	121.47(19)
C3	C4	C5	120.7(2)	C15	C20	C21	117.58(16)
C4	C5	C6	119.9(2)	C19	C20	C15	119.53(18)
C1	C6	C7	118.44(19)	C19	C20	C21	122.88(18)
C5	C6	C1	118.7(2)	N2	C21	C20	118.18(17)
C5	C6	C7	122.8(2)	N2	C21	C22	103.00(19)
C8	C7	C6	121.58(19)	C22	C21	C20	138.8(2)
N1	C8	C9	116.94(16)	N4	C22	C21	109.5(2)
C7	C8	N1	117.18(18)	N1	C23	C1	110.43(15)
C7	C8	C9	125.85(17)	N2	C23	N1	107.21(15)
C10	C9	C8	120.66(17)	N2	C23	C1	114.27(16)
C10	C9	C14	117.89(19)				

Table 6 Torsion Angles for 3aa.

A	B	C	D	Angle/°	A	B	C	D	Angle/°
N1	C8	C9	C10	-11.1(2)	C9	C10	C11	C12	-0.5(3)
N1	C8	C9	C14	170.56(17)	C10	C9	C14	C13	-2.1(3)

A	B	C	D	Angle/°	A	B	C	D	Angle/°
N1	C15	C16	C17	175.86(18)	C10	C11	C12	C13	-0.9(4)
N1	C15	C20	C19	-176.24(17)	C11	C12	C13	C14	0.7(4)
N1	C15	C20	C21	3.6(3)	C12	C13	C14	C9	0.8(4)
N2	N3	N4	C22	-0.2(2)	C14	C9	C10	C11	1.9(3)
N2	C21	C22	N4	-0.4(2)	C15	N1	C8	C7	102.0(2)
N3	N2	C21	C20	-177.64(16)	C15	N1	C8	C9	-79.7(2)
N3	N2	C21	C22	0.3(2)	C15	N1	C23	N2	48.6(2)
N3	N2	C23	N1	149.46(17)	C15	N1	C23	C1	-76.5(2)
N3	N2	C23	C1	-87.8(2)	C15	C16	C17	C18	0.0(3)
N3	N4	C22	C21	0.4(3)	C15	C20	C21	N2	11.1(3)
C1	C2	C3	C4	-1.1(3)	C15	C20	C21	C22	-165.8(2)
C1	C6	C7	C8	21.9(3)	C16	C15	C20	C19	0.9(3)
C2	C1	C6	C5	2.1(3)	C16	C15	C20	C21	-179.20(17)
C2	C1	C6	C7	-175.69(17)	C16	C17	C18	C19	1.4(3)
C2	C1	C23	N1	135.60(19)	C17	C18	C19	C20	-1.6(3)
C2	C1	C23	N2	14.7(3)	C18	C19	C20	C15	0.5(3)
C2	C3	C4	C5	0.4(3)	C18	C19	C20	C21	-179.38(18)
C3	C4	C5	C6	1.5(3)	C19	C20	C21	N2	-169.04(18)
C4	C5	C6	C1	-2.8(3)	C19	C20	C21	C22	14.0(4)
C4	C5	C6	C7	174.93(19)	C20	C15	C16	C17	-1.2(3)

A	B	C	D	Angle/˚	A	B	C	D	Angle/˚
C5	C6	C7	C8	-155.8(2)	C20	C21	C22	N4	176.8(2)
C6	C1	C2	C3	-0.2(3)	C21	N2	N3	N4	-0.1(2)
C6	C1	C23	N1	-46.2(2)	C21	N2	C23	N1	-35.5(2)
C6	C1	C23	N2	-167.13(15)	C21	N2	C23	C1	87.2(2)
C6	C7	C8	N1	-6.5(3)	C23	N1	C8	C7	-34.7(2)
C6	C7	C8	C9	175.45(17)	C23	N1	C8	C9	143.51(16)
C7	C8	C9	C10	166.97(19)	C23	N1	C15	C16	147.38(18)
C7	C8	C9	C14	-11.4(3)	C23	N1	C15	C20	-35.6(2)
C8	N1	C15	C16	13.1(3)	C23	N2	N3	N4	175.50(17)
C8	N1	C15	C20	-169.81(16)	C23	N2	C21	C20	6.9(3)
C8	N1	C23	N2	-174.02(14)	C23	N2	C21	C22	-175.23(17)
C8	N1	C23	C1	60.92(19)	C23	C1	C2	C3	177.89(18)
C8	C9	C10	C11	-176.49(18)	C23	C1	C6	C5	-176.18(17)
C8	C9	C14	C13	176.3(2)	C23	C1	C6	C7	6.0(2)

Table 7 Hydrogen Atom Coordinates (Å×10^4) and Isotropic Displacement Parameters (Å^2×10^3) for 3aa.

Atom	*x*	*y*	*z*	U(eq)
H2	3524.07	5589.15	2304.92	58

Atom	*x*	*y*	*z*	U(eq)
H3	3491.07	4674.33	3105.15	71
H4	5020.68	4150.3	3198.44	75
H5	6625.74	4519.89	2522.15	64
H7	7620.88	5347.62	1464.38	49
H10	7560.05	7145.02	1934.62	46
H11	9114.12	7625.31	1572.63	62
H12	10716.22	7168.48	1365.05	72
H13	10772.88	6231.55	1582.16	79
H14	9227.47	5754.49	1954.11	64
H16	7792.82	6372.45	5308.48	43
H17	7701.86	6691.96	8158.35	49
H18	6126.1	7081.39	8844	52
H19	4601.96	7117.49	6669.94	49
H22	3041.97	7281.09	3696.3	59
H23	5322.29	6174.2	136.47	42

Table 8 Solvent masks information for 3aa.

Number	X	Y	Z	Volume	Electron count	Content
1	0.000	0.000	-0.068	377.8	92.9	?
2	0.000	0.500	-0.101	377.8	92.9	?

Experimental

Single crystals of $C_{23}H_{16}N_4$ **[3aa]** were **[]**. A suitable crystal was selected and **[]** on a **SuperNova, Dual, Cu at zero, AtlasS2** diffractometer. The crystal was kept at 200.00(10) K during data collection.
